# Synthesis and biological evaluation of novel *N*-(piperazin-1-yl)alkyl-1*H*-dibenzo[*a*,*c*]carbazole derivatives of dehydroabietic acid as potential MEK inhibitors

**DOI:** 10.1080/14756366.2019.1655407

**Published:** 2019-08-25

**Authors:** Hao Chen, Chao Qiao, Ting-Ting Miao, A-Liang Li, Wen-Yan Wang, Wen Gu

**Affiliations:** Jiangsu Provincial Key Lab for the Chemistry and Utilization of Agro-forest Biomass, Jiangsu Key Lab of Biomass-based Green Fuels and Chemicals, Co-Inovation Center for Efficient Processing and Utilization of Forest Products, College of Chemical Engineering, Nanjing Forestry University, Nanjing, PR China

**Keywords:** Dehydroabietic acid, anticancer activity, MEK inhibitor, oncosis, apoptosis

## Abstract

In this paper, a series of novel 1*H*-dibenzo[*a*,*c*]carbazole derivatives of dehydroabietic acid bearing different *N*-(piperazin-1-yl)alkyl side chains were designed, synthesised and evaluated for their *in vitro* anticancer activities against three human hepatocarcinoma cell lines (SMMC-7721, HepG2 and Hep3B). Among them, compound **10g** exhibited the most potent activity against three cancer cell lines with IC_50_ values of 1.39 ± 0.13, 0.51 ± 0.09 and 0.73 ± 0.08 µM, respectively. In the kinase inhibition assay, compound **10g** could significantly inhibit MEK1 kinase activity with IC_50_ of 0.11 ± 0.02 µM, which was confirmed by western blot analysis and molecular docking study. In addition, compound **10g** could elevate the intracellular ROS levels, decrease mitochondrial membrane potential, destroy the cell membrane integrity, and finally lead to the oncosis and apoptosis of HepG2 cells. Therefore, compound **10g** could be a potent MEK inhibitor and a promising anticancer agent worthy of further investigations.

## GRAPHICAL ABSTRACT

**Figure:**
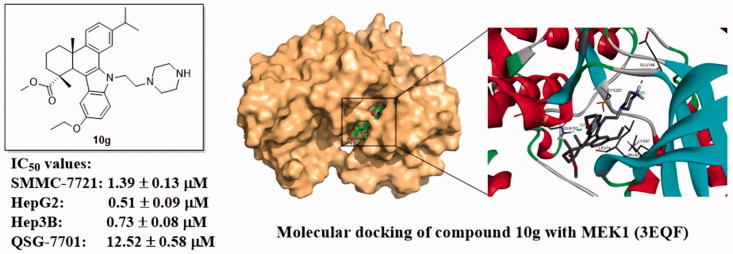

## 1. Introduction

Cancer has become the leading cause of human death worldwide and imposed tremendous health problem to human beings. Besides traditional chemotherapy agents, the exploration on signal transduction networks closely related to oncogenesis and cancer development, has led to a lot of targeted cancer therapeutics with prominent therapeutic benefits^1^. Among them, the mitogen-activated protein kinase (MAPK) pathway plays a central role in controlling mammalian cell functions, including adhesion, migration, differentiation, metabolism and proliferation^2^.

The MAPK pathway includes a chain of proteins that communicates the signal from a receptor on the cell surface to DNA in the nucleus^3^. The pathway is activated when an extracellular stimulus binds to its receptor, which results in activation of the membrane-bound GTPase (RAS) and then leads to the recruitment and activation of Raf, a serine-threonine kinase. Subsequently, the signal is transmitted downstream through activated Raf by phosphorylating and activating its main substrates MEK1/2, two dual-specific kinases which also activate their substrates ERK1/2 *via* phosphorylation of conserved threonine and tyrosine residues in the activation loop^4^^,^^5^. When activated, ERK1/2 in turn phosphorylates and activates several downstream proteins located in cytoplasm or nucleus, leading to a range of cellular events^6^^,^^7^. This pathway is also known as Ras-Raf-MEK-ERK pathway^8^, which is aberrantly activated in more than 30% of human cancers such as hepatocarcinoma (HCC), prostate carcinoma, non-small cell lung cancer (NSCLC), leukemia and melanoma^9^. Consequently, the inhibition of signal transduction through MAPK pathway can be a promising strategy for tumour targeted therapy.

**Figure 1.**
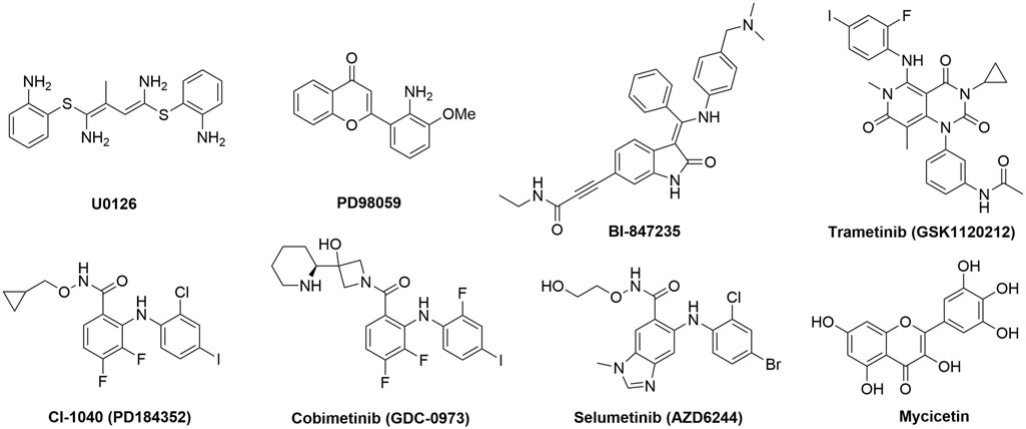
Examples of MEK kinase inhibitors.

As a key node of MAPK pathway, the Ser/Thr kinases MEK1/2 specifically phosphorylate and activate ERK1/2. The inhibition of MEK kinase activity will effectively impede the signal transduction of MAPK pathway. Hence, the interest in MEK1/2 has generated several small molecule inhibitors, e.g. highly specific MEK1/2 inhibitors such as U0126, PD98059, BI-847325, trametinib (GSK1120212), CI-1040 (PD184352), cobimetinib (GDC-0973), selumetinib (AZD6244) and myricetin (Figure 1)^10–17^. CI-1040 is an ATP non-competitive MEK1/2 inhibitor which directly inhibits MEK1 with a 50% inhibitory concentration (IC_50_) of 17 nM^18^. It is the first MEK inhibitor which entered clinical trials for treating a panel of advanced cancers. However, the phase II study results provided little support for further investigation of CI-1040 and the development was terminated^19^. Selumetimib (AZD6244) is an orally available, selective, ATP-noncompetitive MEK1/2 inhibitor which showed significant antitumour activity in cell lines harboring *BRAF* or *RAS* mutations^20^ and in various xenograft models^21^. In a phase II trial that compared selumetinib plus docetaxel with matching placebo plus docetaxel in patients with previously treated *KRAS*-mutant NSCLC, the median overall survival (OS) and progression-free survival (PFS) was significantly longer in the selumetinib plus docetaxel group^22^. However, in a follow-up randomised phase III study (SELECT-1), the results did not confirm the survival benefit of selumetinib plus docetaxel seen in the phase II trial^23^. Despite this disappointing result, several studies are underway to investigate combination approaches of selumetinib with a variety of partner drugs^24^. In addition, trametinib (GSK1120212) is an oral, reversible, potent and selective inhibitor of MEK1/2 with IC_50_ of 0.7–0.9 nM^25^. FDA recently approved the combination of dabrafenib and trametinib for the treatment of BRAF-mutant metastatic melanoma, NSCLC and anaplastic thyroid cancer^26^. These examples have highlighted the potential of MEK inhibitors as potential targeted anticancer drugs. The limitations of present inhibitors on efficacy and/or adverse effects also put forward an urgent need for the discovery of novel MEK inhibitors.

**Figure 2.**
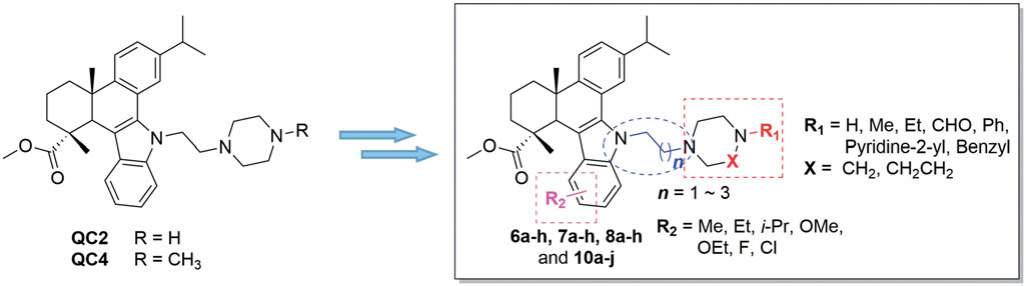
The strategy for the structure modification of QC2 and QC4.

Dehydroabietic acid (DAA) is a natural occurring diterpenic resin acid, which can be easily obtained from *Pinus* rosin or commercial disproportionated rosin. Recent reports indicate that DAA and its derivatives exhibited a broad spectrum of biological activities, such as antimicrobial, antitumour, antiviral, antiprotozoal, antiulcer, antioxidant, anti-ageing and BK-channel opening activities^27–34^. Therefore, DAA has proved to be a promising starting material in search of derivatives with potent anticancer activities. In our previous studies, a series of *N*-substituted 1*H*-dibenzo[*a*,*c*]carbazole derivatives of DAA were synthesised, some of which showed notable antimicrobial activities^35^. Subsequently, in the *in vitro* cytotoxic assay, two compounds (QC2 and QC4) (Figure 2) of these derivatives exhibited significant antiproliferative activity against hepatocarcinoma and gastric cancer cell lines with IC_50_ values at low micromolar level. In pharmacological studies, it was found that QC2 could activate oncosis related protein calpain to induce the damage of cytomembrane and organelles which finally lead to oncosis in hepatocarcinoma cells^36^. QC4 could also induce the oncosis and apoptosis in gastric cancer cells^37^. In addition, QC2 showed moderate inhibitory activity in a preliminary screening of *in vitro* MEK1 inhibitory activity. Based on these findings, the two compounds were subject to further structure modifications at the following sites: (i) the *N*-substituents on the piperazine moiety of the side chain; (ii) the length of the alkyl linker and (iii) the substituents on the indole benzene ring (Figure 2). Through these strategies, several series of compounds derived from QC2 and QC4 can be designed and synthesised in order to find derivatives with better anticancer activities. Furthermore, the anticancer mechanisms of the active compounds will also be extensively explored.

## 2. Experimental

### 2.1. General

IR spectra were measured on a Nexus 870 FTIR spectrometer (Thermo Fisher Scientific, Waltham, MA, USA), and the absorption bands are expressed in cm^−1^. The HRMS spectra were recorded on a high-resolution mass spectrometer equipped with electrospray (ESI) and nanospray sources, and a quadrupole-time of flight hybrid analyzer (Q-TOF Premier/nanoAquity, Waters, Milford, MA). ^1^H NMR and 13 C NMR spectra were accomplished in CDCl_3_ on a Bruker AV-300, AV-500 and DRX-600 NMR spectrometer (Billerica, MA, USA) using TMS as internal standard. Reactions and the resulted products were monitored by TLC which was carried out on TLC Silica gel 60 F254 Aluminium sheets from Merck KGaA, Darmstadt, Germany and visualised in UV light (254 nm). Silica gel (3 0 0–400 mesh) for column chromatography was purchased from Qingdao Marine Chemical Factory, China. The reagents (chemicals), all being of A.R. grade, were purchased from Shanghai Chemical Reagent Company (Shanghai, China) and Energy Chemical (Shanghai, China). Disproportionated rosin was provided by Zhongbang Chemicals Co., Ltd. (Zhaoqing, China), from which dehydroabietic acid (97%) was isolated according to the published method^38^.

### 2.2. General procedure for the synthesis of compounds 5a-c

To a solution of compound **4** (0.7 g, 1.75 mmol) in benzene (5 mL) were added one kind of dibromoalkane (20 mmol), tetrabutyl ammonium bromide (TBAB) (0.02 g, 0.062 mmol) and 50% NaOH solution (3 mL). The mixture was stirred at room temperature for 12 h. Then the mixture was poured into 100 mL of ice-cold water. The suspension was extracted with CH_2_Cl_2_ (3 × 80 mL). The organic layer was combined, washed with water and brine, dried over anhydrous Na_2_SO_4_ and concentrated *in vacuo*. The residue was purified by column chromatography on a silica gel column, eluting with petroleum ether-acetone (100:1, v/v) to give compounds **5a-c**.

#### 2.2.1. 2,3,4,4a,9,13c-Hexahydro-7-isopropyl-1,4a-dimethyl-9–(2-bromoethyl)-1H- dibenzo[a,c]carbazole-1-carboxylic acid methyl ester (5a)

Yield 56%; light yellow resin; ^1^H NMR (300 MHz, CDCl_3_): 1.05 (s, 3H), 1.32 (d, *J* = 7.0 Hz, 3H), 1.35 (d, *J* = 7.0 Hz, 3H), 1.65 (m, 1H), 1.73 (s, 3H), 1.8 0 ∼ 1.99 (m, 4H), 2.29 (d, *J* = 13.4 Hz, 1H), 2.98 (m, 1H), 3.59 (m, 1H), 3.62 (s, 3H), 3.69 (m, 1H), 3.72 (s, 1H), 4.80 (m, 2H), 7.07 (t, *J* = 7.8 Hz, 1H), 7.17 (d, *J* = 7.9 Hz, 1H), 7.19 (t, *J* = 7.5 Hz, 1H), 7.30 (d, *J* = 1.2 Hz, 1H), 7.31 (d, *J* = 7.9 Hz, 1H), 7.34 (d, *J* = 8.2 Hz, 1H), 7.39 (d, *J* = 8.2 Hz, 1H); IR (KBr, cm^−1^): 2963, 2932, 2866, 1717, 1460, 1439, 1381, 1341, 1253, 1220, 1138, 833; HRMS (ESI): *m*/*z* [M + H]^+^ calcd. for C_29_H_35_BrNO_2_: 508.1851; found: 508.1858.

#### 2.2.2. 2,3,4,4a,9,13c-Hexahydro-7-isopropyl-1,4a-dimethyl-9–(3-bromopropyl)-1H- dibenzo[a,c]carbazole-1-carboxylic acid methyl ester (5b)

Yield 48%; light yellow resin; ^1^H NMR (300 MHz, CDCl_3_): 1.05 (s, 3H), 1.32 (d, *J* = 7.0 Hz, 3H), 1.34 (d, *J* = 7.0 Hz, 3H), 1.66 (m, 1H), 1.73 (s, 3H), 1.80–2.00 (m, 4H), 2.11 (m, 2H), 2.28 (d, *J* = 13.8 Hz, 1H), 2.98 (m, 1H), 3.52 (m, 2H), 3.62 (s, 3H), 3.71 (s, 1H), 4.58 (m, 2H), 7.05 (t, *J* = 8.0 Hz, 1H), 7.15 (d, *J* = 8.0 Hz, 1H), 7.16 (t, *J* = 7.5 Hz, 1H), 7.28 (d, *J* = 7.8 Hz, 1H), 7.32 (d, *J* = 8.0 Hz, 1H), 7.36 (s, 1H), 7.38 (d, *J* = 8.4 Hz, 1H); IR (KBr, cm^−1^): 2965, 2931, 2869, 1712, 1463, 1435, 1380, 1343, 1248, 1217, 1130, 829; HRMS (ESI): *m*/*z* [M + H]^+^ calcd. for C_30_H_37_BrNO_2_: 522.2008; found: 522.2003.

#### 2.2.3. 2,3,4,4a,9,13c-Hexahydro-7-isopropyl-1,4a-dimethyl-9–(4-bromobutyl)-1H- dibenzo[a,c]carbazole-1-carboxylic acid methyl ester (5c)

Yield 55%; light yellow resin; ^1^H NMR (300 MHz, CDCl_3_): 1.06 (s, 3H), 1.32 (d, *J* = 7.0 Hz, 3H), 1.35 (d, *J* = 7.1 Hz, 3H), 1.65 (m, 1H), 1.72 (s, 3H), 1.75 (m, 2H), 1.80–2.05 (m, 6H), 2.28 (d, *J* = 13.8 Hz, 1H), 2.96 (m, 1H), 3.50 (m, 2H), 3.61 (s, 3H), 3.72 (s, 1H), 4.47 (m, 2H), 7.04 (t, *J* = 7.8 Hz, 1H), 7.15 (d, *J* = 7.7 Hz, 1H), 7.16 (t, *J* = 7.5 Hz, 1H), 7.29 (d, *J* = 8.0 Hz, 1H), 7.33 (d, *J* = 8.2 Hz, 1H), 7.38 (s, 1H), 7.40 (d, *J* = 8.1 Hz, 1H); IR (KBr, cm^−1^): 2968, 2932, 2874, 1716, 1461, 1432, 1382, 1351, 1236, 1227, 1118, 837; HRMS (ESI): *m*/*z* [M + H]^+^ calcd. for C_31_H_39_BrNO_2_: 536.2164; found: 536.2170.

### 2.3. General procedure for the synthesis of compounds 6a-h, 7a-h and 8a-h

To a solution of compound **5a-c** (0.5 mmol) in acetonitrile (15 mL) was added anhydrous K_2_CO_3_ (0.345 g, 2.5 mmol), KI (0.083 g, 0.5 mmol) and 10 mmol of corresponding *N*-substituted piperazine. The mixture was refluxed for 8–12 h and monitored by TLC. At the end of reaction, the mixture was poured into cold water, which was extracted by CH_2_Cl_2_ (100 mL) for three times. The organic phase was combined, washed with water and brine, dried over anhydrous Na_2_SO_4_ and concentrated *in vacuo*. The residue was subjected to silica gel chromatography, eluting with petroleum ether-acetone (100:1, v/v) to afford compounds **6a-h**, **7a-h** or **8a-h**.

#### 2.3.1. 2,3,4,4a,9,13c-Hexahydro-7-isopropyl-1,4a-dimethyl-9–(2-(piperazin-1-yl)ethyl) -1H-dibenzo[a,c]carbazole-1-carboxylic acid methyl ester (6a)

Yellow amorphous solid; Yield: 60%; ^1^H NMR (300 MHz, CDCl_3_) *δ*: 1.06 (s, 3H), 1.32 (d, *J* = 6.9 Hz, 3H), 1.33 (d, *J* = 6.9 Hz, 3H), 1.66 (m, 1H), 1.74 (s, 3H), 1.79–2.01 (m, 4H), 2.30 (d, *J* = 12.7 Hz, 1H), 2.40 (brs, 1H, NH), 2.54 (t, *J* = 4.4 Hz, 4H), 2.80 (m, 2H), 2.89 (t, *J* = 4.4 Hz, 4H), 2.99 (m, 1H), 3.62 (s, 3H), 3.72 (s, 1H), 4.60 (m, 2H), 7.05 (t, *J* = 8.1 Hz, 1H), 7.15 (dd, *J* = 7.5, 1.3 Hz, 1H), 7.18 (t, *J* = 7.9 Hz, 1H), 7.30 (d, *J* = 8.2 Hz, 1H), 7.34 (d, *J* = 8.4 Hz, 1H), 7.43 (d, *J* = 8.1 Hz, 1H), 7.47 (d, *J* = 1.7 Hz, 1H); ^13^C NMR (150 MHz, CDCl_3_) *δ*: 18.3, 19.3, 21.2, 24.2, 24.3, 34.1, 36.6, 38.7, 38.8, 43.3, 45.6, 45.7, 46.1, 52.5, 54.3, 58.1, 110.1, 113.9, 119.8, 120.9, 121.1, 121.6, 123.5, 125.4, 126.0, 127.3, 135.6, 139.1, 146.3, 147.1, 180.4; IR (KBr, cm^−1^): 3042, 2928, 2860, 2811, 1723, 1607, 1495, 1461, 1350, 1255, 1134, 826, 737; HRMS (ESI): *m*/*z* [M + H]^+^ calcd. for C_33_H_44_N_3_O_2_: 514.3434; found: 514.3439.

#### 2.3.2. 2,3,4,4a,9,13c-Hexahydro-7-isopropyl-1,4a-dimethyl-9–(2-(4-methylpiperazin-1-yl)ethyl)-1H-dibenzo[a,c]carbazole-1-carboxylic acid methyl ester (6b)

Yellow amorphous solid; Yield: 60%; ^1^H NMR (300 MHz, CDCl_3_) *δ*: 1.05 (s, 3H), 1.31 (d, *J* = 6.9 Hz, 3H), 1.33 (d, *J* = 6.9 Hz, 3H), 1.65 (m, 1H), 1.74 (s, 3H), 1.792.01 (m, 4H), 2.30 (d, *J* = 12.8 Hz, 1H), 2.31 (s, 3H), 2.47 (brs, 4H), 2.61 (brs, 4H), 2.81 (m, 1H), 2.90–3.01 (m, 2H), 3.61 (s, 3H), 3.72 (s, 1H), 4.57 (m, 2H), 7.05 (t, *J* = 8.0 Hz, 1H), 7.15 (d, *J* = 8.1, 1H), 7.16 (t, *J* = 8.1 Hz, 1H), 7.30 (d, *J* = 8.5 Hz, 1H), 7.33 (d, *J* = 8.4 Hz, 1H), 7.43 (d, *J* = 8.2 Hz, 1H), 7.47 (s, 1H); ^13^C NMR (150 MHz, CDCl_3_) *δ*: 18.3, 19.3, 21.3, 24.2, 24.3, 34.1, 36.6, 38.7, 38.8, 43.5, 45.7, 46.1, 46.1, 52.5, 53.7, 55.1, 57.5, 110.1, 113.8, 119.8, 120.9, 121.0, 121.7, 123.4, 125.3, 125.9, 127.2, 135.6, 139.2, 146.4, 147.1, 180.4; IR (KBr, cm^−1^): 3046, 2929, 2862, 2797, 1724, 1604, 1460, 1354, 1252, 1166, 826, 736; HRMS (ESI): *m*/*z* [M + H]^+^ calcd. for C_34_H_46_N_3_O_2_ 528.3590; found: 528.3587.

#### 2.3.3. 2,3,4,4a,9,13c-Hexahydro-7-isopropyl-1,4a-dimethyl-9–(2-(4-ethylpiperazin -1-yl)ethyl)-1H-dibenzo[a,c]carbazole-1-carboxylic acid methyl ester (6c)

Yellow amorphous solid; Yield: 50%; ^1^H NMR (300 MHz, CDCl_3_) *δ*: 1.05 (s, 3H), 1.10 (t, *J* = 7.0 Hz, 3H), 1.31 (d, *J* = 6.6 Hz, 3H), 1.33 (d, *J* = 6.5 Hz, 3H), 1.66 (m, 1H), 1.74 (s, 3H), 1.75–2.10 (m, 4H), 2.29 (d, *J* = 11.1 Hz, 1H), 2.44 (q, *J* = 7.1 Hz, 2H), 2.50 (brs, 4H), 2.63 (brs, 4H), 2.84 (m, 1H), 2.94 (m, 2H), 3.61 (s, 3H), 3.72 (s, 1H), 4.45 (m, 2H), 7.05 (t, *J* = 7.6 Hz, 1H), 7.15 (d, *J* = 7.8 Hz, 1H), 7.17 (t, *J* = 7.8 Hz, 1H), 7.31 (d, *J* = 9.3 Hz, 1H), 7.34 (d, *J* = 8.6 Hz, 1H), 7.44 (d, *J* = 8.1 Hz, 1H), 7.48 (s, 1H); ^13^C NMR (150 MHz, CDCl_3_) *δ*: 11.8, 18.2, 19.2, 21.2, 24.1, 24.3, 34.0, 36.5, 38.6, 38.7, 43.4, 45.6, 46.0, 52.3, 52.4, 52.6, 53.5, 57.5, 110.1, 113.7, 119.7, 120.8, 121.0, 121.6, 123.4, 125.2, 125.9, 127.2, 135.5, 139.1, 146.3, 147.0, 180.3; IR (KBr, cm^−1^): 3029, 2924, 2856, 2806, 1716, 1603, 1453, 1336, 1160, 1128, 730; HRMS (ESI): *m*/*z* [M + H]^+^ calcd. for C_35_H_48_N_3_O_2_: 542.3747; found: 542.3753.

#### 2.3.4. 2,3,4,4a,9,13c-Hexahydro-7-isopropyl-1,4a-dimethyl-9–(2-(1,4-diazepan-1-yl) ethyl)-1H-dibenzo[a,c]carbazole-1-carboxylic acid methyl ester (6d)

Yellow amorphous solid; Yield: 32%; ^1^H NMR (500 MHz, CDCl_3_) *δ*: 1.05 (s, 3H), 1.30 (d, *J* = 7.2 Hz, 3H), 1.32 (d, *J* = 6.9 Hz, 3H), 1.65 (m, 1H), 1.74 (s, 3H), 1.80–2.10 (m, 6H), 2.29 (d, *J* = 11.5 Hz, 1H), 2.78–2.82 (m, 5H), 2.85–3.05 (m, 5H), 3.05 (t, *J* = 5.5 Hz, 2H), 3.61 (s, 3H), 3.71 (s, 1H), 4.56 (m, 2H), 7.04 (t, *J* = 7.5 Hz, 1H), 7.15 (d, *J* = 8.2 Hz, 1H), 7.16 (t, *J* = 9.3 Hz, 1H), 7.30 (d, *J* = 8.1 Hz, 1H), 7.33 (d, *J* = 8.3 Hz, 1H), 7.40 (d, *J* = 8.2 Hz, 1H), 7.44 (s, 1H); ^13^C NMR (125 MHz, CDCl_3_) *δ*: 18.3, 19.3, 21.2, 22.8, 24.2, 24.3, 27.4, 34.1, 36.6, 38.8, 38.8, 44.1, 45.7, 46.0, 46.1, 47.8, 52.5, 54.9, 57.4, 110.1, 114.2, 119.9, 121.0, 121.2, 121.4, 123.6, 125.5, 126.1, 127.4, 135.6, 139.4, 146.4, 147.1, 180.4; IR (KBr, cm^−1^): 3380, 3046, 2955, 2928, 2851, 2806, 1729, 1653, 1454, 1359, 1241, 1110, 730; HRMS (ESI): *m*/*z* [M + H]^+^ calcd. for C_34_H_46_N_3_O_2_: 528.3590; found: 528.3582.

#### 2.3.5. 2,3,4,4a,9,13c-Hexahydro-7-isopropyl-1,4a-dimethyl-9–(2-(4-formylpiperazin -1-yl)ethyl)-1H-dibenzo[a,c]carbazole-1-carboxylic acid methyl ester (6e)

Yellow amorphous solid; Yield: 61%; ^1^H NMR (300 MHz, CDCl_3_) *δ*: 1.06 (s, 3H), 1.30 (d, *J* = 6.9 Hz, 3H), 1.32 (d, *J* = 6.9 Hz, 3H), 1.65 (m, 1H), 1.74 (s, 3H), 1.76–2.08 (m, 4H), 2.29 (d, *J* = 12.2 Hz, 1H), 2.47 (brs, 4H), 2.82 (m, 2H), 2.96 (m, 1H), 3.26 (m, 2H), 3.44 (m, 2H), 3.62 (s, 3H), 3.71 (s, 1H), 4.61 (t, *J* = 6.8 Hz, 2H), 7.05 (t, *J* = 7.7 Hz, 1H), 7.15 (d, *J* = 7.5, 1H), 7.16 (t, *J* = 7.4 Hz, 1H), 7.31 (d, *J* = 8.7 Hz, 1H), 7.34 (d, *J* = 9.0 Hz, 1H), 7.40 (d, *J* = 8.1 Hz, 1H), 7.45 (s, 1H), 7.98 (s, 1H, CHO); ^13^C NMR (150 MHz, CDCl_3_) *δ*: 18.2, 19.3, 21.3, 24.2, 24.3, 34.1, 36.6, 38.7, 39.8, 43.2, 45.5, 45.6, 46.0, 52.5, 52.8, 54.1, 57.5, 110.0, 114.1, 119.9, 121.0, 121.1, 121.4, 123.6, 125.5, 126.1, 127.3, 135.7, 139.1, 146.3, 147.1, 160.8, 180.4; IR (KBr, cm^−1^): 3010, 2923, 2851, 1719, 1677, 1460, 1218, 1134, 997, 739; HRMS (ESI): *m*/*z* [M + H]^+^ calcd. for C_34_H_44_N_3_O_3_: 542.3383; found: 542.3389.

#### 2.3.6. 2,3,4,4a,9,13c-Hexahydro-7-isopropyl-1,4a-dimethyl-9–(2-(4-phenylpiperazin-1-yl)ethyl)-1H-dibenzo[a,c]carbazole-1-carboxylic acid methyl ester (6f)

Yellow amorphous solid; Yield: 45%; ^1^H NMR (300 MHz, CDCl_3_) *δ*: 1.05 (s, 3H), 1.32 (d, *J* = 6.9 Hz, 3H), 1.33 (d, *J* = 6.4 Hz, 3H), 1.65 (m, 1H), 1.74 (s, 3H), 1.80–2.10 (m, 4H), 2.28 (d, *J* = 9.3 Hz, 1H), 2.65 (m, 2H), 2.69 (m, 4H), 2.97 (m, 1H), 3.16 (m, 4H), 3.61 (s, 3H), 3.72 (s, 1H), 4.61 (m, 2H), 6.84 (t, *J* = 7.3 Hz, 1H), 6.90 (d, *J* = 8.2 Hz, 2H), 7.05 (t, *J* = 7.8 Hz, 1H), 7.14 (d, *J* = 7.1 Hz, 1H), 7.16 (t, *J* = 7.3 Hz, 1H), 7.24 (t, *J* = 7.8 Hz, 2H), 7.29 (d, *J* = 8.1 Hz, 1H), 7.34 (d, *J* = 8.3 Hz, 1H), 7.45 (d, *J* = 8.2 Hz, 1H), 7.50 (s, 1H); ^13^C NMR (125 MHz, CDCl_3_) *δ*: 18.3, 19.3, 21.3, 24.2, 24.4, 34.1, 36.6, 38.8, 38.8, 43.5, 45.7, 46.1, 49.1, 52.5, 53.8, 57.6, 110.2, 113.9, 116.2, 119.8, 119.9, 120.9, 121.1, 121.6, 123.5, 125.4, 126.0, 127.3, 129.2, 135.7, 139.2, 146.4, 147.1, 151.4, 180.4; IR (KBr, cm^−1^): 3011, 2953, 2924, 2851, 1724, 1600, 1497, 1463, 1383, 1233, 1139, 730; HRMS (ESI): *m*/*z* [M + H]^+^ calcd. for C_39_H_48_N_3_O_2_: 590.3747; found: 590.3753.

#### 2.3.7. 2,3,4,4a,9,13c-Hexahydro-7-isopropyl-1,4a-dimethyl-9–(2-(4-(pyridine-2-yl) piperazin-1-yl)ethyl)-1H-dibenzo[a,c]carbazole-1-carboxylic acid methyl ester (6g)

Yellow amorphous solid; Yield: 50%; ^1^H NMR (500 MHz, CDCl_3_) *δ*: 1.05 (s, 3H), 1.32 (d, *J* = 6.7 Hz, 3H), 1.33 (d, *J* = 6.7 Hz, 3H), 1.65 (m, 1H), 1.74 (s, 3H), 1.8 0–2.10 (m, 4H), 2.28 (d, *J* = 12.3 Hz, 1H), 2.64 (t, *J* = 5.0 Hz, 4H), 2.84 (m, 1H), 2.90–3.00 (m, 2H), 3.50 (m, 4H), 3.61 (s, 3H), 3.72 (s, 1H), 4.63 (m, 2H), 6.60 (t, *J* = 4.0 Hz, 1H), 6.62 (s, 1H), 7.05 (t, *J* = 7.6 Hz, 1H), 7.14 (d, *J* = 7.3 Hz, 1H), 7.16 (t, *J* = 7.4 Hz, 1H), 7.29 (d, *J* = 8.1 Hz, 1H), 7.34 (d, *J* = 8.3 Hz, 1H), 7.45 (d, *J* = 8.2 Hz, 1H), 7.46 (m, 1H), 7.50 (s, 1H), 8.18 (d, *J* = 3.8 Hz, 1H); ^13^C NMR (125 MHz, CDCl_3_) *δ*: 18.3, 19.3, 21.3, 24.2, 24.4, 34.1, 36.6, 38.8, 38.8, 43.5, 45.3, 45.7, 46.1, 52.5, 53.6, 57.7, 107.2, 110.2, 113.5, 114.0, 119.8, 120.9, 121.1, 121.6, 123.5, 125.4, 126.0, 127.3, 135.7, 137.6, 139.2, 146.4, 147.1, 148.1, 159.6, 180.4; IR (KBr, cm^−1^): 3010, 2924, 2851, 1723, 1593, 1480, 1382, 1243, 1126, 732; HRMS (ESI): *m*/*z* [M + H]^+^ calcd. for C_38_H_47_N_4_O_2_: 591.3699; found: 591.3706.

#### 2.3.8. 2,3,4,4a,9,13c-Hexahydro-7-isopropyl-1,4a-dimethyl-9–(2-(4-benzylpiperazin -1-yl)ethyl)-1H-dibenzo[a,c]carbazole-1-carboxylic acid methyl ester (6h)

Yellow amorphous solid; Yield: 64%; ^1^H NMR (300 MHz, CDCl_3_) *δ*: 1.04 (s, 3H), 1.30 (d, *J* = 6.4 Hz, 3H), 1.32 (d, *J* = 6.2 Hz, 3H), 1.64 (m, 1H), 1.73 (s, 3H), 1.76–2.10 (m, 4H), 2.28 (d, *J* = 11.8 Hz, 1H), 2.48 (brs, 4H), 2.60 (brs, 4H), 2.90 (m, 2H), 2.96 (m, 1H), 3.52 (s, 2H), 3.61 (s, 3H), 3.71 (s, 1H), 4.56 (m, 2H), 7.04 (t, *J* = 7.6 Hz, 1H), 7.14 (d, *J* = 7.7, 1H), 7.16 (t, *J* = 7.0 Hz, 1H), 7.29 (d, *J* = 7.5 Hz, 1H), 7.31 (s, 5H), 7.32 (d, *J* = 10.2 Hz, 1H), 7.42 (d, *J* = 8.2 Hz, 1H), 7.47 (s, 1H); ^13^C NMR (150 MHz, CDCl_3_) *δ*: 18.2, 19.3, 21.2, 24.1, 24.3, 34.0, 36.5, 38.6, 38.7, 43.5, 45.6, 46.0, 52.4, 52.9, 53.7, 57.5, 63.1, 110.1, 113.7, 119.7, 120.8, 121.0, 121.6, 123.4, 125.3, 125.9, 127.2, 127.2, 128.3, 129.3, 135.5, 138.0, 139.1, 146.3, 147.0, 180.4; IR (KBr, cm^−1^): 3028, 2924, 2851, 2809, 1722, 1457, 1350, 1251, 1140, 734, 697; HRMS (ESI): *m*/*z* [M + H]^+^ calcd. for C_40_H_50_N_3_O_2_: 604.3903; found: 604.3898.

#### 2.3.9. 2,3,4,4a,9,13c-Hexahydro-7-isopropyl-1,4a-dimethyl-9–(2-(piperazin-1-yl) propyl)-1H-dibenzo[a,c]carbazole-1-carboxylic acid methyl ester (7a)

Yellow amorphous solid; Yield: 67%; ^1^H NMR (300 MHz, CDCl_3_) *δ*: 1.04 (s, 3H), 1.30 (d, *J* = 6.8 Hz, 3H), 1.32 (d, *J* = 6.7 Hz, 3H), 1.64 (m, 1H), 1.73 (s, 3H), 1.75–2.15 (m, 5H), 2.1 8 ∼ 2.40 (m, 4H), 2.43 (brs, 1H, NH), 2.50 (m, 4H), 2.95 (m, 1H), 3.02 (m, 4H), 3.61 (s, 3H), 3.70 (s, 1H), 4.51 (m, 2H), 7.03 (t, *J* = 7.5 Hz, 1H), 7.13 (d, *J* = 7.7 Hz, 1H), 7.15 (t, *J* = 7.7 Hz, 1H), 7.29 (d, *J* = 8.6 Hz, 1H), 7.32 (d, *J* = 8.7 Hz, 1H), 7.39 (d, *J* = 9.4 Hz, 1H), 7.40 (s, 1H); ^13^C NMR (150 MHz, CDCl_3_) *δ*: 18.0, 19.1, 20.9, 24.0, 24.1, 27.2, 33.7, 36.3, 38.4, 38.5, 43.5, 45.4, 45.9, 49.5, 52.3, 54.5, 55.3, 110.2, 113.7, 119.5, 120.6, 120.8, 121.1, 123.3, 125.2, 125.7, 127.1, 135.2, 139.1, 146.0, 146.7, 180.1; IR (KBr, cm^−1^): 3486, 3041, 2946, 2929, 2858, 1722, 1608, 1462, 1361, 1249, 1137, 732; HRMS (ESI): *m*/*z* [M + H]^+^ calcd. for C_34_H_46_N_3_O_2_: 528.3590; found: 528.3593.

#### 2.3.10. 2,3,4,4a,9,13c-Hexahydro-7-isopropyl-1,4a-dimethyl-9–(3-(4-methyl piperazin-1-yl)propyl)-1H-dibenzo[a,c]carbazole-1-carboxylic acid methyl ester (7b)

Yellow amorphous solid; Yield: 49%; ^1^H NMR (300 MHz, CDCl_3_): *δ* 1.04 (s, 3H), 1.31 (d, *J* = 6.3 Hz, 3H), 1.32 (d, *J* = 6.8 Hz, 3H), 1.65 (m, 1H), 1.73 (s, 3H), 1.75–2.15 (m, 6H), 2.28 (d, *J* = 12.1 Hz, 1H), 2.30 (s, 3H), 2.36 (t, *J* = 7.2 Hz, 2H), 2.45 (brs, 8H), 2.95 (m, 1H), 3.61 (s, 3H), 3.71 (s, 1H), 4.49 (t, *J* = 7.4 Hz, 2H), 7.03 (t, *J* = 7.6 Hz, 1H), 7.14 (d, *J* = 7.8 Hz, 1H), 7.15 (t, *J* = 7.8 Hz, 1H), 7.29 (d, *J* = 8.6 Hz, 1H), 7.32 (d, *J* = 8.5 Hz, 1H), 7.41 (s, 1H), 7.42 (d, *J* = 9.7 Hz, 1H); ^13^C NMR (150 MHz, CDCl_3_) *δ*: 18.0, 19.1, 21.0, 24.0, 24.1, 27.4, 33.7, 36.3, 38.4, 38.5, 43.3, 45.4, 45.9, 52.2, 53.0, 55.0, 55.4, 110.2, 113.7, 119.4, 120.5, 120.7, 121.2, 123.2, 125.2, 125.6, 127.2, 135.2, 139.0, 146.0, 146.7, 180.1; IR (KBr, cm^−1^): 3045, 2933, 2871, 2794, 1722, 1609, 1460, 1359, 1283, 1164, 734; HRMS (ESI): *m*/*z* [M + H]^+^ calcd. for C_35_H_48_N_3_O_2_: 542.3747; found: 542.3741.

#### 2.3.11. 2,3,4,4a,9,13c-Hexahydro-7-isopropyl-1,4a-dimethyl-9–(2-(4-ethylpiperazin -1-yl)propyl)-1H-dibenzo[a,c]carbazole-1-carboxylic acid methyl ester (7c)

Yellow amorphous solid; Yield: 38%; ^1^H NMR (300 MHz, CDCl_3_) *δ*: 1.06 (s, 3H), 1.09 (t, *J* = 7.2 Hz, 3H), 1.31 (d, *J* = 6.2 Hz, 3H), 1.33 (d, *J* = 6.5 Hz, 3H), 1.65 (m, 1H), 1.75 (s, 3H), 1.80–2.10 (m, 6H), 2.28 (d, *J* = 12.1 Hz, 1H), 2.36 (t, *J* = 6.5 Hz, 2H), 2.42 (q, *J* = 7.2 Hz, 2H), 2.47 (brs, 8H), 2.96 (m, 1H), 3.61 (s, 3H), 3.72 (s, 1H), 4.50 (t, *J* = 7.6 Hz, 2H), 7.04 (t, *J* = 7.5 Hz, 1H), 7.14 (d, *J* = 7.8 Hz, 1H), 7.15 (t, *J* = 8.3 Hz, 1H), 7.29 (d, *J* = 8.1 Hz, 1H), 7.34 (d, *J* = 8.3 Hz, 1H), 7.42 (s, 1H), 7.43 (d, *J* = 8.5 Hz, 1H); ^13^C NMR (125 MHz, CDCl_3_) *δ*: 11.9, 18.2, 19.2, 21.1, 24.1, 24.2, 27.6, 33.9, 36.5, 38.6, 38.7, 43.5, 45.6, 46.1, 52.3, 52.4, 52.8, 53.2, 55.6, 110.3, 113.9, 119.6, 120.7, 120.9, 121.4, 123.3, 125.3, 125.8, 127.4, 135.4, 139.2, 146.2, 147.0, 180.3; IR (KBr, cm^−1^): 3049, 2962, 2924, 2852, 1725, 1604, 1463, 1380, 1336, 1248, 1190, 908; HRMS (ESI): *m*/*z* [M + H]^+^ calcd. for C_36_H_50_N_3_O_2_: 556.3903; found: 556.3909.

#### 2.3.12. 2,3,4,4a,9,13c-Hexahydro-7-isopropyl-1,4a-dimethyl-9–(2-(1,4-diazepan-1-yl) propyl)-1H-dibenzo[a,c]carbazole-1-carboxylic acid methyl ester (7d)

Yellow amorphous solid; Yield: 41%; ^1^H NMR (300 MHz, CDCl_3_) *δ*: 1.05 (s, 3H), 1.31 (d, *J* = 6.6 Hz, 3H), 1.32 (d, *J* = 6.5 Hz, 3H), 1.65 (m, 1H), 1.74 (s, 3H), 1.76–2.10 (m, 8H), 2.29 (d, *J* = 10.6 Hz, 1H), 2.3 5 ∼ 2.55 (m, 5H), 2.63 (m, 4H), 2.95 (m, 1H), 2.99 (m, 2H), 3.61 (s, 3H), 3.71 (s, 1H), 4.52 (t, *J* = 7.1 Hz, 2H), 7.03 (t, *J* = 7.7 Hz, 1H), 7.14 (d, *J* = 7.8 Hz, 1H), 7.15 (t, *J* = 7.8 Hz, 1H), 7.30 (d, *J* = 8.4 Hz, 1H), 7.32 (d, *J* = 8.4 Hz, 1H), 7.42 (s, 1H), 7.43 (d, *J* = 7.0 Hz, 1H); ^13^C NMR (150 MHz, CDCl_3_) *δ*: 18.2, 19.2, 21.1, 24.2, 24.2, 25.2, 26.4, 28.1, 33.9, 36.5, 38.6, 38.7, 43.0, 45.6, 46.0, 48.0, 51.4, 52.5, 54.2, 55.0, 110.4, 114.1, 119.7, 121.0, 121.2, 121.3, 123.4, 125.5, 125.7, 127.3, 135.3, 139.2, 146.2, 146.9, 180.4; IR (KBr, cm^−1^): 3421, 3037, 2949, 2928, 2861, 1720, 1609, 1462, 1382, 1359, 1248, 1110, 733; HRMS (ESI): *m*/*z* [M + H]^+^ calcd. for C_35_H_48_N_3_O_2_: 542.3747; found: 542.3742.

#### 2.3.13. 2,3,4,4a,9,13c-Hexahydro-7-isopropyl-1,4a-dimethyl-9–(2-(4-formylpiperazin-1-yl)propyl)-1H-dibenzo[a,c]carbazole-1-carboxylic acid methyl ester (7e)

Yellow amorphous solid; Yield: 52%; ^1^H NMR (300 MHz, CDCl_3_) *δ*: 1.05 (s, 3H), 1.31 (d, *J* = 6.8 Hz, 3H), 1.32 (d, *J* = 6.8 Hz, 3H), 1.65 (m, 1H), 1.74 (s, 3H), 1.75–2.12 (m, 6H), 2.2 0 ∼ 2.40 (m, 7H), 2.96 (m, 1H), 3.32 (brs, 2H), 3.50 (brs, 2H), 3.62 (s, 3H), 3.71 (s, 1H), 4.56 (m, 2H), 7.04 (t, *J* = 7.8 Hz, 1H), 7.15 (d, *J* = 7.9 Hz, 1H), 7.16 (t, *J* = 7.9 Hz, 1H), 7.30 (d, *J* = 8.6 Hz, 1H), 7.33 (d, *J* = 8.8 Hz, 1H), 7.41 (s, 1H), 7.42 (d, *J* = 7.4 Hz, 1H), 7.99 (s, 1H, CHO); ^13^C NMR (125 MHz, CDCl_3_) *δ*: 18.2, 19.2, 21.1, 24.1, 24.2, 27.3, 33.9, 36.5, 38.6, 38.7, 39.9, 43.1, 45.6, 46.0, 52.4, 52.5, 53.6, 55.3, 110.3, 114.1, 119.7, 120.8, 120.9, 121.3, 123.4, 125.4, 125.9, 127.3, 135.4, 139.2, 146.1, 146.9, 160.7, 180.3; IR (KBr, cm^−1^): 3041, 2958, 2930, 2861, 2808, 2773, 1722, 1679, 1608, 1436, 1398, 1260, 1108, 733; HRMS (ESI): *m*/*z* [M + H]^+^ calcd. for C_35_H_46_N_3_O_3_: 556.3539; found: 556.3534.

#### 2.3.14. 2,3,4,4a,9,13c-Hexahydro-7-isopropyl-1,4a-dimethyl-9–(2-(4-phenyl piperazin-1-yl)propyl)-1H-dibenzo[a,c]carbazole-1-carboxylic acid methyl ester (7f)

Yellow amorphous solid; Yield: 46%; ^1^H NMR (300 MHz, CDCl_3_) *δ*: 1.06 (s, 3H), 1.30 (d, *J* = 7.7 Hz, 3H), 1.31 (d, *J* = 6.8 Hz, 3H), 1.64 (m, 1H), 1.74 (s, 3H), 1.80–2.10 (m, 6H), 2.27 (d, *J* = 10.8 Hz, 1H), 2.35 (brs, 2H), 2.51 (m, 4H), 2.94 (m, 1H), 3.15 (brs, 4H), 3.58 (s, 3H), 3.72 (s, 1H), 4.53 (brs, 2H), 6.81 (t, *J* = 6.8 Hz, 1H), 6.87 (d, *J* = 7.6 Hz, 2H), 7.03 (t, *J* = 7.2 Hz, 1H), 7.12 (d, *J* = 6.1 Hz, 1H), 7.13 (t, *J* = 7.6 Hz, 1H), 7.21 (t, *J* = 7.0 Hz, 2H), 7.28 (d, *J* = 7.7 Hz, 1H), 7.34 (d, *J* = 8.0 Hz, 1H), 7.42 (s, 1H), 7.43 (d, *J* = 7.4 Hz, 1H); ^13^C NMR (125 MHz, CDCl_3_) *δ*: 18.2, 19.3, 21.1, 24.2, 24.3, 27.5, 33.9, 36.6, 38.6, 38.8, 43.4, 45.6, 46.1, 49.1, 52.4, 53.3, 55.5, 110.3, 114.0, 116.1, 119.7, 119.8, 120.8, 120.9, 121.4, 123.4, 125.4, 125.9, 127.4, 129.1, 135.4, 139.3, 146.2, 147.0, 151.3, 180.3; IR (KBr, cm^−1^): 3041, 2949, 2920, 2850, 1721, 1600, 1497, 1462, 1382, 1231, 1139, 733; HRMS (ESI): *m*/*z* [M + H]^+^ calcd. for C_40_H_50_N_3_O_2_: 604.3903; found: 604.3910.

#### 2.3.15. 2,3,4,4a,9,13c-Hexahydro-7-isopropyl-1,4a-dimethyl-9–(2-(4-(pyridine-2-yl) piperazin-1-yl)propyl)-1H-dibenzo[a,c]carbazole-1-carboxylic acid methyl ester (7g)

Yellow amorphous solid; Yield: 52%; ^1^H NMR (300 MHz, CDCl_3_) *δ*: 1.05 (s, 3H), 1.31 (d, *J* = 6.7 Hz, 3H), 1.32 (d, *J* = 6.7 Hz, 3H), 1.66 (m, 1H), 1.74 (s, 3H), 1.78–2.20 (m, 6H), 2.29 (d, *J* = 11.5 Hz, 1H), 2.40 (brs, 2H), 2.49 (brs, 4H), 2.97 (m, 1H), 3.55 (brs, 4H), 3.61 (s, 3H), 3.72 (s, 1H), 4.58 (brs, 2H), 6.62 (d, *J* = 8.0 Hz, 1H), 6.63 (t, *J* = 8.0 Hz, 1H), 7.04 (t, *J* = 7.7 Hz, 1H), 7.15 (d, *J* = 7.7 Hz, 1H), 7.16 (t, *J* = 7.4 Hz, 1H), 7.30 (d, *J* = 8.6 Hz, 1H), 7.33 (d, *J* = 8.9 Hz, 1H), 7.4 0 ∼ 7.50 (m, 3H), 8.18 (d, *J* = 3.5 Hz, 1H); ^13^C NMR (125 MHz, CDCl_3_) *δ*: 18.2, 19.3, 21.2, 24.2, 24.3, 27.5, 34.0, 36.6, 38.7, 38.8, 43.4, 45.3, 45.7, 46.1, 52.5, 53.1, 55.6, 107.2, 110.3, 113.5, 114.0, 119.7, 120.8, 121.0, 121.4, 123.4, 125.4, 125.9, 127.4, 135.5, 137.6, 139.2, 146.2, 147.0, 148.1, 159.6, 180.4; IR (KBr, cm^−1^): 3050, 2954, 2866, 2808, 1718, 1595, 1480, 1432, 1379, 1265, 1115, 733; HRMS (ESI): *m*/*z* [M + H]^+^ calcd. for C_39_H_49_N_4_O_2_: 605.3856; found: 605.3852.

#### 2.3.16. 2,3,4,4a,9,13c-Hexahydro-7-isopropyl-1,4a-dimethyl-9–(2-(4-benzylpiperazin-1-yl)propyl)-1H-dibenzo[a,c]carbazole-1-carboxylic acid methyl ester (7h)

Yellow amorphous solid; Yield: 47%; ^1^H NMR (300 MHz, CDCl_3_) *δ*: 1.04 (s, 3H), 1.30 (d, *J* = 6.8 Hz, 3H), 1.31 (d, *J* = 6.8 Hz, 3H), 1.65 (m, 1H), 1.74 (s, 3H), 1.7 6 ∼ 2.15 (m, 6H), 2.28 (d, *J* = 12.1 Hz, 1H), 2.36 (t, *J* = 7.0 Hz, 2H), 2.46 (brs, 8H), 2.95 (m, 1H), 3.50 (s, 2H), 3.61 (s, 3H), 3.71 (s, 1H), 4.48 (t, *J* = 7.5 Hz, 2H), 7.03 (t, *J* = 7.6 Hz, 1H), 7.13 (d, *J* = 8.0 Hz, 1H), 7.14 (t, *J* = 8.0 Hz, 1H), 7.29 (d, *J* = 8.8 Hz, 1H), 7.30 (s, 5H), 7.33 (d, *J* = 9.5 Hz, 1H), 7.40 (s, 1H), 7.42 (d, *J* = 10.0 Hz, 1H); ^13^C NMR (125 MHz, CDCl_3_) *δ*: 18.1, 19.2, 21.1, 24.1, 24.2, 27.5, 33.9, 36.5, 38.5, 38.7, 43.5, 45.5, 46.0, 52.4, 53.1, 53.2, 55.6, 63.1, 110.3, 113.7, 119.6, 120.6, 120.8, 121.3, 123.3, 125.3, 125.7, 127.0, 127.3, 128.2, 129.2, 135.3, 138.0, 139.1, 146.1, 146.9, 180.3; IR (KBr, cm^−1^): 3028, 2954, 2929, 2870, 2809, 1721, 1607, 1458, 1383, 1345, 1248, 1138, 1012, 734; HRMS (ESI): *m*/*z* [M + H]^+^ calcd. for C_41_H_52_N_3_O_2_: 618.4060; found: 618.4066.

#### 2.3.17. 2,3,4,4a,9,13c-Hexahydro-7-isopropyl-1,4a-dimethyl-9–(2-(piperazin-1-yl) butyl)-1H-dibenzo[a,c]carbazole-1-carboxylic acid methyl ester (8a)

Yellow amorphous solid; Yield: 62%; ^1^H NMR (300 MHz, CDCl_3_) *δ*: 1.04 (s, 3H), 1.30 (d, *J* = 6.8 Hz, 3H), 1.31 (d, *J* = 6.8 Hz, 3H), 1.64 (m, 1H), 1.73 (s, 3H), 1.7 5 ∼ 2.10 (m, 6H), 2.28 (d, *J* = 10.6 Hz, 1H), 2.38 (m, 2H), 2.42–2.75 (m, 7H), 2.95 (m, 1H), 3.10 (m, 4H), 3.61 (s, 3H), 3.71 (s, 1H), 4.45 (m, 2H), 7.04 (t, *J* = 7.6 Hz, 1H), 7.15 (d, *J* = 7.8 Hz, 1H), 7.16 (t, *J* = 7.3 Hz, 1H), 7.30 (d, *J* = 8.0 Hz, 1H), 7.33 (d, *J* = 8.1 Hz, 1H), 7.37 (s, 1H), 7.40 (d, *J* = 9.1 Hz, 1H); ^13^C NMR (150 MHz, CDCl_3_) *δ*: 18.1, 19.1, 21.0, 23.8, 24.1, 24.2, 27.5, 33.8, 36.4, 38.5, 38.6, 43.5, 45.0, 45.5, 46.0, 49.5, 52.4, 57.2, 110.1, 113.7, 119.6, 120.7, 120.9, 121.2, 123.4, 125.4, 125.7, 127.2, 135.4, 138.9, 146.1, 146.9, 180.3; IR (KBr, cm^−1^): 3396, 3046, 2956, 2928, 2867, 2813, 1722, 1609, 1460, 1382, 1360, 1250, 1133, 734; HRMS (ESI): *m*/*z* [M + H]^+^ calcd. for C_35_H_48_N_3_O_2_: 542.3747; found: 542.3741.

#### 2.3.18. 2,3,4,4a,9,13c-Hexahydro-7-isopropyl-1,4a-dimethyl-9–(3-(4-methyl piperazin-1-yl)butyl)-1H-dibenzo[a,c]carbazole-1-carboxylic acid methyl ester (8b)

Yellow amorphous solid; Yield: 63%; ^1^H NMR (300 MHz, CDCl_3_): *δ* 1.04 (s, 3H), 1.30 (d, *J* = 6.3 Hz, 3H), 1.32 (d, *J* = 6.3 Hz, 3H), 1.5 0–1.70 (m, 3H), 1.74 (s, 3H), 1.80–2.10 (m, 6H), 2.29 (d, *J* = 11.4 Hz, 1H), 2.31 (s, 3H), 2.40 (t, *J* = 7.4 Hz, 2H), 2.48 (brs, 8H), 2.95 (m, 1H), 3.61 (s, 3H), 3.72 (s, 1H), 4.43 (m, 2H), 7.04 (t, *J* = 7.3 Hz, 1H), 7.14 (d, *J* = 7.7 Hz, 1H), 7.16 (t, *J* = 7.4 Hz, 1H), 7.30 (d, *J* = 8.7 Hz, 1H), 7.33 (d, *J* = 8.9 Hz, 1H), 7.38 (s, 1H), 7.42 (d, *J* = 8.1 Hz, 1H); ^13^C NMR (150 MHz, CDCl_3_) *δ*: 18.1, 19.1, 21.0, 24.1, 24.1, 24.1, 28.0, 33.8, 36.4, 38.5, 38.6, 45.2, 45.5, 45.8, 46.0, 52.3, 52.8, 54.9, 57.7, 110.2, 113.5, 119.5, 120.6, 120.8, 121.2, 123.3, 125.3, 125.7, 127.2, 135.3, 138.9, 146.1, 146.9, 180.3; IR (KBr, cm^−1^): 3037, 2932, 2869, 2797, 1724, 1604, 1458, 1358, 1252, 1164, 742; HRMS (ESI): *m*/*z* [M + H]^+^ calcd. for C_36_H_50_N_3_O_2_: 556.3903; found: 556.3910.

#### 2.3.19. 2,3,4,4a,9,13c-Hexahydro-7-isopropyl-1,4a-dimethyl-9–(2-(4-ethylpiperazin -1-yl)butyl)-1H-dibenzo[a,c]carbazole-1-carboxylic acid methyl ester (8c)

Yellow amorphous solid; Yield: 49%; ^1^H NMR (300 MHz, CDCl_3_) *δ*: 1.05 (s, 3H), 1.09 (t, *J* = 7.5 Hz, 3H), 1.31 (d, *J* = 6.7 Hz, 3H), 1.32 (d, *J* = 6.8 Hz, 3H), 1.50–1.70 (m, 3H), 1.74 (s, 3H), 1.80–2.15 (m, 6H), 2.29 (d, *J* = 11.9 Hz, 1H), 2.35–2.44 (m, 4H), 2.48 (brs, 8H), 2.95 (m, 1H), 3.61 (s, 3H), 3.72 (s, 1H), 4.42 (m, 2H), 7.04 (t, *J* = 7.6 Hz, 1H), 7.14 (d, *J* = 7.7 Hz, 1H), 7.16 (t, *J* = 7.7 Hz, 1H), 7.30 (d, *J* = 9.2 Hz, 1H), 7.33 (d, *J* = 9.0 Hz, 1H), 7.38 (s, 1H), 7.43 (d, *J* = 8.1 Hz, 1H); ^13^C NMR (150 MHz, CDCl_3_) *δ*: 11.8, 18.1, 19.1, 21.0, 24.1, 24.1, 24.1, 28.0, 33.8, 36.4, 38.5, 38.6, 45.2, 45.5, 46.0, 52.2, 52.3, 52.6, 52.9, 57.8, 110.1, 113.4, 119.5, 120.6, 120.8, 121.2, 123.3, 125.3, 125.6, 127.2, 135.3, 138.9, 146.0, 146.9, 180.2; IR (KBr, cm^−1^): 3050, 2958, 2927, 2854, 2810, 1724, 1608, 1463, 1347, 1252, 1165, 733; HRMS (ESI): *m*/*z* [M + H]^+^ calcd. for C_37_H_52_N_3_O_2_: 570.4060; found: 570.4064.

#### 2.3.20. 2,3,4,4a,9,13c-Hexahydro-7-isopropyl-1,4a-dimethyl-9–(2-(1,4-diazepan-1-yl) butyl)-1H-dibenzo[a,c]carbazole-1-carboxylic acid methyl ester (8d)

Yellow amorphous solid; Yield: 39%; ^1^H NMR (300 MHz, CDCl_3_) *δ*: 1.05 (s, 3H), 1.30 (d, *J* = 6.3 Hz, 3H), 1.32 (d, *J* = 6.6 Hz, 3H), 1.40–1.70 (m, 3H), 1.73 (s, 3H), 1.77–2.05 (m, 8H), 2.29 (d, *J* = 11.4 Hz, 1H), 2.50 (t, *J* = 7.2 Hz, 2H), 2.58–2.70 (m, 5H), 2.95 (m, 1H), 3.08 (m, 2H), 3.17 (m, 2H), 3.61 (s, 3H), 3.71 (s, 1H), 4.45 (t, *J* = 7.7 Hz, 2H), 7.04 (t, *J* = 8.0 Hz, 1H), 7.15 (d, *J* = 7.7 Hz, 1H), 7.16 (t, *J* = 7.6 Hz, 1H), 7.30 (d, *J* = 7.8 Hz, 1H), 7.33 (d, *J* = 7.8 Hz, 1H), 7.38 (s, 1H), 7.39 (d, *J* = 7.2 Hz, 1H); ^13^C NMR (150 MHz, CDCl_3_) *δ*: 16.6, 18.2, 19.2, 21.1, 22.8, 24.2, 24.2, 27.6, 33.9, 36.5, 38.5, 38.6, 38.7, 44.5, 45.0, 45.6, 46.0, 47.7, 52.5, 53.7, 57.5, 110.3, 114.2, 119.7, 120.8, 121.1, 121.3, 123.5, 125.5, 125.9, 127.3, 135.5, 139.0, 146.2, 147.0, 180.4; IR (KBr, cm^−1^): 3394, 3041, 2958, 2930, 2864, 1721, 1603, 1462, 1382, 1361, 1248, 1127, 735; HRMS (ESI): *m*/*z* [M + H]^+^ calcd. for C_36_H_50_N_3_O_2_: 556.3903; found: 556.3909.

#### 2.3.21. 2,3,4,4a,9,13c-Hexahydro-7-isopropyl-1,4a-dimethyl-9–(2-(4-formylpiperazin-1-yl)butyl)-1H-dibenzo[a,c]carbazole-1-carboxylic acid methyl ester (8e)

Yellow amorphous solid; Yield: 53%; ^1^H NMR (300 MHz, CDCl_3_) *δ*: 1.05 (s, 3H), 1.31 (d, *J* = 6.6 Hz, 3H), 1.33 (d, *J* = 6.7 Hz, 3H), 1.50–1.72 (m, 3H), 1.74 (s, 3H), 1.76–2.10 (m, 6H), 2.25–2.45 (m, 7H), 2.96 (m, 1H), 3.33 (brs, 2H), 3.52 (brs, 2H), 3.62 (s, 3H), 3.71 (s, 1H), 4.46 (m, 2H), 7.04 (t, *J* = 7.6 Hz, 1H), 7.15 (d, *J* = 7.8 Hz, 1H), 7.16 (t, *J* = 7.8 Hz, 1H), 7.31 (d, *J* = 8.2 Hz, 1H), 7.33 (d, *J* = 8.4 Hz, 1H), 7.39 (s, 1H), 7.41 (d, *J* = 10.3 Hz, 1H), 8.00 (s, 1H, CHO); ^13^C NMR (150 MHz, CDCl_3_) *δ*: 18.1, 19.1, 21.0, 23.9, 24.1, 27.7, 33.8, 36.4, 38.5, 38.6, 39.9, 45.1, 45.5, 45.9, 52.2, 52.3, 53.5, 57.6, 110.1, 113.6, 119.5, 120.7, 120.8, 121.2, 123.3, 125.4, 125.7, 127.2, 135.4, 138.9, 146.0, 146.9, 160.6, 180.2; IR (KBr, cm^−1^): 3045, 2949, 2925, 2855, 2817, 1721, 1679, 1466, 1360, 1219, 1137, 735; HRMS (ESI): *m*/*z* [M + H]^+^ calcd. for C_36_H_48_N_3_O_3_: 570.3696; found: 570.3689.

#### 2.3.22. 2,3,4,4a,9,13c-Hexahydro-7-isopropyl-1,4a-dimethyl-9–(2-(4-phenyl piperazin-1-yl)butyl)-1H-dibenzo[a,c]carbazole-1-carboxylic acid methyl ester (8f)

Yellow amorphous solid; Yield: 49%; ^1^H NMR (300 MHz, CDCl_3_) *δ*: 1.06 (s, 3H), 1.32 (d, *J* = 7.6 Hz, 3H), 1.33 (d, *J* = 7.5 Hz, 3H), 1.60–1.70 (m, 3H), 1.75 (s, 3H), 1.80–2.10 (m, 6H), 2.29 (d, *J* = 11.8 Hz, 1H), 2.43 (t, *J* = 7.4 Hz, 2H), 2.55 (m, 4H), 2.97 (m, 1H), 3.18 (m, 4H), 3.61 (s, 3H), 3.72 (s, 1H), 4.46 (m, 2H), 6.84 (t, *J* = 7.3 Hz, 1H), 6.91 (d, *J* = 8.2 Hz, 2H), 7.04 (t, *J* = 7.6 Hz, 1H), 7.14 (d, *J* = 7.6 Hz, 1H), 7.15 (t, *J* = 7.4 Hz, 1H), 7.25 (t, *J* = 7.8 Hz, 2H), 7.30 (d, *J* = 8.1 Hz, 1H), 7.34 (d, *J* = 8.3 Hz, 1H), 7.40 (s, 1H), 7.43 (d, *J* = 8.2 Hz, 1H); ^13^C NMR (125 MHz, CDCl_3_) *δ*: 18.3, 19.3, 21.2, 24.2, 24.3, 24.3, 28.1, 34.0, 36.6, 38.7, 38.8, 45.4, 45.7, 46.1, 49.3, 52.5, 53.3, 58.0, 110.3, 113.7, 116.2, 119.7, 119.8, 120.9, 121.0, 121.4, 123.4, 125.5, 125.9, 127.5, 129.2, 135.6, 139.2, 146.2, 147.1, 151.5, 180.4; IR (KBr, cm^−1^): 3041, 2949, 2930, 2818, 1720, 1600, 1501, 1454, 1357, 1235, 1149, 757; HRMS (ESI): *m*/*z* [M + H]^+^ calcd. for C_41_H_52_N_3_O_2_: 618.4060; found: 618.4052.

#### 2.3.23. 2,3,4,4a,9,13c-Hexahydro-7-isopropyl-1,4a-dimethyl-9–(2-(4-(pyridine-2-yl) piperazin-1-yl)butyl)-1H-dibenzo[a,c]carbazole-1-carboxylic acid methyl ester (8g)

Yellow amorphous solid; Yield: 56%; ^1^H NMR (300 MHz, CDCl_3_) *δ*: 1.06 (s, 3H), 1.30 (d, *J* = 6.0 Hz, 3H), 1.33 (d, *J* = 6.0 Hz, 3H), 1.55–1.72 (m, 3H), 1.74 (s, 3H), 1.78–2.15 (m, 6H), 2.29 (d, *J* = 11.4 Hz, 1H), 2.43 (t, *J* = 7.3 Hz, 2H), 2.51 (brs, 4H), 2.96 (m, 1H), 3.53 (brs, 4H), 3.61 (s, 3H), 3.72 (s, 1H), 4.46 (brs, 2H), 6.62 (t, *J* = 8.0 Hz, 1H), 6.63 (d, *J* = 8.0 Hz, 1H), 7.04 (t, *J* = 7.5 Hz, 1H), 7.15 (d, *J* = 7.7 Hz, 1H), 7.16 (t, *J* = 7.7 Hz, 1H), 7.30 (d, *J* = 8.9 Hz, 1H), 7.33 (d, *J* = 9.0 Hz, 1H), 7.40 (s, 1H), 7.43 (d, *J* = 8.3 Hz, 1H), 7.47 (t, *J* = 7.8 Hz, 1H), 8.19 (d, *J* = 4.0 Hz, 1H); ^13^C NMR (150 MHz, CDCl_3_) *δ*: 18.2, 19.3, 21.1, 24.1, 24.2, 24.3, 28.1, 33.9, 36.5, 38.6, 38.7, 45.2, 45.3, 45.6, 46.1, 52.5, 53.0, 58.0, 107.1, 110.2, 113.4, 113.7, 119.6, 120.8, 120.9, 121.4, 123.4, 125.5, 125.8, 127.4, 135.5, 137.5, 139.1, 146.2, 147.0, 148.0, 159.6, 180.4; IR (KBr, cm^−1^): 3046, 2962, 2926, 2853, 1722, 1593, 1436, 1380, 1247, 1140, 734; HRMS (ESI): *m*/*z* [M + H]^+^ calcd. for C_40_H_51_N_4_O_2_: 619.4012; found: 619.4018.

#### 2.3.24. 2,3,4,4a,9,13c-Hexahydro-7-isopropyl-1,4a-dimethyl-9–(2-(4-benzylpiperazin -1-yl)butyl)-1H-dibenzo[a,c]carbazole-1-carboxylic acid methyl ester (8h)

Yellow amorphous solid; Yield: 54%; ^1^H NMR (300 MHz, CDCl_3_) *δ*: 1.04 (s, 3H), 1.30 (d, *J* = 6.7 Hz, 3H), 1.32 (d, *J* = 6.7 Hz, 3H), 1.50–1.70 (m, 3H), 1.74 (s, 3H), 1.76–2.10 (m, 6H), 2.29 (d, *J* = 12.4 Hz, 1H), 2.40 (t, *J* = 7.6 Hz, 2H), 2.48 (brs, 8H), 2.95 (m, 1H), 3.53 (s, 2H), 3.61 (s, 3H), 3.72 (s, 1H), 4.42 (m, 2H), 7.04 (t, *J* = 7.8 Hz, 1H), 7.14 (d, *J* = 8.0 Hz, 1H), 7.15 (t, *J* = 7.0 Hz, 1H), 7.30 (d, *J* = 8.5 Hz, 1H), 7.31 (s, 5H), 7.32 (d, *J* = 8.3 Hz, 1H), 7.38 (s, 1H), 7.42 (d, *J* = 8.3 Hz, 1H); ^13^C NMR (150 MHz, CDCl_3_) *δ*: 18.1, 19.2, 21.1, 24.1, 24.2, 24.2, 28.0, 33.8, 36.4, 38.5, 38.6, 45.2, 45.5, 46.0, 51.4, 53.0, 53.5, 57.7, 63.1, 110.3, 113.4, 119.6, 120.6, 120.9, 121.2, 123.3, 125.4, 125.7, 127.0, 127.2, 128.2, 129.2, 135.3, 138.1, 139.0, 146.0, 146.8, 180.1; IR (KBr, cm^−1^): 3024, 2932, 2872, 2806, 1722, 1603, 1455, 1348, 1283, 1162, 1009, 738; HRMS (ESI): *m*/*z* [M + H]^+^ calcd. for C_42_H_54_N_3_O_2_: 632.4216; found: 632.4207.

### 2.4. General procedure for the synthesis of compounds 10a-j

To a solution of compound **3** (1.8 g, 5.5 mmol) in 20 mL of EtOH was added 12 mmol of substituted phenylhydrazine hydrochloride and 2 mL of concentrated HCl. The mixture was refluxed for 3 h. After cooling, the mixture was poured into 100 mL of ice-cold water and extracted with CH_2_Cl_2_ (3 × 80 mL). The organic layer was combined, washed with saturated NaHCO_3_ solution and brine, dried over anhydrous Na_2_SO_4_ and concentrated to give a crude product, which was subject to a silica gel column chromatography (petroleum ethereacetone 50:1, v/v) to afford compound **4a-j**. Subsequently, to a solution of compound **4a-j** (1.75 mmol) in benzene (5 mL) were added 1,2-dibromoalkane (3.76 g, 20 mmol), tetrabutyl ammonium bromide (TBAB) (0.02 g, 0.062 mmol) and 50% NaOH solution (3 mL). The mixture was stirred at room temperature for 12 h. Then the mixture was poured into 100 mL of ice-cold water. The suspension was extracted with CH_2_Cl_2_ (3 × 80 mL). The organic layer was combined, washed with water and brine, dried over anhydrous Na_2_SO_4_ and concentrated *in vacuo*. The residue was purified by column chromatography on a silica gel column, eluting with petroleum ether-acetone (100:1, v/v) to give compounds **9a-j** (Yield: 58–69%). Further, to a solution of compound **9a-j** (0.5 mmol) in acetonitrile (15 mL) was added anhydrous K_2_CO_3_ (0.345 g, 2.5 mmol), KI (0.083 g, 0.5 mmol) and 10 mmol of anhydrous piperazine. The mixture was refluxed for 8–12 h and monitored by TLC. At the end of reaction, the mixture was poured into cold water, which was extracted by CH_2_Cl_2_ (100 mL) for three times. The organic phase was combined, washed with water and brine, dried over anhydrous Na_2_SO_4_ and concentrated *in vacuo*. The residue was subjected to silica gel chromatography, eluting with petroleum ether-acetone (100:1, v/v) to afford compounds **10a-j**.

#### 2.4.1. 2,3,4,4a,9,13c-Hexahydro-7-isopropyl-1,4a,12-trimethyl-9–(2-(piperazin-1-yl) ethyl)-1H-dibenzo[a,c]carbazole-1-carboxylic acid methyl ester (10a)

Yellow amorphous solid; Yield: 51%; ^1^H NMR (300 MHz, CDCl_3_) *δ*: 1.06 (s, 3H), 1.30 (d, *J* = 6.2 Hz, 3H), 1.32 (d, *J* = 6.2 Hz, 3H), 1.64 (m, 1H), 1.73 (s, 3H), 1.8 1–2.00 (m, 4H), 2.28 (d, *J* = 11.7 Hz, 1H), 2.42 (s, 3H), 2.58 (brs, 4H), 2.79 (m, 2H), 2.90 (m, 4H), 3.01 (m, 1H), 3.13 (brs, 1H, NH), 3.64 (s, 3H), 3.68 (s, 1H), 4.55 (m, 2H), 6.99 (d, *J* = 8.2 Hz, 1H), 7.12 (s, 1H), 7.13 (d, *J* = 9.0 Hz, 1H), 7.29 (d, *J* = 8.2 Hz, 2H), 7.43 (s, 1H); ^13^C NMR (150 MHz, CDCl_3_) *δ*: 18.2, 19.4, 21.3, 21.9, 24.2, 24.3, 34.0, 36.5, 38.6, 38.7, 43.0, 43.4, 45.6, 46.0, 50.1, 52.4, 57.1, 109.7, 113.9, 120.9, 121.1, 122.7, 123.7, 125.4, 126.4, 127.4, 128.9, 135.9, 137.5, 146.2, 147.0, 180.2; IR (KBr, cm^−1^): 2948, 2929, 2868, 1724, 1678, 1498, 1457, 1363, 1253, 1190, 1000, 826, 736; HRMS (ESI): *m*/*z* [M + H]^+^ calcd. for C_34_H_46_N_3_O_2_: 528.3590; found: 528.3595.

#### 2.4.2. 2,3,4,4a,9,13c-Hexahydro-7-isopropyl-1,4a,10-trimethyl-9–(2-(piperazin-1-yl) ethyl)-1H-dibenzo[a,c]carbazole-1-carboxylic acid methyl ester (10b)

Yellow amorphous solid; Yield: 45%; ^1^H NMR (300 MHz, CDCl_3_) *δ*: 1.14 (s, 3H), 1.30 (d, *J* = 6.4 Hz, 3H), 1.31 (d, *J* = 6.8 Hz, 3H), 1.63 (m, 1H), 1.76 (s, 3H), 1.8 0–2.10 (m, 4H), 2.22 (d, *J* = 13.7 Hz, 1H), 2.31 (brs, 4H), 2.35 (m, 2H), 2.61 (brs, 1H, NH), 2.73 (s, 3H), 2.78 (m, 4H), 2.96 (m, 1H), 3.58 (s, 3H), 3.66 (s, 1H), 4.75 (m, 2H), 6.90 (d, *J* = 6.7 Hz, 1H), 6.98 (t, *J* = 7.5 Hz, 1H), 7.13 (d, *J* = 7.4 Hz, 1H), 7.20 (d, *J* = 8.1 Hz, 1H), 7.30 (d, *J* = 8.0 Hz, 1H), 7.38 (s, 1H); ^13^C NMR (125 MHz, CDCl_3_) *δ*: 18.1, 19.4, 21.0, 21.2, 24.1, 24.2, 33.9, 36.5, 38.4, 38.8, 44.2, 45.5, 46.1, 46.2, 52.2, 52.4, 57.8, 116.6, 119.0, 120.5, 121.9, 122.1, 123.4, 124.9, 125.2, 126.9, 128.5, 138.4, 139.8, 146.2, 146.9, 180.3; IR (KBr, cm^−1^): 2953, 2926, 2855, 1724, 1680, 1493, 1459, 1382, 1253, 1188, 1081, 965, 741; HRMS (ESI): *m*/*z* [M + H]^+^ calcd. for C_34_H_46_N_3_O_2_: 528.3590; found: 528.3583.

#### 2.4.3. 2,3,4,4a,9,13c-Hexahydro-12-ethyl-7-isopropyl-1,4a-dimethyl-9–(2-(piperazin -1-yl) ethyl)-1H-dibenzo[a,c]carbazole-1-carboxylic acid methyl ester (10c)

Yellow amorphous solid; Yield: 58%; ^1^H NMR (600 MHz, CDCl_3_) *δ*: 1.05 (s, 3H), 1.25–1.31 (m, 9H), 1.64 (m, 1H), 1.74 (s, 3H), 1.82–1.98 (m, 4H), 2.28 (d, *J* = 11.5 Hz, 1H), 2.63 (m, 4H), 2.71 (q, *J* = 6.2 Hz, 2H), 2.76 (m, 1H), 2.85 (m, 1H), 2.92 (m, 4H), 2.99 (m, 1H), 3.46 (brs, 1H, NH), 3.63 (s, 3H), 3.69 (s, 1H), 4.54 (m, 2H), 7.02 (d, *J* = 8.4 Hz, 1H), 7.13 (dd, *J* = 8.0, 1.6 Hz, 1H), 7.16 (s, 1H), 7.29 (d, *J* = 8.0 Hz, 1H), 7.30 (d, *J* = 8.4 Hz, 1H), 7.41 (d, *J* = 1.3 Hz, 1H); ^13^C NMR (150 MHz, CDCl_3_) *δ*: 16.4, 18.2, 19.3, 21.3, 24.2, 24.2, 29.8, 33.9, 36.5, 38.6, 38.7, 43.4, 45.6, 45.9, 46.0, 50.2, 52.4, 57.2, 109.6, 113.9, 119.5, 121.1, 121.8, 123.6, 125.3, 126.3, 127.4, 135.6, 135.8, 137.6, 146.2, 147.0, 180.2; IR (KBr, cm^−1^): 2949, 2928, 2855, 1720, 1615, 1498, 1469, 1384, 1217, 1052, 824, 737; HRMS (ESI): *m*/*z* [M + H]^+^ calcd. for C_35_H_48_N_3_O_2_: 542.3747; found: 542.3753.

#### 2.4.4. 2,3,4,4a,9,13c-Hexahydro-7,12-diisopropyl-1,4a-dimethyl-9–(2-(piperazin-1-yl) ethyl)-1H-dibenzo[a,c]carbazole-1-carboxylic acid methyl ester (10d)

Yellow amorphous solid; Yield: 42%; ^1^H NMR (600 MHz, CDCl_3_) *δ*: 1.05 (s, 3H), 1.29 (d, *J* = 6.8 Hz, 6H), 1.30 (d, *J* = 6.5 Hz, 3H), 1.31 (d, *J* = 6.9 Hz, 3H), 1.64 (m, 1H), 1.74 (s, 3H), 1.8 2 ∼ 1.98 (m, 4H), 2.28 (d, *J* = 11.9 Hz, 1H), 2.6 0 ∼ 2.70 (m, 5H), 2.75 (m, 1H), 2.85 (m, 1H), 2.9 0 ∼ 2.98 (m, 6H), 3.62 (s, 3H), 3.69 (s, 1H), 4.53 (m, 2H), 7.05 (dd, *J* = 8.5, 1.3 Hz,1H), 7.13 (dd, *J* = 8.0, 1.6 Hz, 1H), 7.21 (s, 1H), 7.29 (d, *J* = 8.6 Hz, 1H), 7.30 (d, *J* = 8.0 Hz, 1H), 7.39 (d, *J* = 1.6 Hz, 1H); ^13^C NMR (150 MHz, CDCl_3_) *δ*: 18.2, 19.3, 21.3, 24.2, 24.7, 24.9, 34.0, 34.5, 36.5, 38.7, 38.8, 43.0, 43.4, 45.6, 45.9, 50.2, 52.5, 57.2, 109.5, 114.0, 118.0, 120.7, 121.0, 123.6, 125.3, 126.1, 127.4, 135.7, 137.6, 140.4, 146.2, 147.0, 180.3; IR (KBr, cm^−1^): 2951, 2928, 2854, 1723, 1609, 1498, 1459, 1381, 1362, 1250, 1130, 824, 736; HRMS (ESI): *m*/*z* [M + H]^+^ calcd. for C_36_H_50_N_3_O_2_: 556.3903; found: 556.3911.

#### 2.4.5. 2,3,4,4a,9,13c-Hexahydro-7-isopropyl-12-methoxy-1,4a-dimethyl-9–(2- (piperazin-1-yl)ethyl)-1H-dibenzo[a,c]carbazole-1-carboxylic acid methyl ester (10e)

Yellow amorphous solid; Yield: 55%; ^1^H NMR (300 MHz, CDCl_3_) *δ*: 1.06 (s, 3H), 1.30 (d, *J* = 7.0 Hz, 3H), 1.32 (d, *J* = 6.2 Hz, 3H), 1.67 (m, 1H), 1.75 (s, 3H), 1.80–2.00 (m, 4H), 2.30 (d, *J* = 11.8 Hz, 1H), 2.50–2.55 (m, 5H), 2.72–3.00 (m, 7H), 3.62 (s, 3H), 3.70 (s, 1H), 3.81 (s, 3H), 4.54 (m, 2H), 6.83 (d, *J* = 9.4 Hz, 1H), 6.85 (s, 1H), 7.14 (d, *J* = 8.1 Hz, 1H), 7.30 (d, *J* = 7.9 Hz, 2H), 7.44 (s, 1H); ^13^C NMR (150 MHz, CDCl_3_) *δ*: 18.2, 19.3, 21.3, 24.2, 24.2, 34.0, 36.4, 38.7, 38.7, 43.0, 43.3, 45.7, 45.9, 50.1, 52.6, 55.8, 57.2, 102.8, 110.5, 111.6, 114.0, 121.1, 123.7, 125.5, 126.3, 127.3, 134.3, 136.3, 146.2, 147.0, 154.2, 180.5; IR (KBr, cm^−1^): 2957, 2929, 2855, 1719, 1677, 1616, 1499, 1453, 1363, 1223, 1049, 826, 734; HRMS (ESI): *m*/*z* [M + H]^+^ calcd. for C_34_H_46_N_3_O_3_: 544.3539; found: 544.3531.

#### 2.4.6. 2,3,4,4a,9,13c-Hexahydro-7-isopropyl-10-methoxy-1,4a-dimethyl-9–(2- (piperazin-1-yl)ethyl)-1H-dibenzo[a,c]carbazole-1-carboxylic acid methyl ester (10f)

Yellow amorphous solid; Yield: 51%; ^1^H NMR (600 MHz, CDCl_3_) *δ*: 1.08 (s, 3H), 1.29 (d, *J* = 6.6 Hz, 3H), 1.30 (d, *J* = 7.0 Hz, 3H), 1.61 (m, 1H), 1.73 (s, 3H), 1.80–2.00 (m, 4H), 2.28 (d, *J* = 12.0 Hz, 1H), 2.50–2.65 (m, 6H), 2.80–3.00 (m, 6H), 3.58 (s, 3H), 3.64 (s, 1H), 3.94 (s, 3H), 4.75 (m, 1H), 4.97 (m, 1H), 6.61 (d, *J* = 7.1 Hz, 1H), 6.92 (d, *J* = 8.1 Hz, 1H), 6.96 (d, *J* = 7.6 Hz, 1H), 7.12 (dd, *J* = 8.0, 1.6 Hz, 1H), 7.28 (d, *J* = 8.2 Hz, 1H), 7.39 (d, *J* = 1.2 Hz, 1H); ^13^C NMR (150 MHz, CDCl_3_) *δ*: 18.1, 19.3, 21.2, 24.1, 24.2, 33.8, 36.5, 38.5, 38.7, 43.2, 44.6, 45.5, 46.0, 49.9, 52.4, 55.4, 58.5, 102.6, 113.9, 115.3, 120.2, 121.7, 123.5, 125.3, 127.0, 128.7, 129.1, 137.1, 146.1, 146.9, 147.7, 180.3; IR (KBr, cm^−1^): 2957, 2929, 2855, 1722, 1678, 1608, 1568, 1458, 1365, 1259, 1046, 825, 732; HRMS (ESI): *m*/*z* [M + H]^+^ calcd. for C_34_H_46_N_3_O_3_: 544.3539; found: 544.3543.

#### 2.4.7. 2,3,4,4a,9,13c-Hexahydro-12-ethoxy-7-isopropyl-1,4a-dimethyl-9–(2- (piperazin-1-yl)ethyl)-1H-dibenzo[a,c]carbazole-1-carboxylic acid methyl ester (10g)

Yellow amorphous solid; Yield: 53%; ^1^H NMR (500 MHz, CDCl_3_) *δ*: 1.11 (s, 3H), 1.30 (d, *J* = 7.6 Hz, 3H), 1.31 (d, *J* = 7.8 Hz, 3H), 1.52 (t, *J* = 7.0 Hz, 3H), 1.63 (m, 1H), 1.74 (s, 3H), 1.80–2.05 (m, 4H), 2.29 (d, *J* = 11.6 Hz, 1H), 2.41 (brs, 4H), 2.50–2.60 (m, 4H), 2.80–2.90 (m, 3H), 2.94 (m, 1H), 3.59 (s, 3H), 3.66 (s, 1H), 4.20 (q, *J* = 7.0 Hz, 2H), 4.80 (m, 1H), 5.07 (m, 1H), 6.60 (d, *J* = 5.7 Hz, 1H), 6.92 (s, 1H), 6.93 (d, *J* = 6.1 Hz, 1H), 7.12 (d, *J* = 7.8 Hz, 1H), 7.29 (d, *J* = 8.1 Hz, 1H), 7.42 (s, 1H); ^13^C NMR (125 MHz, CDCl_3_) *δ*: 15.1, 18.1, 19.3, 21.2, 24.0, 24.2, 33.8, 36.5, 38.5, 38.7, 43.4, 45.4, 45.5, 46.0, 50.4, 52.3, 58.6, 63.8, 103.4, 113.8, 115.3, 120.2, 121.8, 123.4, 125.2, 127.1, 128.9, 129.1, 137.2, 146.0, 146.9, 147.0, 180.2; IR (KBr, cm^−1^): 2945, 2928, 2855, 1720, 1615, 1498, 1469, 1383, 1217, 1052, 824, 737; HRMS (ESI): *m*/*z* [M + H]^+^ calcd. for C_35_H_48_N_3_O_3_: 558.3696; found: 558.3701.

#### 2.4.8. 2,3,4,4a,9,13c-Hexahydro-10-ethoxy-7-isopropyl-1,4a-dimethyl-9–(2- (piperazin-1-yl)ethyl)-1H-dibenzo[a,c]carbazole-1-carboxylic acid methyl ester (10h)

Yellow amorphous solid; Yield: 58%; ^1^H NMR (500 MHz, CDCl_3_) *δ*: 1.06 (s, 3H), 1.31 (d, *J* = 7.8 Hz, 3H), 1.33 (d, *J* = 7.3 Hz, 3H), 1.44 (t, *J* = 7.0 Hz, 3H), 1.67 (dt, *J* = 12.9, 2.0 Hz, 1H), 1.75 (s, 3H), 1.8 0 ∼ 2.05 (m, 4H), 2.29 (d, *J* = 11.9 Hz, 1H), 2.52 (brs, 4H), 2.7 0 ∼ 2.95 (m, 7H), 2.96 (m, 1H), 3.61 (s, 3H), 3.70 (s, 1H), 4.03 (m, 2H), 4.53 (m, 2H), 6.84 (dd, *J* = 8.2, 1.8 Hz, 1H), 6.85 (s, 1H), 7.14 (d, *J* = 8.0 Hz, 1H), 7.28–7.32 (m, 2H), 7.45 (s, 1H); ^13^C NMR (125 MHz, CDCl_3_) *δ*: 15.2, 18.3, 19.3, 21.3, 24.2, 24.3, 34.1, 36.6, 38.8, 38.8, 43.4, 45.6, 45.8, 46.0, 52.5, 54.5, 58.2, 64.1, 103.7, 110.6, 112.3, 113.6, 121.5, 123.5, 125.3, 126.1, 127.4, 134.5, 136.2, 146.3, 147.0, 153.4, 180.6; IR (KBr, cm^−1^): 2951, 2927, 2852, 1717, 1612, 1493, 1460, 1381, 1221, 1051, 812, 758; HRMS (ESI): *m*/*z* [M + H]^+^ calcd. for C_35_H_48_N_3_O_3_: 558.3696; found: 558.3690.

#### 2.4.9. 2,3,4,4a,9,13c-Hexahydro-12-fluoro-7-isopropyl-1,4a-dimethyl-9–(2- (piperazin-1-yl)ethyl)-1H-dibenzo[a,c]carbazole-1-carboxylic acid methyl ester (10i)

Yellow amorphous solid; Yield: 49%; ^1^H NMR (500 MHz, CDCl_3_) *δ*: 1.05 (s, 3H), 1.31 (d, *J* = 7.8 Hz, 3H), 1.33 (d, *J* = 7.6 Hz, 3H), 1.65 (t, *J* = 13.0 Hz, 1H), 1.72 (s, 3H), 1.80–2.00 (m, 4H), 2.20 (brs, 1H, NH), 2.29 (d, *J* = 11.7 Hz, 1H), 2.50 (m, 4H), 2.80 (m, 2H), 2.86 (m, 4H), 2.96 (m, 1H), 3.67 (s, 3H), 3.68 (s, 1H), 4.55 (m, 2H), 6.90 (dt, *J* = 8.8, 1.8 Hz, 1H), 7.01 (dd, *J* = 10.2, 1.5 Hz, 1H), 7.16 (d, *J* = 7.8 Hz, 1H), 7.30 (d, *J* = 8.3 Hz, 1H), 7.33 (dd, *J* = 8.8, 4.6 Hz, 1H), 7.46 (s, 1H); ^13^C NMR (125 MHz, CDCl_3_) *δ*: 18.2, 19.1, 21.3, 24.1, 24.3, 34.1, 36.5, 38.7, 43.6, 45.5, 45.9, 46.0, 52.5, 55.0, 58.2, 105.8 (d, *J* = 25.4 Hz), 109.2 (d, *J* = 25.9 Hz), 110.5 (d, *J* = 9.7 Hz), 113.7 (d, *J* = 4.6 Hz), 121.7, 123.5, 125.7, 126.0 (d, *J* = 9.8 Hz), 127.0, 135.9, 137.3, 146.4, 147.2, 157.9 (d, *J* = 231.6 Hz), 180.0; IR (KBr, cm^−1^): 2948, 2930, 2869, 1723, 1679, 1616, 1497, 1456, 1382, 1243, 1125, 1037, 823; HRMS (ESI): *m*/*z* [M + H]^+^ calcd. for C_33_H_43_FN_3_O_2_: 532.3339; found: 532.3345.

#### 2.4.10. 2,3,4,4a,9,13c-Hexahydro-12-chloro-7-isopropyl-1,4a-dimethyl-9–(2- (piperazin-1-yl)ethyl)-1H-dibenzo[a,c]carbazole-1-carboxylic acid methyl ester (10j)

Yellow amorphous solid; Yield: 58%; ^1^H NMR (600 MHz, CDCl_3_) δ: 1.05 (s, 3H), 1.27 (d, *J* = 7.0 Hz, 3H), 1.29 (d, *J* = 6.8 Hz, 3H), 1.63 (m, 1H), 1.71 (s, 3H), 1.80–1.97 (m, 4H), 2.27 (d, *J* = 12.7 Hz, 1H), 2.64 (m, 4H), 2.7 0 ∼ 2.80 (m, 3H), 2.91 (m, 4H), 2.98 (m, 1H), 3.65 (s, 1H), 3.71 (s, 3H), 4.51 (m, 1H), 4.62 (m, 1H), 7.10 (dd, *J* = 8.7, 1.9 Hz, 1H), 7.16 (dd, *J* = 8.1, 1.6 Hz, 1H), 7.25 (s, 1H), 7.28 (d, *J* = 1.9 Hz, 1H), 7.30 (d, *J* = 8.2 Hz, 1H), 7.37 (d, *J* = 1.5 Hz, 1H); ^13^C NMR (150 MHz, CDCl_3_) *δ*: 18.1, 19.0, 21.2, 24.1, 24.2, 33.9, 36.4, 38.5, 43.3, 45.2, 45.4, 45.9, 52.4, 53.7, 57.8, 110.9, 113.3, 120.1, 121.0, 121.5, 123.5, 125.4, 125.8, 126.8, 137.0, 137.4, 146.3, 147.1, 162.5, 179.7; IR (KBr, cm^−1^): 2952, 2929, 2856, 1725, 1670, 1607, 1495, 1455, 1360, 1262, 1111, 968, 737; HRMS (ESI): *m*/*z* [M + H]^+^ calcd. for C_33_H_43_ClN_3_O_2_: 548.3044; found: 548.3038.

### 2.5. Biological evaluation

#### 2.5.1. Cell lines and culture

Three human hepatocarcinoma cell lines (SMMC-7721, HepG2 and Hep3B) and normal hepatocyte cell line (QSG-7701) were maintained in Dulbecco Modified Eagle Medium (DMEM) containing 4.0 mM L-Glutamine and 4500 mg/l Glucose supplemented with 10% (v/v) foetalbovine serum (FBS) and 100 unites/mL penicillin/streptomycin at 37 °C in humidified atmosphere of 5% CO_2_ and 95% air.

#### 2.5.2. Cytotoxic assay

The *in vitro* cytotoxic activities of the carbazole derivatives of DHA were evaluated against three human hepatocarcinoma cell lines (SMMC-7721, HepG2 and Hep3B) and a normal human hepatocyte cell line (QSG-7701) *via* the MTT colorimetric method^39^. Briefly, SMMC-7721, HepG2, Hep3B and QSG-7701 cells were harvested at log phase of growth and seeded in 96-well plates (100 µL/well at a density of 2 × 10^5^ cells/mL). After 24 h incubation at 37 °C and 5% CO_2_ to allow cell attachment, cultures were exposed to various concentrations of the isolated compounds for 48 h. Finally, MTT solution (2.5 mg/mL in PBS) was added (40 µL/well). Plates were further incubated for 4 h at 37 °C, and the formazan crystals formed were dissolved by adding 150 µL/well of DMSO. Absorption at 570 nm was measured with an ELISA plate reader. The results were expressed as IC_50_ values (mean, *n* = 3), which was defined as the concentration at which 50% survival of cells was discerned. Doxorubicin was co-assayed as positive control.

#### 2.5.3. *In vitro* MEK1 inhibition assay

An *in vitro* kinase assay of MEK1 was performed using ADP-Glo kinase assay (Promega, Madison, WI, USA) according to the manufacturer’s protocol. Briefly, the kinase reaction was conducted in a 5 µL mixture [25 mM Tris-HCl (pH 7.5), 25 mH MgCl_2_, 2 mM dithiothretol, 10 µM ATP, 0.02% Triton X-100, 200 ng of recombinant GST-MEK1 protein (Active) and 200 ng of GST-ERK2 (Inactive) protein (Carna Biosciences, Japan)] with or without various concentrations of tested compounds at 22 °C for 30 min. Reactions were stopped by adding 5 µL of ADP-Glo reagent to each well. After incubating at 22 °C for 40 min, 10 µL of the kinase detection reagent was added and the plates were incubated for another 30 min at 22 °C in the dark. The reaction mixture was analysed by EnSpire (PerkinElmer, Waltham, MA, USA). U0126 was used as the positive control for MEK1 inhibition.

#### 2.5.4. Molecular docking

The molecular modelling of compound **10g** was performed with Schrödinger Suite 2015–1 (Schrödinger LLC., New York, NY, USA). The crystal structure of the MEK1 (PDB ID: 3EQF) was downloaded from Protein Data Bank (PDB) and prepared using the Protein Preparation Wizard workflow from Schrödinger Suite, including the optimisation of hydrogen bond network and a short energy minimisation with position restraints on heavy atoms using OPLS_2005 force field. The docking grid was generated according to the initial ligand K252A. Then the target compounds were freely docked into the designated binding site using the standard protocol implemented in Maestro v 10.1 (Schrödinger LLC, Cambridge, MA, USA). Van der Waals (vdW) scaling of 0.8 and partial cut-off of 0.15 were set to soften the potential for non-polar sites, and no constraints were specified. The best docked pose ranked by Glide Score value was recorded, and saved for each ligand. The structures of complexes were analysed for interaction modes, and the binding pose of compound **10g** with MEK1 kinase was displayed using Discovery studio 3.5 client.

#### 2.5.5. Cell cycle analysis

Cell cycle distributions in HepG2 cells were determined through propidium iodide (PI) staining and analysed by flow cytometry. HepG2 cells were seeded into a six-well plate at 5 × 10^5^ cell/mL and treated with different concentrations of compound **10g** for 48 h. After treatment, cells were detached with 0.25% trypsin, harvested by centrifugation, washed twice with ice-cold PBS and then fixed and permeabilised with ice-cold 70% ethanol at 4 °C overnight. Ethanol was removed and the cells were washed twice with ice-cold PBS. After this, the cells were treated with 100 µL of RNase (100 µg/mL) at 37 °C for 30 min, followed by incubation with 400 µl of DNA staining solution (PI) (1 mg/mL) in the dark at 4 °C for 30 min. The samples were analyzed by a flow cytometer (Becton-Dickinson FACSCalibur, NJ, USA) and data were analysed using the FlowJo software (Becton-Dickinson & Co, Totowa, NJ, USA).

#### 2.5.6. Annexin V-FITC/PI dual staining assay

The extent of apoptosis was quantitatively measured using Annexin V-FITC/PI dual staining assay. HepG2 cells were seeded into a six-well plate at 5 × 10^5^ cells per well in 10% foetal calf serum (FBS)-DMEM into six-well plates and treated with different concentrations of the indicated compound **10g** for 48 h. The cells were detached with 0.25% trypsin, washed with ice-cold PBS for twice and then resuspended in 1 × Binding buffer (0.1 M Hepes/NaOH (pH 7.4), 1.4 M NaCl, 25 mM CaCl_2_). The cells were stained with 5 µL of Annexin V-FITC and 5 µL of PI (propidium iodide) to each tube. The cells were gently vortexed and incubated in the dark at room temperature for 15 min and then keep them at 4 °C. The samples were analysed by a flow cytometer (Becton-Dickinson FACSCalibur, NJ, USA) and data were analysed using the FlowJo software (Becton-Dickinson & Co, Totowa, NJ, USA).

#### 2.5.7. ROS generation assay

Reactive oxygen species (ROS) generation assay was performed by using the reactive oxygen species assay kit (Beyotime Biotech., China). Intracellular ROS generation was tested through dichlorodihydro fluorescein diacetate (DCFH-DA) assay. DCFH-DA is taken up by HepG2 cells, and then activated by esterase-mediated cleavage of acetate to form dichlorodihydro fluorescein (DCFH), which is trapped in the cells. DCFH is converted to fluorescein DCF in the presence of ROS. HepG2 cells were seeded in six-well plates and incubated with different concentrations of compound **10g** for 24 h. After removing the compound solution, cells were treated with 10 µM of DCFH-DA at 37 °C for 20 min. Subsequently, the cells were washed with PBS for three times and then exposed to light. Immediately after light exposure, cell images were acquired through an inverted fluorescence microscope (Olympus 1X71 Inverted System Microscope, Olympus, Tokyo, Japan).

#### 2.5.8. Mitochondrial membrane potential assay

The JC-1 mitochondrial membrane potential assay kit (Keygene Biotech., China) was employed to measure mitochondrial depolarization in HepG2 cells. Briefly, cells cultured in six-well plates after indicated treatments were incubated with an equal volume of JC-1 staining solution (5 µg/mL) at 37 °C for 20 min and rinsed twice with PBS. Mitochondrial membrane potentials were monitored by determining the relative amounts of dual emissions from mitochondrial JC-1 monomers or aggregates using flow cytometry (Becton-Dickinson FACSCalibur, New York, USA). Mitochondrial depolarization is indicated by an increase in the pencentage of cells with low ΔΨm (green fluorescence, lower right quadrant) compared with cells with high ΔΨm (red fluorescence, upper right quadrant).

#### 2.5.9. Lactate dehydrogenase (LDH) leakage assay

The cell membrane integrity was determined by LDH leakage assay by using a LDH assay kit (Beyotime, China). In brief, HepG2 cells were plated on 96-well plates at the density of 5 × 10^3^ cells per well and allowed to attach overnight. After being incubated with compound **10g** for 24 h, the supernatants were collected and centrifuged at the speed of 1000 rpm and were subjected to LDH detection as the description in the manual. The absorbance at 490 nm was measured by a Cytation 3 Cell Imaging Multi-Mode Reader (BioTek Instruments, Inc., Winooski, VT, USA).

#### 2.5.10. Western blot analysis

HepG2 cells were seeded at a density of 5 × 10^6^ cells per well and attached for 8 h, and then treated with different concentrations of compound **10g** for 48 h. After the treatment, the cells were harvested and washed twice with PBS. The harvested cells were lysed with radio-immunoprecipitation assay (RIPA) lysis buffer (Beyotime Biotech., Nantong, China) with 1% cocktail (Sigma-Aldrich, USA). Whole-cell protein lysates were prepared and centrifuged at 12,000 rpm for 10 min at 4 °C. The total proteins were determined using Bradford reagent (Bio-Rad Laboratories, Inc., USA). Exactly 40 µg of protein per lane was separated through sodium dodecyl sulfate-polyacrylamide gel electrophoresis and then transferred onto a polyvinylidene difluoride membrane (Millipore, Bedford, MA, USA). The membranes were incubated with each antibody and detected through immunoblot analysis. All of the antibodies were purchased from Cell signaling Technology, Inc. (Boston, MA, USA) and diluted in accordance with the manufacturer’s instruction. Proteins were visualized using a C-Digit® imaging system (LI-COR Biosciences, Lincoln, NE, USA).

## 3. Results and discussion

### 3.1. Chemistry

**Scheme 1.**
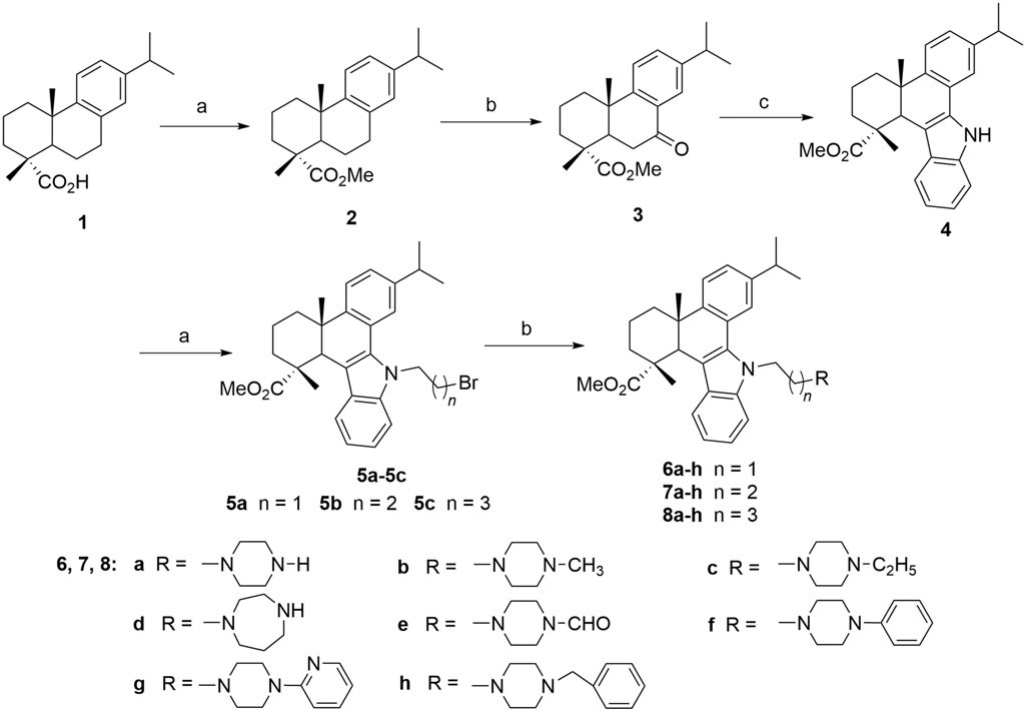
Synthetic route of target compounds **6a-h**, **7a-h** and **8a-h** from dehydroabietic acid. (a) (i) SOCl_2_, benzene, reflux, 3 h, (ii) MeOH, reflux, 2 h; (b) CrO_3_, AcOH, Ac_2_O, 0 °C to rt, 12 h; (c) phenylhydrazine hydrochloride, EtOH, conc. HCl, reflux, 3 h; (d) 1,2-dibromoethane, 1,3-dibromopropane or 1,4-dibromobutane, TBAB, NaOH, benzene, H_2_O, rt, 12 h; (e) *N*-substituted piperazine, K_2_CO_3_, KI, MeCN, reflux, 8 ∼ 12 h.

**Scheme 2.**
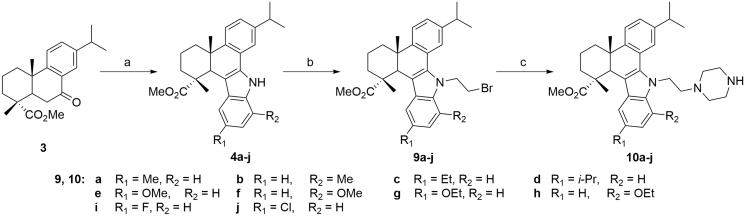
Synthetic route of target compounds **10a-j** from the intermediate **3**. (a) Substituted phenylhydrazine hydrochloride, EtOH, conc. HCl, reflux, 3 h; (b) 1,2-dibromoethane, TBAB, NaOH, benzene, H_2_O, rt, 12 h; (c) piperazine, K_2_CO_3_, KI, MeCN, reflux, 8 ∼ 12 h.

The reaction sequences employed for the synthesis of the target compounds (**6a-h**, **7a-h**, **8a-h** and **10a-j**) was outlined in Schemes 1 and 2, according to the previous studies^35^^,^^40^. Initially, methyl 7-oxo-dehydroabietate (**3**) was synthesised from the starting material dehydroabietic acid (**1**) through methyl esterification and oxidation by CrO_3_. Then compound **3** was converted to the carbazole derivative (**4**) by reacting with phenylhydrazine through Fisher indole reaction. Subsequently, the intermediate **4** was treated with 1,2-, 1,3- or 1,4-dibromoalkanes, NaOH and TBAB to give the *N*-bromoalkyl derivatives **5a-c** (Scheme 1, *n* = 1–3), which were further reacted with different *N*-substituted piperazines and homopiperazine in the presence of K_2_CO_3_, KI in acetonitrile to yield three series of *N*-substituted carbazole derivatives of **6a-h**, **7a-h** and **8a-h** with different linkers and *N*-containing heterocyclic moieties (Scheme 1).

Subsequently, to explore the relationships between the substituent on indole moiety and anticancer activity, compounds **10a-j** with different substituents on the indole benzene rings were also synthesised according to Scheme 2. Briefly, compound **3** was reacted with different substituted phenylhydrazines to afford a series of carbazole derivatives with different substituents on indole moieties (**4a-j**), which were converted to the corresponding *N*-bromoethyl derivatives **9a-j** and then *N*-(piperazin-1-yl)ethyl derivatives **10a-j** through similar two-step procedures. The structures of all the synthesised compounds were characterized by their IR, ESI-MS, ^1^H NMR and ^13^C NMR spectral data analysis (Supplementary Figures S1–S68).

### 3.2. *In vitro* cytotoxic activity

The *in vitro* cytotoxic activities of all the target compounds were evaluated by MTT assay against three human hepatocarcinoma cell lines (SMMC-7721, HepG2 and Hep3B) and a normal human hepatocyte cell line (QSG-7701). Doxorubicin was co-assayed as the positive control. The results expressed as IC_50_ values for these compounds were summarised in Tables 1 and 2.

**Table 1.** IC_50_ values of compounds **6a-t**, **7 m-t** and **8 m-t** against two hepatocarcinoma cell lines (SMMC-7721, HepG2 and Hep3B) and normal hepatocyte cell line (QSG-7701).

Compound	IC_50_ value (μM)			
	SMMC-7721	HepG2	Hep3B	QSG-7701
**6a**	5.20 ± 0.21	2.28 ± 0.19	0.82 ± 0.08	8.75 ± 0.65
**6b**	13.2 ± 0.76	11.7 ± 0.58	6.78 ± 0.42	27.53 ± 1.87
**6c**	14.00 ± 2.10	17.40 ± 1.73	10.97 ± 0.65	41.05 ± 3.69
**6d**	7.01 ± 0.74	5.87 ± 0.42	6.31 ± 0.33	22.1 ± 2.75
**6e**	17.27 ± 1.78	15.65 ± 1.61	11.42 ± 0.53	>50
**6f**	>50	>50	>50	NT
**6g**	>50	>50	>50	NT
**6h**	>50	>50	>50	NT
**7a**	5.10 ± 0.18	3.10 ± 0.45	1.37 ± 0.20	17.22 ± 1.89
**7b**	6.80 ± 0.72	29.00 ± 1.29	5.98 ± 0.39	>50
**7c**	>50	>50	40.72 ± 2.62	NT
**7d**	10.34 ± 1.02	8.68 ± 1.13	12.83 ± 0.67	32.79 ± 3.22
**7e**	23.34 ± 2.35	16.85 ± 1.58	20.03 ± 1.22	>50
**7f**	>50	>50	>50	NT
**7g**	>50	>50	>50	NT
**7h**	>50	>50	>50	NT
**8a**	6.10 ± 0.47	4.80 ± 0.32	2.89 ± 0.27	23.38 ± 2.97
**8b**	34.90 ± 3.35	19.60 ± 2.91	18.34 ± 1.02	>50
**8c**	>50	>50	>50	NT
**8d**	12.77 ± 0.84	11.52 ± 1.23	14.38 ± 0.77	43.65 ± 4.03
**8e**	32.12 ± 2.78	41.17 ± 3.56	38.02 ± 1.75	>50
**8f**	>50	>50	>50	NT
**8g**	>50	>50	>50	NT
**8h**	>50	>50	>50	NT
Doxorubicin	1.13 ± 0.11	2.38 ± 0.29	1.02 ± 0.09	13.78 ± 0.53

The results are expressed as mean value ± SD.

NT: Not tested.

**Table 2.** IC_50_ values of compounds **10a-j** against two hepatocarcinoma cell lines (SMMC-7721, HepG2, and Hep3B) and normal hepatocyte cell line (QSG-7701).

Compound	IC_50_ value (μM)			
	SMMC-7721	HepG2	Hep3B	QSG-7701
**10a**	1.73 ± 0.22	3.05 ± 0.37	1.17 ± 0.12	16.32 ± 1.56
**10b**	5.52 ± 0.47	6.11 ± 0.38	5.03 ± 0.31	21.89 ± 1.72
**10c**	6.56 ± 0.19	7.27 ± 0.51	5.84 ± 0.36	22.19 ± 1.81
**10d**	10.51 ± 0.48	8.42 ± 0.39	8.85 ± 0.42	30.98 ± 2.39
**10e**	3.02 ± 0.21	3.73 ± 0.19	4.38 ± 0.35	15.32 ± 1.13
**10f**	2.03 ± 0.15	3.15 ± 0.13	2.23 ± 0.16	17.29 ± 0.78
**10g**	1.39 ± 0.13	0.51 ± 0.09	0.73 ± 0.08	12.52 ± 0.58
**10h**	2.21 ± 0.17	4.87 ± 0.48	1.78 ± 0.12	18.87 ± 1.09
**10i**	4.32 ± 0.27	3.91 ± 0.34	3.32 ± 0.20	23.67 ± 1.53
**10j**	2.49 ± 0.18	2.88 ± 0.23	3.75 ± 0.34	12.23 ± 1.01
Doxorubicin	1.13 ± 0.11	2.38 ± 0.29	1.02 ± 0.09	13.78 ± 0.53

The results are expressed as mean value ± SD.

As shown in Table 1, compounds **6a-h**, **7a-h** and **8a-h** displayed variable cytotoxic activities against three cancer cells. Among them, compounds **6a**, **7a** and **8a** with piperazine moieties, **6b** and **7b** with *N*-methylpiperazine moieties, **6d** and **7d** containing homopiperazine moieties revealed strong inhibitory activities with IC_50_ < 10 µM against at least one hepatocarcinoma cell line. Specially, compound **6a** with *N*-(piperazin-1-yl)ethyl substituent emerged as the most potent cytotoxic agent against SMMC-7721, HepG2 and Hep3B cells with IC_50_ values of 5.20 ± 0.21, 2.28 ± 0.19 and 0.82 ± 0.08 µM, respectively, equipotent to those of doxorubicin (IC_50_: 1.13 ± 0.11, 2.38 ± 0.29 and 1.02 ± 0.09 µM, respectively). Notably, the compound was substantially less cytotoxic to normal hepatocyte cells QSG-7701 (8.75 ± 0.65 µM). In addition, its analog **7a** and **8a** with 3 C and 4 C chain linker also showed promising cytotoxic activities (IC_50_: 1.37–5.10 µM and 2.89–6.10 µM, respectively) compared with compound **6a**. Further, *N*-formylpiperazine derivatives (**6e, 7e** and **8e**) displayed moderate inhibitions to three cancer cell lines. Compound **6c** bearing *N*-ethylpiperazine also showed moderate activity while its analogs **7c** and **8c** displayed weak or no inhibitions to three cancer cell lines. One the other hand, all the derivatives with *N*-phenyl, *N*-pyridinyl and *N*-benzyl piperazine moieties (**6f-8f**, **6 g-8g** and **6 h-8h**) appeared to be inactive against three cancer cells (IC_50_ >50 µM).

From the results, it could be indicated that the cytotoxic activities of these derivatives were significantly affected by the piperazine moieties introduced to the side chain. For compounds **6a-h**, the order of cytotoxicities of these derivatives could be generally expressed as: piperazine > homopiperazine > *N*-methylpiperazine > *N*-ethyl, *N*-formylpiperazine > *N*-phenyl, *N*-pyridinyl-, and *N*-benzylpiperazine derivatives. Similar relationships could also be observed for compounds **7a-h** and **8a-h**. These results indicated that the introduction of alkyl, acyl or aryl substituents, especially bulky aryl groups on the nitrogen atom of piperazine moiety will significantly reduce the anticancer activity. On the other hand, the length of alkyl side chain also substantially affected the cytotoxicity. In general, the cytotoxic activities of compounds **6a-h** with 2 C linkers appeared to be stronger than those of compounds **7a-h** with 3 C linkers, which were markedly stronger than **8a-h** with 4 C linkers. These results suggested that the *N*-(piperazin-1-yl)ethyl side chain with piperazine heterocycle and ethyl linker proved to be most beneficial to the cytotoxic activity, and compound **6a** (QC2) was still chosen for further structural modification.

The effects of substituents on the indole benzene ring on the cytotoxicity were also explored. Compounds **10a-j** with different substituted indole moieties were synthesised and screened for their *in vitro* anticancer activities against SMMC-7721, HepG2 and Hep3B cells. As shown in Table 2, compounds **10a-j** all exhibited strong cytotoxic activities with IC_50_ values below 10 µM. Among them, compounds **10a**, **10e**, **10f**, **10g**, **10h** and **10j** displayed relatively higher anticancer potency than other derivatives, which indicated that methyl, methoxyl, ethoxyl and chloro groups anchored on the indole moiety were more beneficial to the anticancer activity. In addition, the derivatives (**10a** and **10g**) containing 12-Me and 12-OEt generally showed greater cytotoxic activities than their analogs (**10b** and **10h**) with same substituents at C-10, while compound **10f** with 10-OMe substituent was relatively more active than compound **10e** with 12-OMe. Especially, compound **10g** with 12-OEt substituent exhibited the most potent anticancer activity against SMMC-7721, HepG2 and Hep3B cells with IC_50_ values of 1.39 ± 0.13, 0.51 ± 0.09 and 0.73 ± 0.08 µM, respectively. Compared with lead compound **6a** (QC2) and the positive control doxorubicin, it exhibited considerably more potent anticancer activities against three cancer cells and lower cytotoxicity to normal hepatocyte cell line QSG-7701 (IC_50_: 12.52 ± 0.58 µM). Because of its significant anticancer property, compound **10g** was selected for further inverstigations on its anticancer mechanisms.

### 3.3. MEK1 inhibitory activity

The inhibitory activities of selected compounds (**6a** and **10a-j**) against MEK1 were evaluated by the Raf/MEK/ERK cascade kinase assay using recombinant proteins. The potent MEK1 inhibitor AZD6244 was co-assayed as positive control. The results were summarised in Table 3. It can be found that these compounds exhibited diverse inhibitory activities. Specifically, the lead compound **6a** showed weak MEK1 inhibitory activity, and compounds **10a-d** and **10i-k** displayed mild or even no activities. In contrast, compounds **10e-h** with OMe and OEt substituents demonstrated strong inhibitory activities. Among them, compound **10g** showed the most potent inhibitory activity with IC_50_ of 0.11 ± 0.02 µM, near to the positive control AZD6244 with IC_50_ of 0.029 ± 0.003 µM. These results indicated that the derivatives with potent antiproliferative activities generally showed significant MEK1 inhibitory activities in this assay. Therefore, the antiproliferative effects of these derivatives were probably correlated with their MEK1 kinase inhibitory activity.

**Table 3.** MEK1 inhibitory activities of compounds **6a** and **10a-j**. The results are expressed as mean value ± SD.

Compound	IC_50_ value (μM)
**6a**	13.21 ± 0.73
**10a**	6.36 ± 0.51
**10b**	>20
**10c**	>20
**10d**	>20
**10e**	1.28 ± 0.06
**10f**	0.23 ± 0.03
**10g**	0.11 ± 0.02
**10h**	1.63 ± 0.12
**10i**	>20
**10j**	5.62 ± 0.48
**10k**	>20
**AZD6244**	0.029 ± 0.003

**Figure 3.**
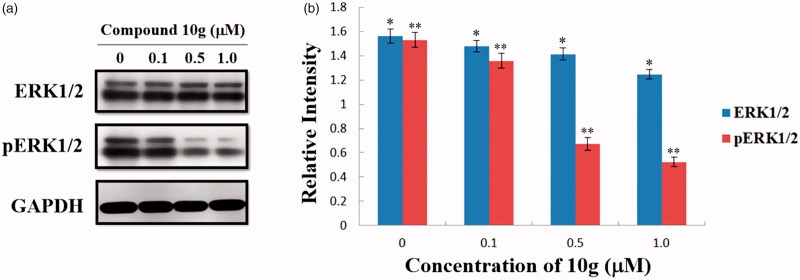
(a) Effects of compound **10g** on the expression of ERK and pERK in HepG2 cells. HepG2 cells were treated with compound **10g** (0, 0.1, 0.5 and 1.0 μM) for 48 h; (b) The expression level of ERK1/2 and pERK1/2 in HepG2 cells. *p < 0.001; **p < 0.001.

Subsequently, to evaluate the MEK inhibition of compound **10g** in HepG2 cells, western blot analyses were also carried out to test the change of levels of ERK and phosphorylated ERK (pERK) in compound **10g**-treated HepG2 cells. As shown in Figure 3, the expression levels of ERK1/2 decreased slightly, while the levels of pERK1/2 were significantly downregulated by compound **10g** in a dose-dependent manner. After treatment with different concentrations of **10g** (0.1, 0.5 and 1.0 µM) for 48 h, the expression levels of pERK1/2 were reduced to 88.8%, 43.8% and 34.2% of the control group, respectively. As a result, the immunoblot analyses demonstrated that compound **10g** could significantly inhibit MEK catalytic activity in HepG2 cells, therefore could suppress the phosphorylation and activation of the downstream ERK proteins.

### 3.4. Molecular docking

**Figure 4.**
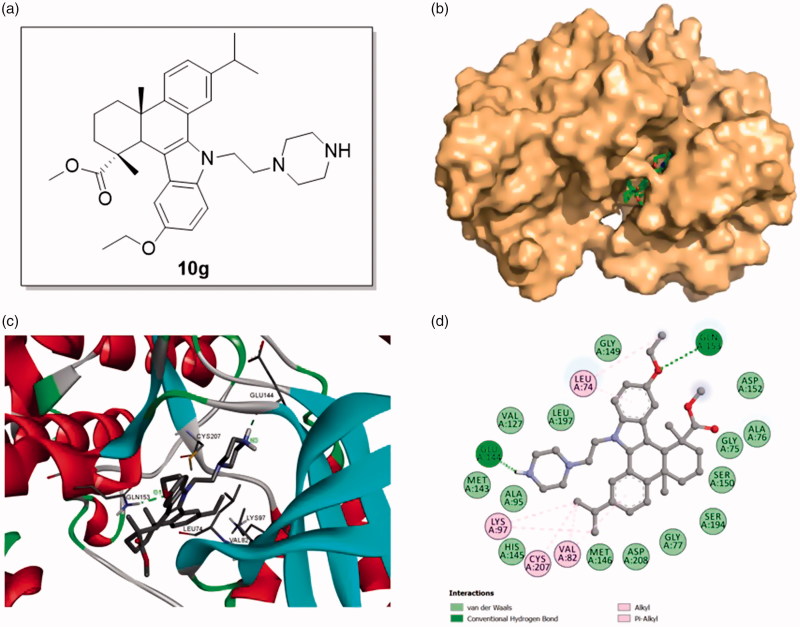
Binding mode of compound **10g** at MEK1 kinase domain (PDB: 3EQF). (a) Molecular structure of compound **10g**; (b) Space filling model of MEK1 protein with compound **10g** embedded in the binding pocket; (c) Binding pose of compound **10g** within the MEK1 kinase domain. Ligand and key residues are presented as stick models and colored by atom type, whereas the proteins are represented as ribbons. The dash lines exhibit the hydrogen bond interactions; (d) 2D projection drawing of compound **10g** docked into MEK1 active site.

To gain more understanding of the interaction between target compounds and MEK, we explored their binding modes by molecular docking based on the reported MEK-1/inhibitor complex structure (PDB code: 3EQF). The docking studies were performed by using GLIDE docking module of Schrodinger suite 2015–1 and the docking results were analyzed and visualized by Discovery Studio 2016 Client. The binding models of compound **10g** with MEK1 protein were shown in Figure 4.

It was observed that compound **10g** could be suitably docked into the binding site of MEK1 protein (Figure 4(b,c)), affording a significant docking score (–7.518), comparable to the docking score of AZD6244 (–7.401). Specifically, the (piperazin-1-yl)ethyl side chain was deeply inserted into the binding pocket of MEK1 structure, and a hydrogen bond was established between N3 atom of **10g** and Glu 144 (N3–H⋅⋅⋅O/Glu 144, angle N – H⋅⋅⋅O = 125.1°, distance = 1.90 Å). On the other hand, the ethoxyl group on the indole benzene ring also played an important role in the interaction. The ethoxyl group formed a hydrogen bond with Gln 153 (O1⋅⋅⋅H – N/Gln 153, angle N – H⋅⋅⋅O = 150.0°, distance = 2.08 Å) and an alkyl hydrophobic interaction with Leu 74. Other alkyl hydrophobic interactions were also detected between the isopropyl group on C12 and Lys 97, Cys 207, Val 82 and π-alkyl hydrophobic interactions formed between two benzene rings and Val 82, Leu74. In addition, the molecule also formed van der Waals interactions with residues Met 146, His 145, Ala 95, Met 143, Val 127, Leu 197, Gly 149, Asp 152, Ala 76, Gly 75, Ser 150, Ser 194, Gly 77 and Asp 208 (Figure 4(d)). Taken together, the molecular docking results in combination with the biological assay data indicated that compound **10g** could be a promising MEK inhibitor worthy of further investigation.

### 3.5. Cell cycle analysis

**Figure 5.**
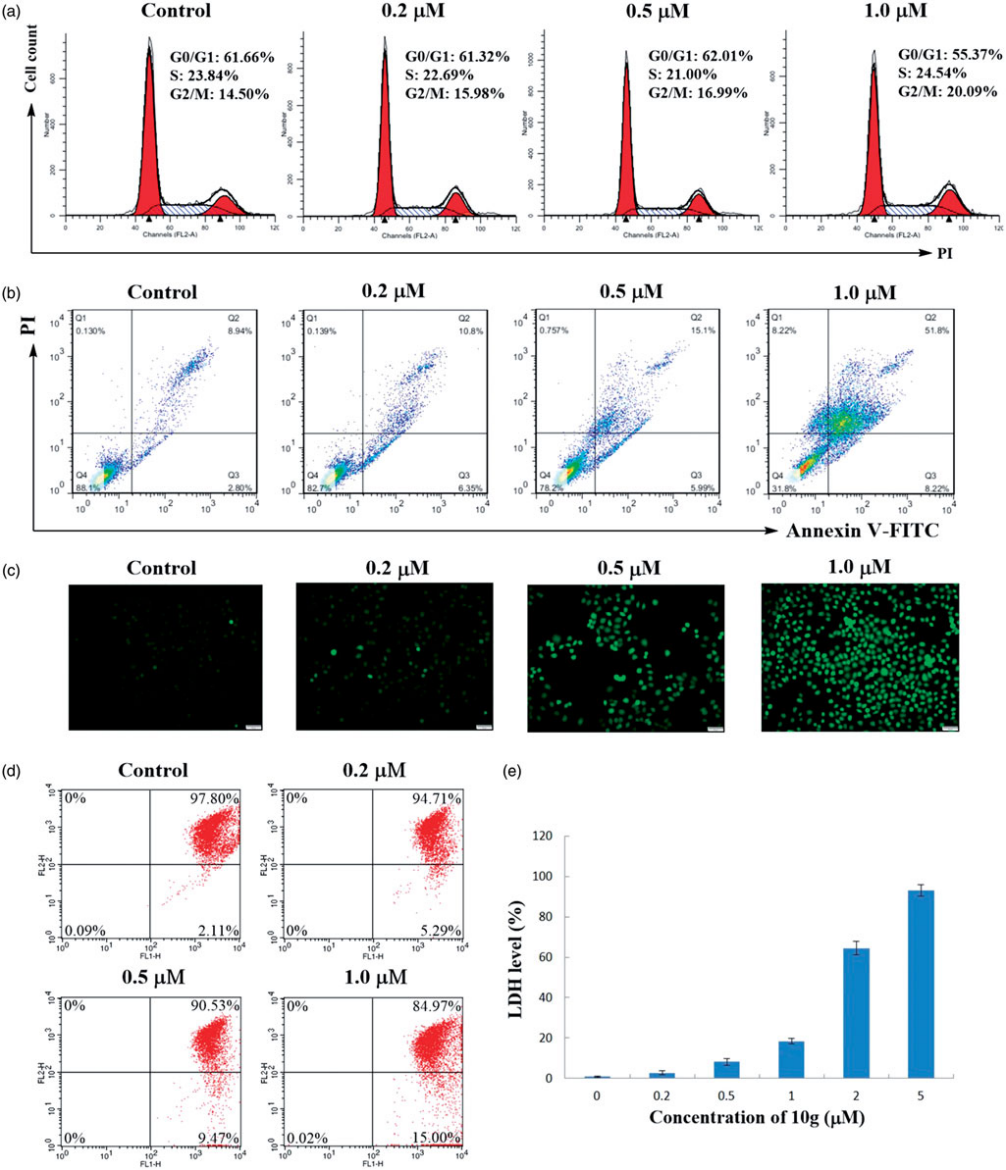
(a) Cell cycle assay. HepG2 cells were treated with different concentrations of compound **10g** (0, 0.2, 0.5, 1.0 μM) for 48 h, stained with propidium iodide (PI) and analysed using flow cytometer. (b) Annexin V-FITC/PI dual staining assay. HepG2 cells were treated with different concentrations of compound **10g** (0, 0.2, 0.5, 1.0 μM) for 48 h, stained with Annexin V-FITC/PI and analysed for apoptosis using flow cytometer. (c) ROS generation assay. HepG2 cells were treated with different concentrations of compound **10g** (0, 0.2, 0.5, 1.0 μM) for 48 h, stained with DCFH-DA and analysed using flow cytometer. (d) Mitochondrial membrane potential assay. HeLa cells were treated with compound **4d** (0, 0.2, 0.5, 1.0 μM) for 24 h, incubated with JC-1 and analysed using flow cytometry. (e) LDH release assay of HepG2 cells treated with different concentrations of compound **10g** (0, 0.2, 0.5, 1, 2 and 5 μM).

To determine whether the inhibition of cancer cell growth by compound **10g** was correlated with cell cycle arrest, HepG2 cells were treated with different concentrations of compound **10g** (0, 0.2, 0.5 and 1.0 µM) for 48 h. After staining with propidium iodide (PI), the cell cycle distribution of the treated cells was analysed by flow cytometry method. As shown in Figure 5(a), the percentage of cells in G2/M phase gradually increased from 14.50% (0 µM) to 20.09% (2 µM), while the G0/G1 phase cells decreased from 61.66% (0 µM) to 55.37% (2 µM). These results indicated that compound **10g** could dose-dependently arrest the cell cycle of HepG2 cells at G2/M phase.

### 3.6. Annexin V-FITC/PI dual staining assay

In order to investigate whether compound **10g** could induce apoptosis, HepG2 cells were treated with different concentrations of compound **10g** (0, 0.2, 0.5 and 1.0 µM) for 48 h. Then the treated cells were subjected to Annexin V-FITC/PI dual staining followed by flow cytometry assay. As shown in Figure 5(b), the percentage of early and late apoptotic cells (lower right quadrant, AV+/PI– and upper right quadrant, AV+/PI+, respectively) significantly increased from 11.74% (0 µM) to 60.02 (2 µM). The results suggested that compound **10g** could induce the cell death of HepG2 cells in a dose-dependent manner.

### 3.7. ROS generation assay

Reactive oxygen species (ROS) are chemically reactive chemical species containing oxygen, which can exert oxidative stress to cells and result in severe damage to organelles. Excessive ROS generation renders cells vulnerable to apoptosis^41^. To determine whether compound **10g** could trigger ROS generation in HepG2 cells to induce cell death, the cells were treated with different concentrations of compound **10g** for 48 h, and the ROS generation was assayed using the fluorescent probe 2,7-dichlorofluorescein diacetate (DCF-DA) by fluorescence microscopy. As shown in Figure 5(c), the treated cells exhibited significant green fluorescence in a dose-dependent manner, indicating that compound **10g** could remarkably induce ROS generation in HepG2 cells.

### 3.8. Mitochondrial membrane potential (MMP) assay

It has been widely believed that ROS accumulation could decrease mitochondrial membrane potential (ΔΨ_m_) and promote apoptosis. The disruption of mitochondrial function is considered as one of the most important apoptotic pathways, which has been recognized as an attractive antitumour target^42^. To investigate the correlation between MMP and cell death induced by compound **10g**, the measurement of MMP were carried out by JC-1 assay kit on the instructions of the manual. As shown in Figure 5(d), the percentage of cells with low ΔΨ_m_ (Lower right quadrant) increased from 2.11% (Control) to 5.29% (0.2 µM), 9.47% (0.5 µM) and 15.00% (1.0 µM), which implied that compound **10g** could result in the decrease of mitochondrial membrane potential in a concentration dependent manner, and thus the mitochondrial apoptotic pathway was probably involved in the cell death induced by the title compound.

### 3.9. Lactate dehydrogenase (LDH) leakage assay

As an enzyme existing in cytoplasm, LDH will be released into the medium when the cell membrane integrity is destructed. Hence, the destroyed cell membrane can be confirmed by LDH leakage assay^37^. HepG2 cells were incubated with different concentrations of compound **10g** for 24 h, and then the extent of LDH leakage was detected using a LDH assay kit as the description of the manual. As shown in Figure 5(e), the relative LDH leakage level increased significantly from 0.80% (Control) to 93.03% (5 µM) in a dose-dependent manner. The results indicated that the treatment of compound **10g** would markedly destroy the cell membrane integrity of HepG2 cells.

### 3.10. Cell death inhibition assay

**Figure 6.**
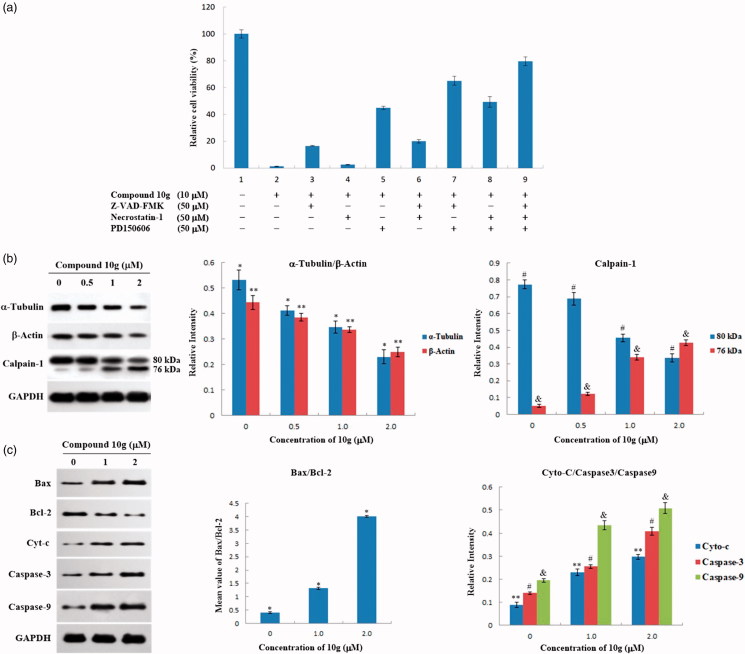
Compound **10g** induced oncosis and apoptosis of HepG2 cells. (a) PD150606 and Z-VAD-FMK partially reversed compound **10g**-induced cell death. HepG2 cells were incubated with PD150606, Z-VAD-FMK or Necrostatin-1 at indicated concentration for 24 h alone or in combination with compound **10g** at indicated concentration for 24 h. (b) Compound **10g** induced the degradation of α-tubulin and β-actin, and activated oncosis marker calpain-1 autolysis from the 80 kDa event to 76 kDa event in western blot analysis. *, *p* < 0.01; **, *p* < 0.01; #, *p* < 0.01; &, *p* < 0.001. (c) Compound **10g** induced the upregulation of apoptotic protein Bax, caspase-3, caspase-9 and plasmic cytochrome c levels, and the downregulation of antiapoptotic protein Bcl-2 level. *, *p* < 0.001; **, *p* < 0.01; #, *p* < 0.01; and *p* < 0.01.

After confirming the cytotoxic effect of compound **10g** on hepatocarcinoma cells, we sought to clarify the detailed type of cell death caused by **10g**. In this effort, necrosis inhibitor Necrostatin-1, apoptosis inhibitor Z-VAD-FMK and oncosis inhibitor PD150606 were utilised to reverse compound **10g**-inducing cell death. As shown in Figure 6(a), the relative cell viability of HepG2 cells treated with **10g** (10 µM) was only 1.22%. However, PD150606 (50 µM) could significantly reverse compound **10g**-induced cell death with the relative cell viability increased to 44.87%. Z-VAD-FMK could also increase the relative cell viability to 16.53%. On the contrary, Necrostatin-1 had no apparent effect on cell death caused by compound **10g**. Based on these results, we speculated that compound **10g** could induce cell death of HepG2 cells through oncosis and apoptosis.

### 3.11. Western blot analysis

To further explore whether compound **10g** could induce the oncosis and apoptosis of HepG2 cells, a number of key protein markers involved in oncosis and apoptosis pathway were examined through Western blot analysis. Because calpains were reported to function in the process of oncotic cell death^43^, we further detected the expression level of calpain-1 in compound **10g**-treated HepG2 cells. It has been reported that the activation of calpain at the membrane included the dissociation of calpain subunits and two successive autolytic events (80 kDa and 76 kDa)^44^, we found that calpain-1 autolysed from 80 kDa event to 76 kDa event in a dose-dependent manner when treated with compound **10g**, which might imply the activation of this protein during the oncotic cell death (Figure 6(b)). Moreover, excessively active calpain can break down molecules in the cytoskeleton, and α-tubulin and β-actin has been identified as calpain substrates during oncosis process^43^^,^^45^. Therefore, we evaluated the expression levels of α-tubulin and β-actin in compound **10g**-treated HepG2 cells. As shown in Figure 6(b), the level of α-tubulin and β-actin suffered a significant decrease in a dose-dependent manner. These results further indicated that compound **10g** could induce oncotic cell death in HepG2 cells.

In addition, because the apoptosis inhibitor Z-VAD-FMK could partially reverse cell death, the expression of several apoptosis-related proteins was also detected in compound **10g**-treated cells. Two important members of Bcl-2 family, the pro-apototic protein Bax and the anti-apoptotic protein Bcl-2 are key regulators of apoptosis^46^. With the dysfunction of mitochondrial membrane, cytochrome c is released to cytoplasma, which participates in the activation of downstream caspases. Caspases are a family of cysteinyl aspartate specific proteases involved in apoptosis, which can be divided into groups of initiators (caspase 8, 9 and 10) and executioners (caspase 3, 6 and 7)^47^^,^^48^. Therefore, the expression of cytochrome c, caspase-3 and caspase-9 were also detected. As shown in Figure 6(c), the ratio of Bax/Bcl-2 increased significantly with the treatment of compound **10g**. In addition, the level of cytochrome c, caspase-3 and caspase-9 also increased dose-dependently in compound **10g**-treated HepG2 cells. These data suggested that compound **10g** could also induce the apoptosis of HepG2 cells through mitochondrial signalling pathway.

## 4. Conclusion

A series of novel 1*H*-dibenzo[*a*,*c*]carbazole derivatives of dehydroabietic acid with different *N*-(piperazin-1-yl)alkyl side chains were designed, synthesised and evaluated for their *in vitro* antiproliferative activities against three human hepatocarcinoma cell lines (SMMC-7721, HepG2 and Hep3B) and a normal human hepatocyte cell line (QSG-7701). Several derivatives displayed considerable *in vitro* antiproliferative activities in MTT assay. Especially, compound **10g** exhibited the most potent inhibitory activity against all the cancer cell lines and significantly lower cytotoxicity to human normal hepatocyte cells QSG-7701. *In vitro* pharmacological studies demonstrated that compound **10g** could significantly inhibit MEK1 kinase activity and thus impede the MAPK signaling pathway. In addition, it could arrest cell cycle of HepG2 cells at G2/M phase, induce intracellular ROS generation, decrease mitochondrial membrane potential, destroy the membrane integrity and finally lead to the oncosis and apoptosis of HepG2 cells. All these results highlight the potential of this class of derivatives as promising candidates for the discovery of new targeted anticancer agents.

## Supplementary Material

Supplemental Material
