# Supplementary material for: Synthesis and biological evaluation of novel N-(piperazin-1-yl)alkyl-1H-dibenzo[a,c]carbazole derivatives of dehydroabietic acid as potential MEK inhibitors
Source: J Enzyme Inhib Med Chem. 2019 Aug 25;34(1):1544–61. doi: 10.1080/14756366.2019.1655407 (PMC6720511; doi:10.1080/14756366.2019.1655407)
Supplement: Supplemental Material [file IENZ_A_1655407_SM1952.pdf]

Supplementary material

Synthesis and biological evaluation of novel *N*-(piperazin-1-yl)alkyl-1*H*-dibenzo[*a*,*c*]carbazole derivatives of dehydroabietic acid as potential MEK inhibitors

Hao Chen, Chao Qiao, Ting-Ting Miao, A-Liang Li, Wen-Yan Wang, Wen Gu

Jiangsu Provincial Key Lab for the Chemistry and Utilization of Agro-forest Biomass, Jiangsu Key Lab of Biomass-based Green Fuels and Chemicals, Co-Inovation Center for Efficient Processing and Utilization of Forest Products, College of Chemical Engineering, Nanjing Forestry University, Nanjing 210037, P. R. China

Corresponding author.

Email address: njguwen@163.com (W. Gu)

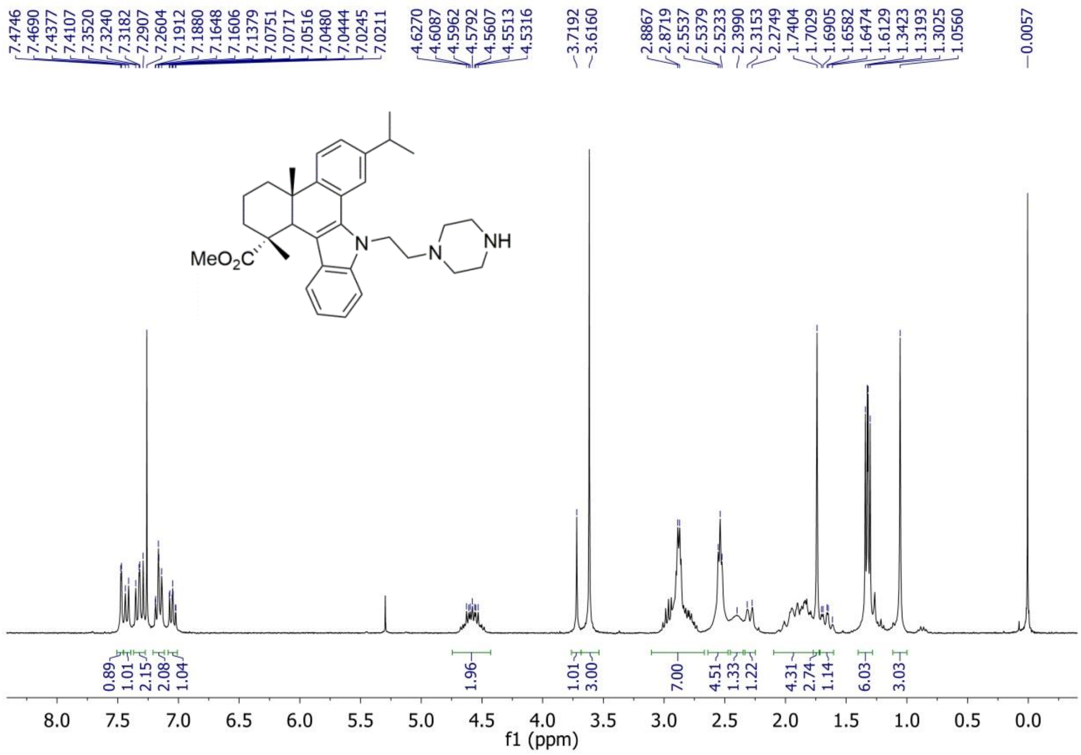

Fig. S1. ^{1}H-NMR spectrum of compound **6a** (300 MHz, $CDCl_3$)

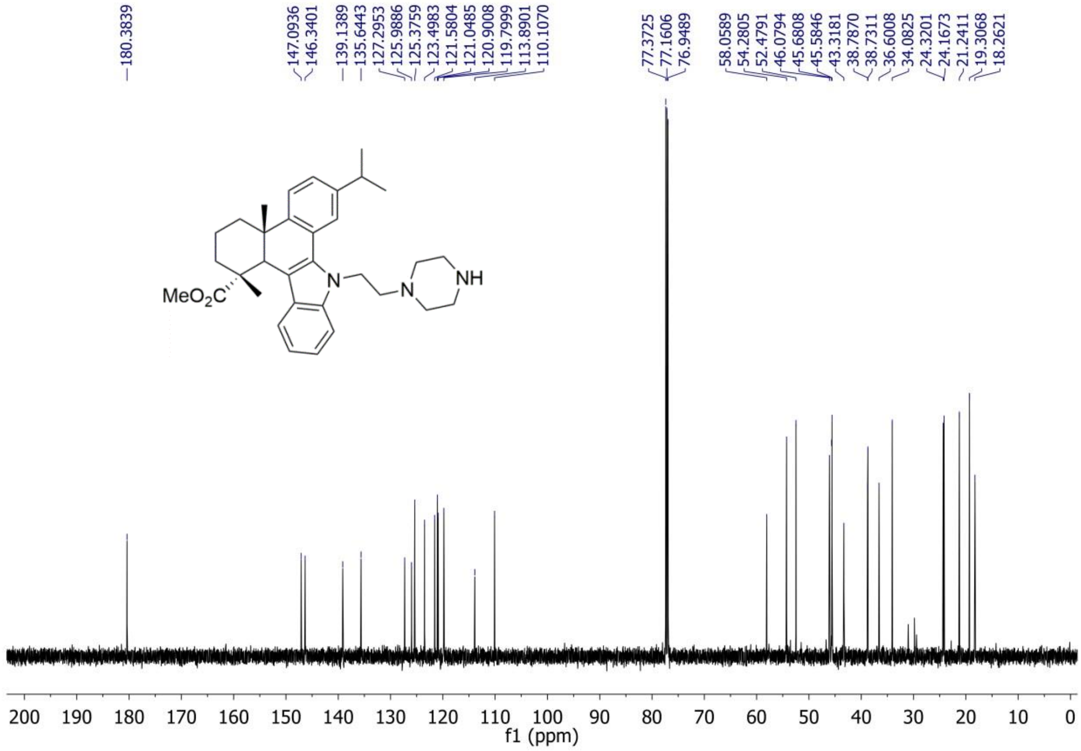

Fig. S2. ^{13}C-NMR spectrum of compound **6a** (150 MHz, $CDCl_3$)

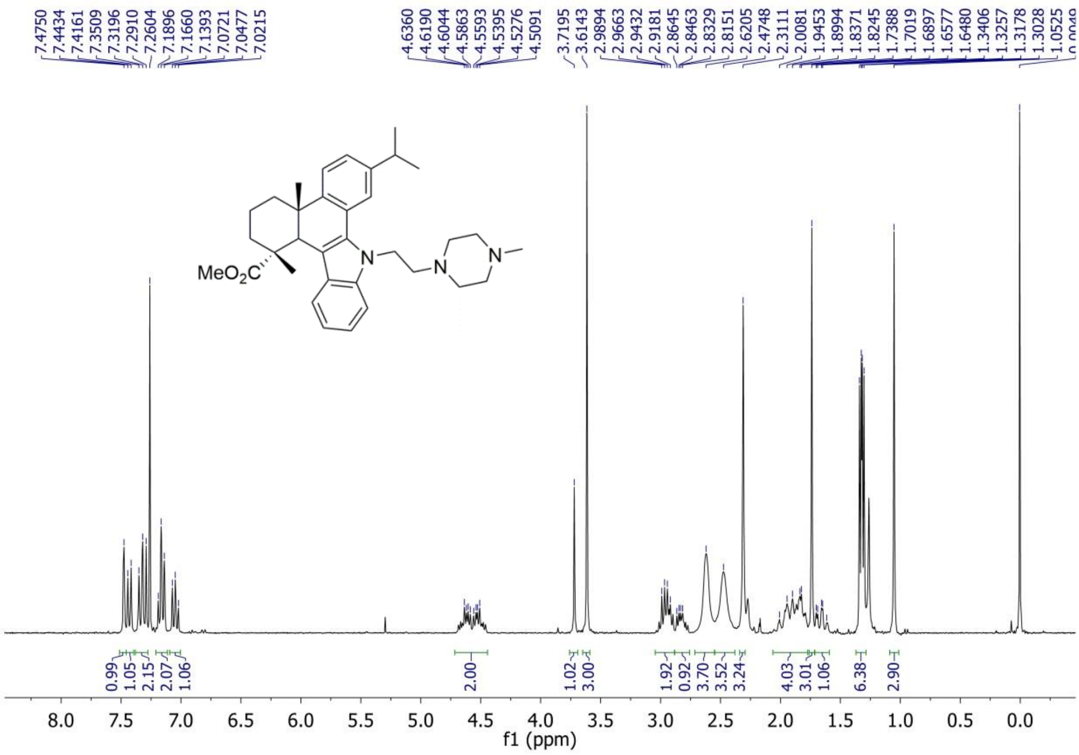

Fig. S3. ^{1}H-NMR spectrum of compound **6b** (300 MHz, $CDCl_3$)

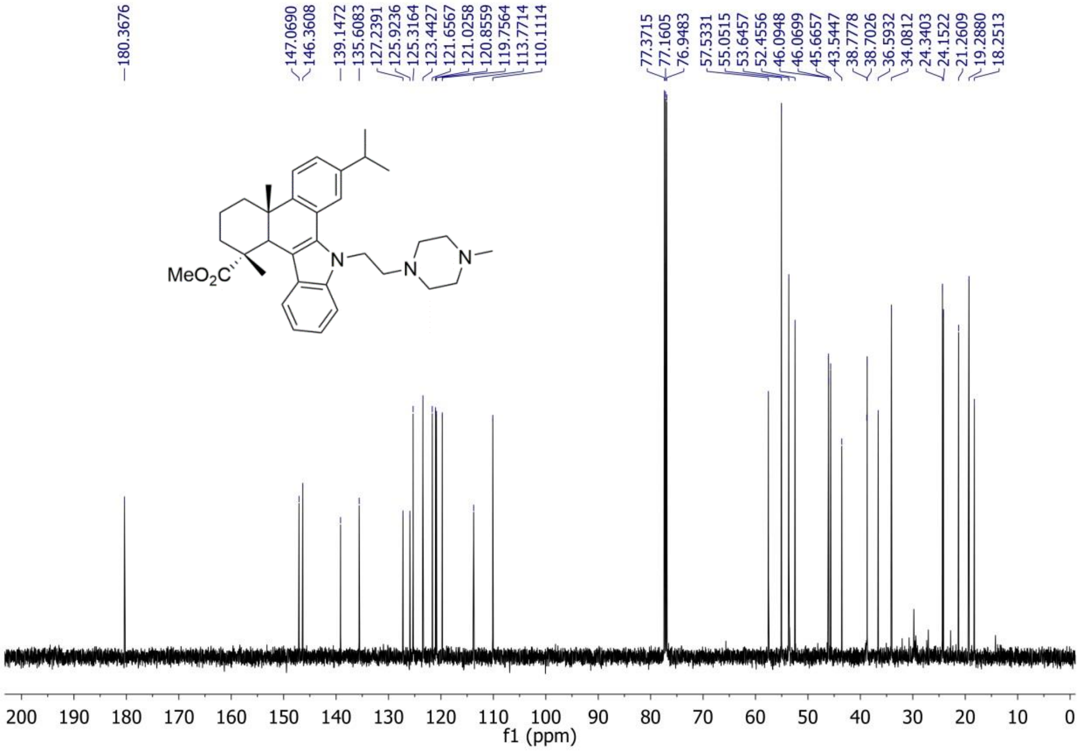

Fig. S4. ^{13}C-NMR spectrum of compound **6b** (150 MHz, $CDCl_3$)

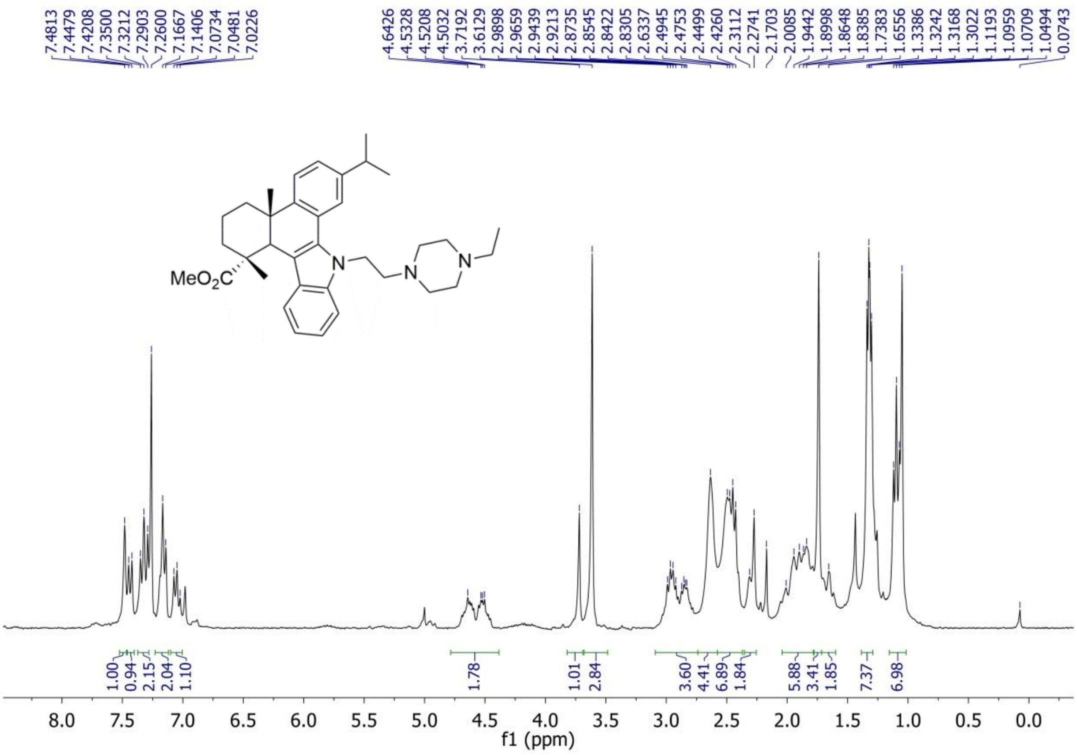

Fig. S5. ^{1}H-NMR spectrum of compound **6c** (300 MHz, $CDCl_3$)

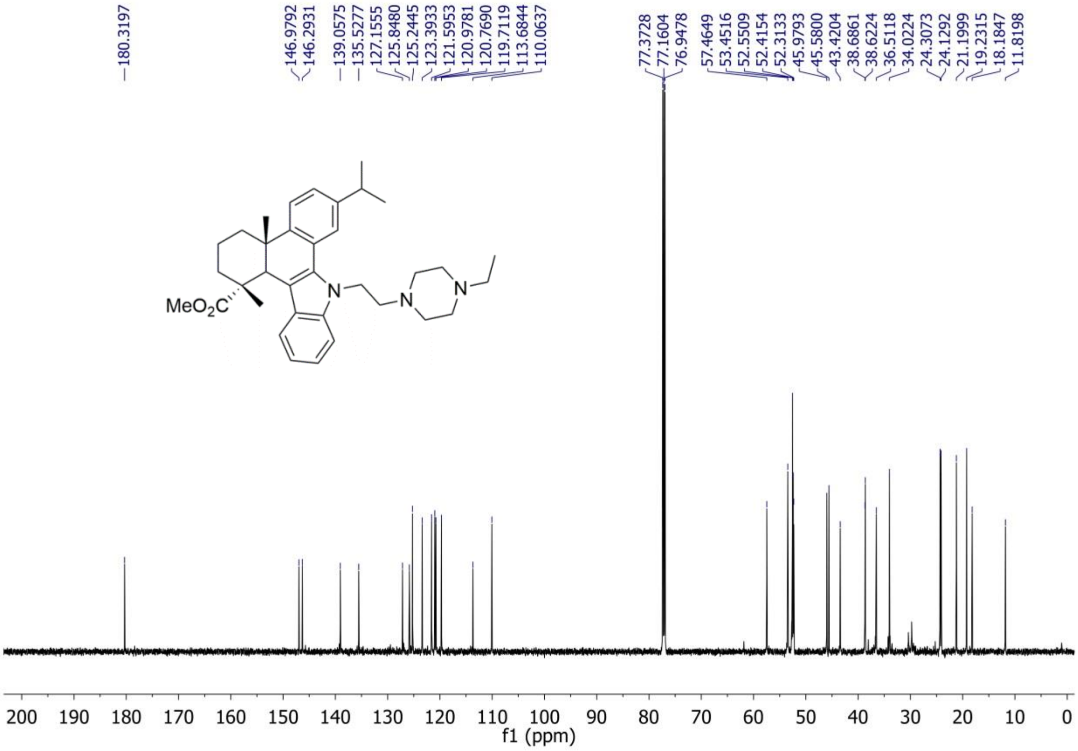

Fig. S6. ^{13}C-NMR spectrum of compound **6c** (150 MHz, $CDCl_3$)

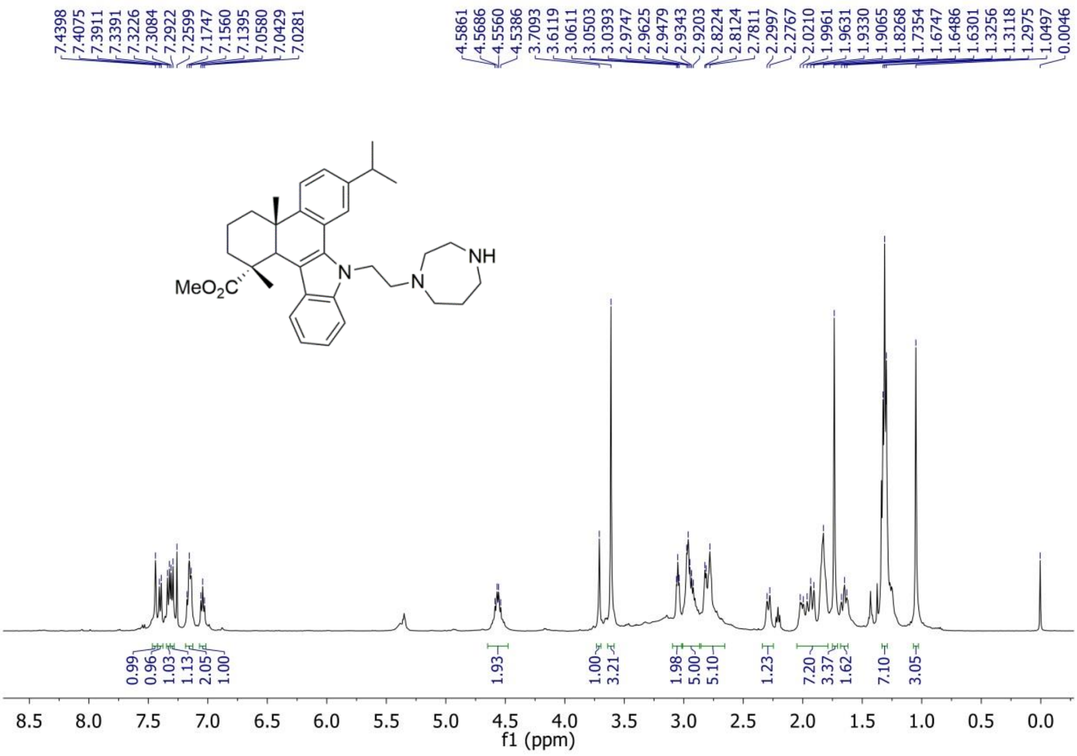

Fig. S7. ^{1}H-NMR spectrum of compound **6d** (500 MHz, $CDCl_3$)

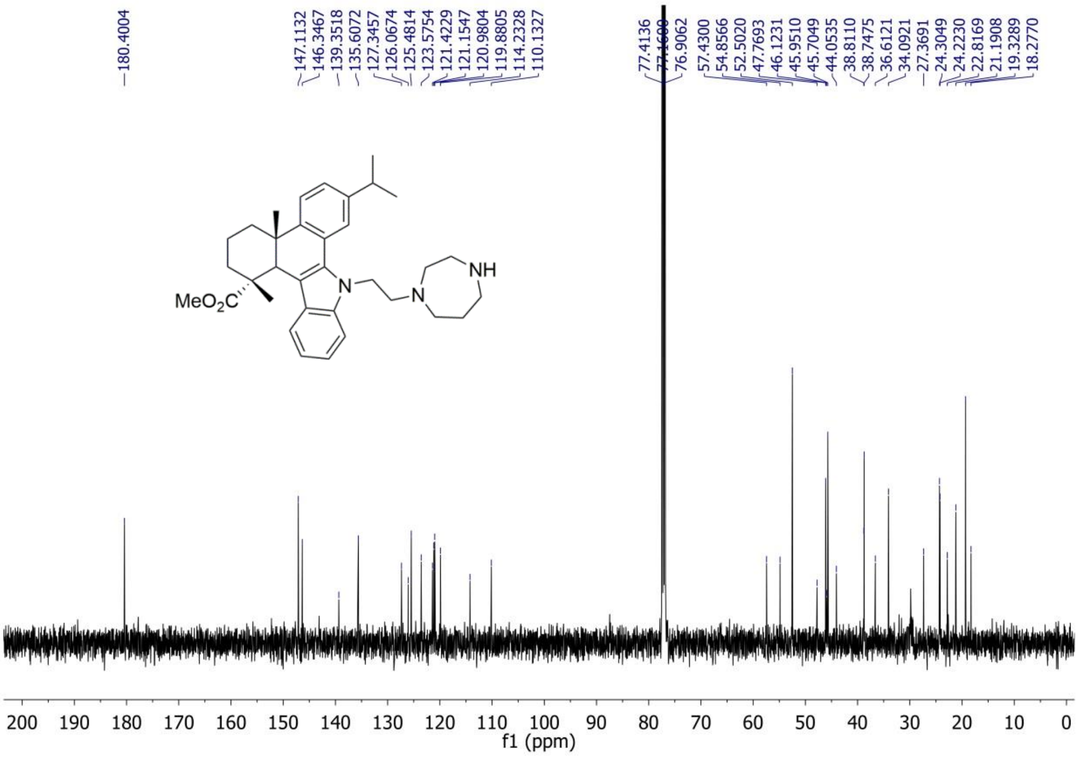

Fig. S8. ^{13}C-NMR spectrum of compound **6d** (125 MHz, $CDCl_3$)

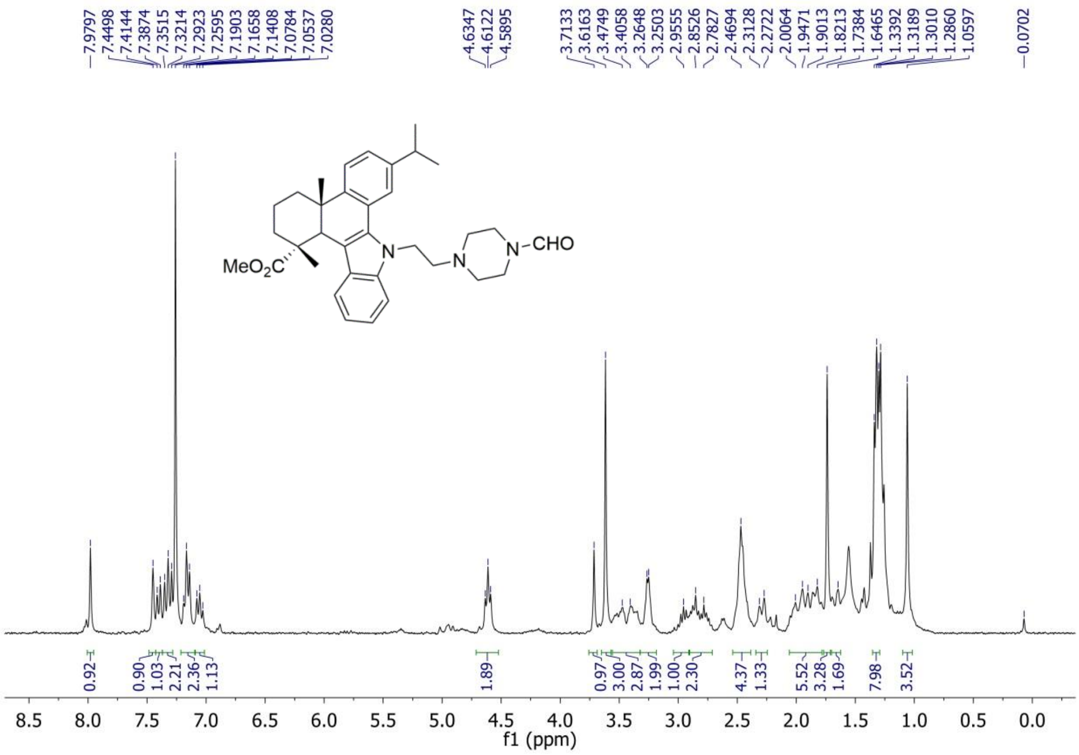

Fig. S9. ^{1}H-NMR spectrum of compound **6e** (300 MHz, $CDCl_3$)

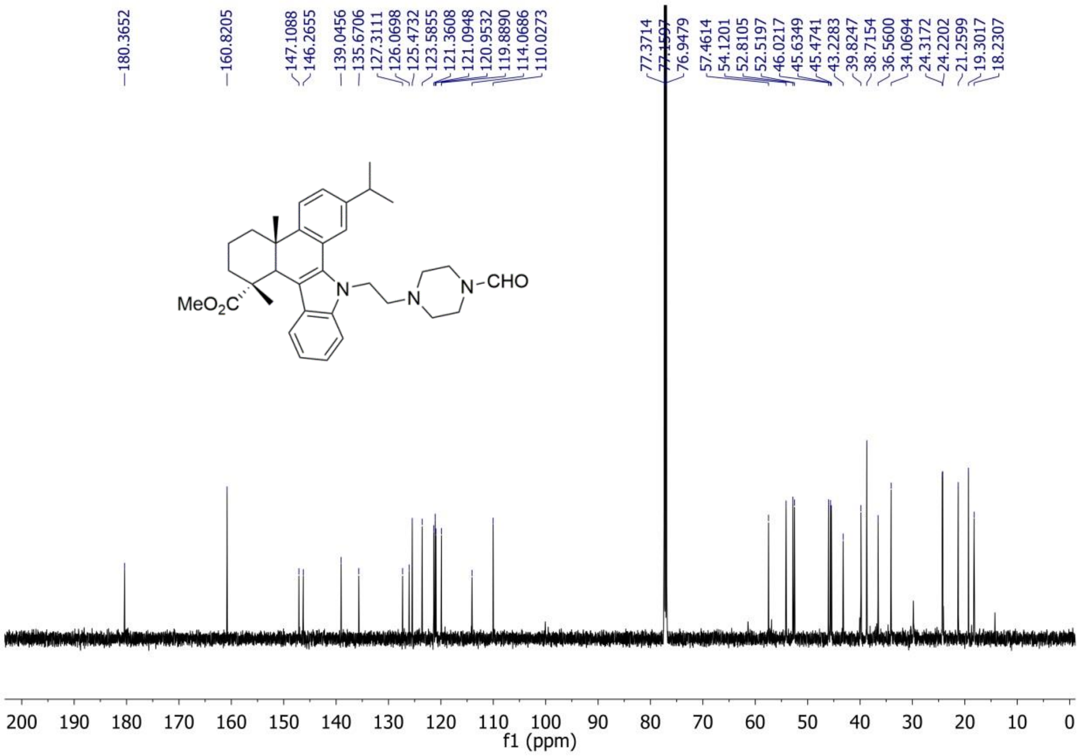

Fig. S10. ^{13}C-NMR spectrum of compound **6e** (150 MHz, $CDCl_3$)

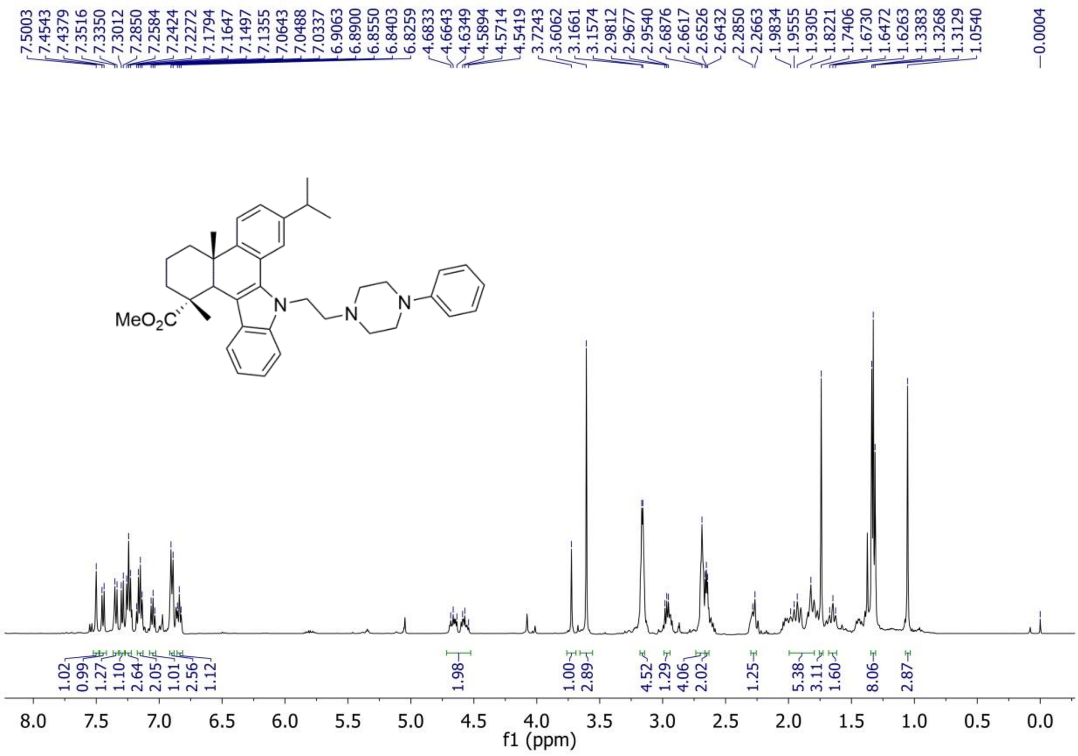

Fig. S11. ^{1}H-NMR spectrum of compound **6f** (500 MHz, $CDCl_3$)

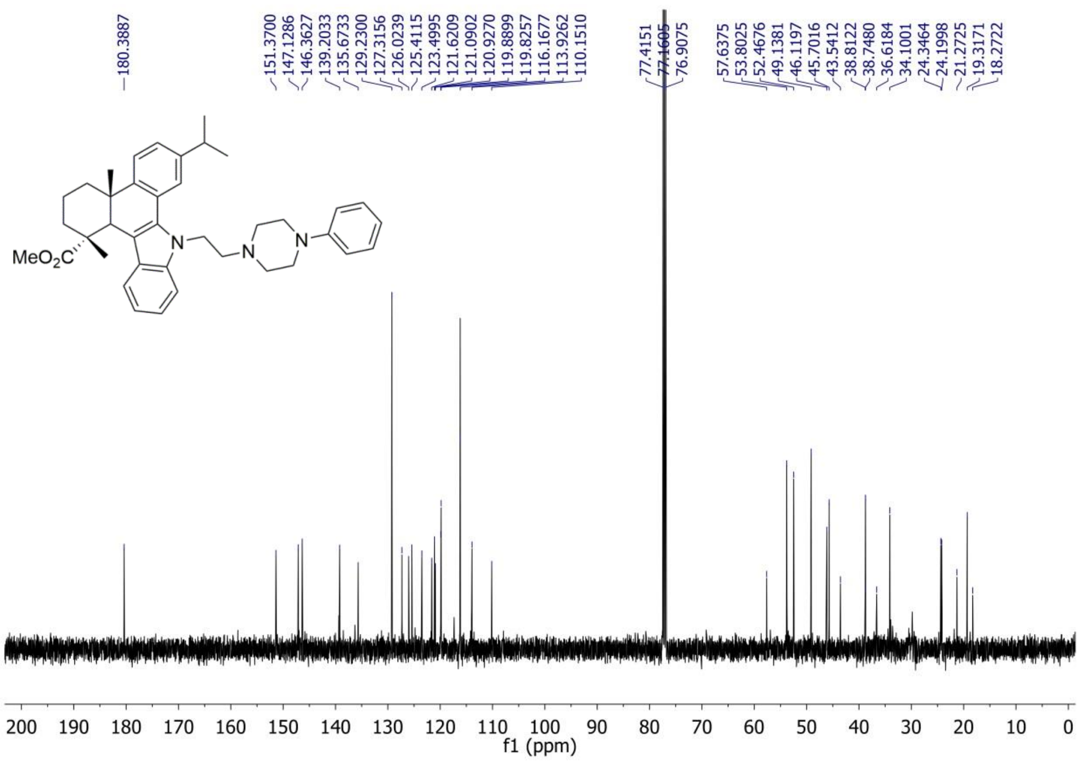

Fig. S12. ^{13}C-NMR spectrum of compound **6f** (125 MHz, $CDCl_3$)

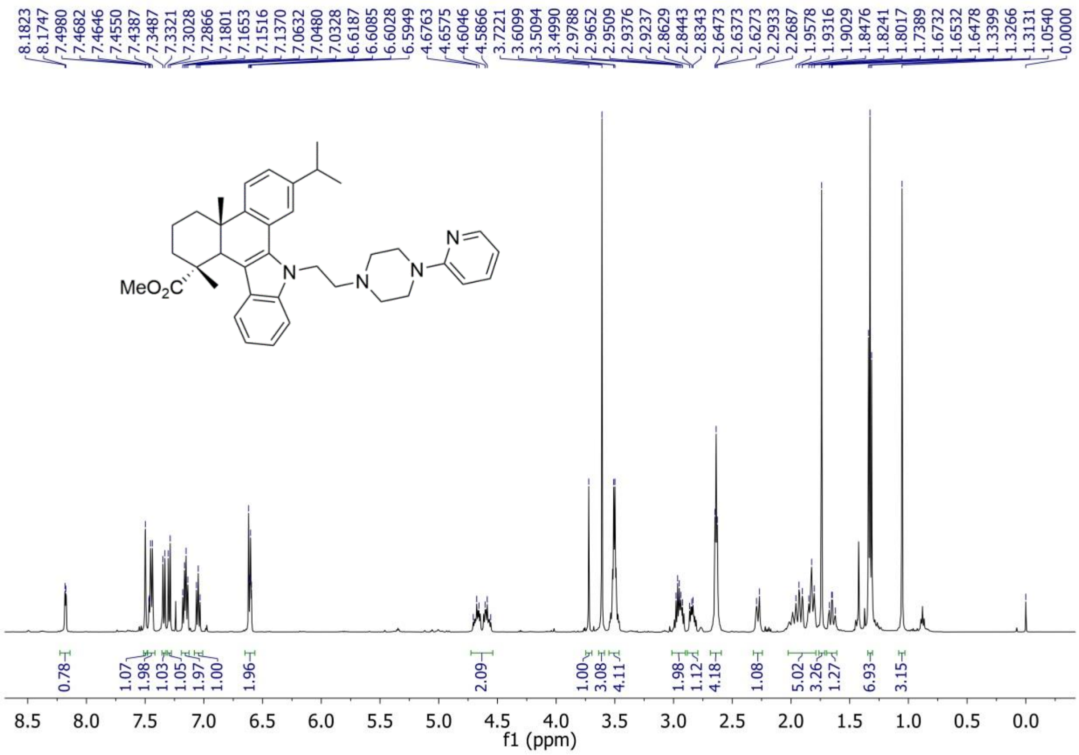

Fig. S13. ^{1}H-NMR spectrum of compound **6g** (500 MHz, $CDCl_3$)

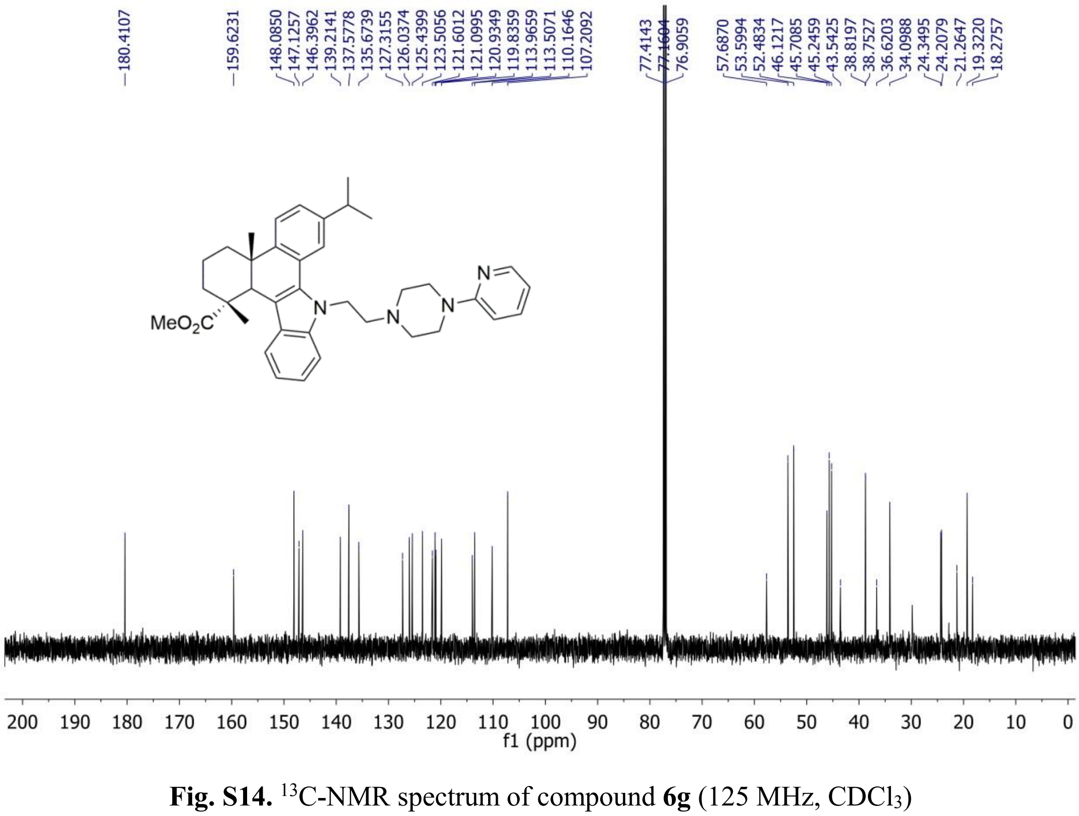

Fig. S14. ^{13}C-NMR spectrum of compound **6g** (125 MHz, $CDCl_3$)

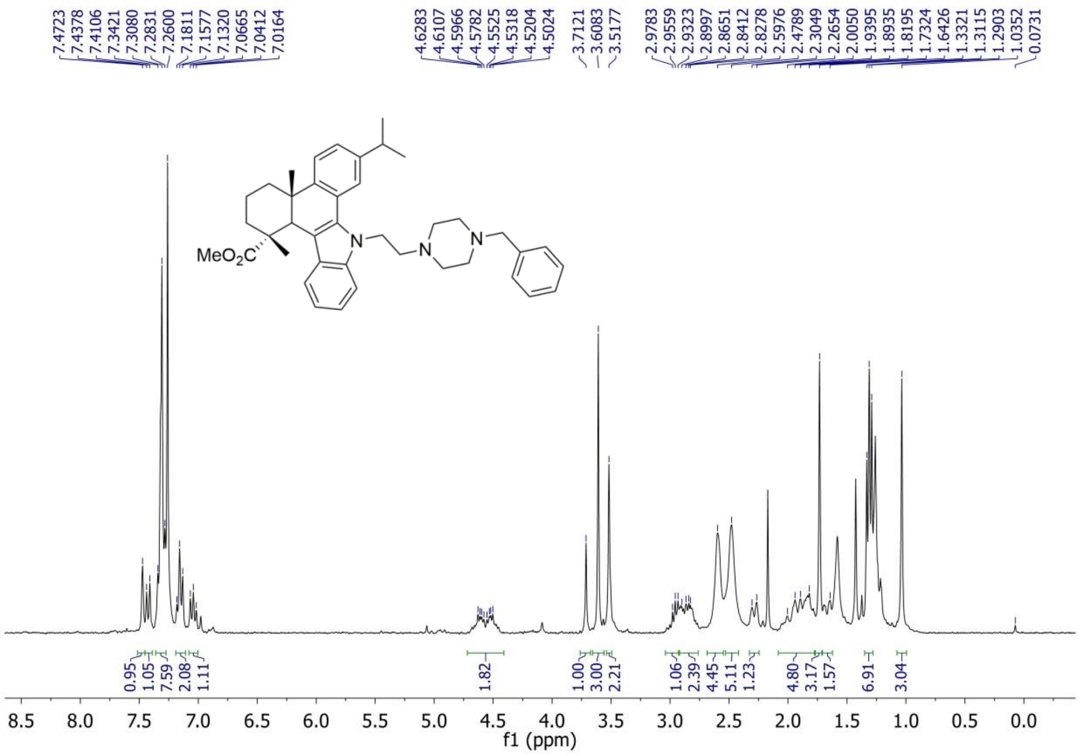

Fig. S15. ^{1}H-NMR spectrum of compound **6h** (300 MHz, $CDCl_3$)

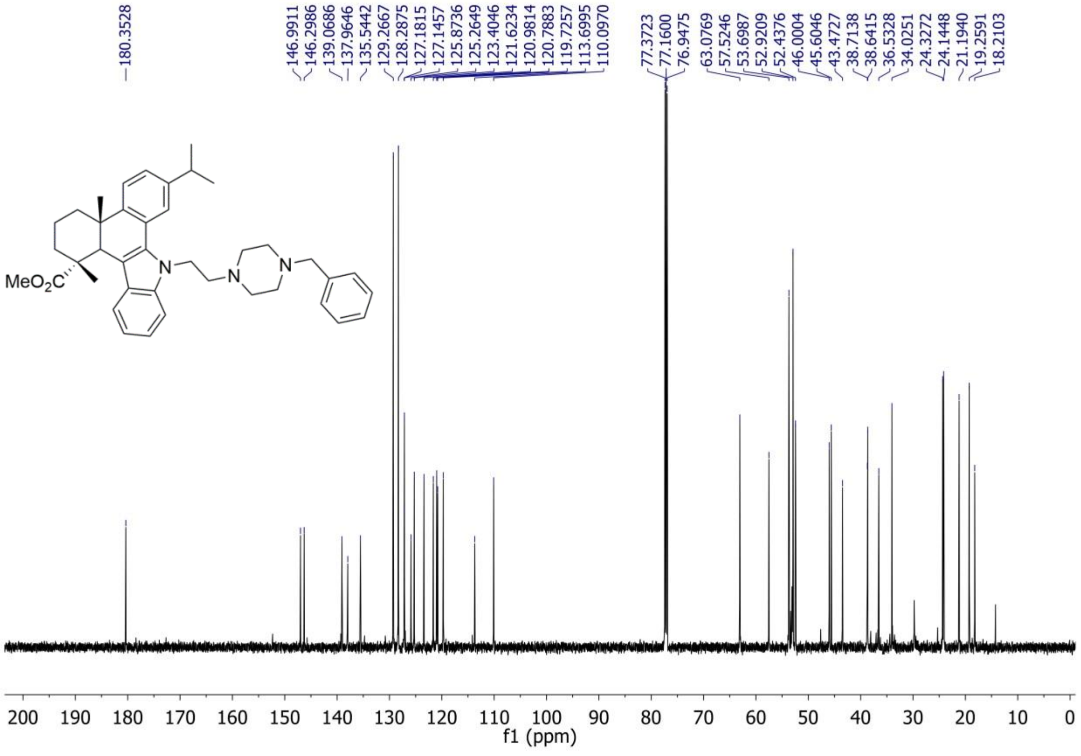

Fig. S16. ^{13}C-NMR spectrum of compound **6h** (150 MHz, $CDCl_3$)

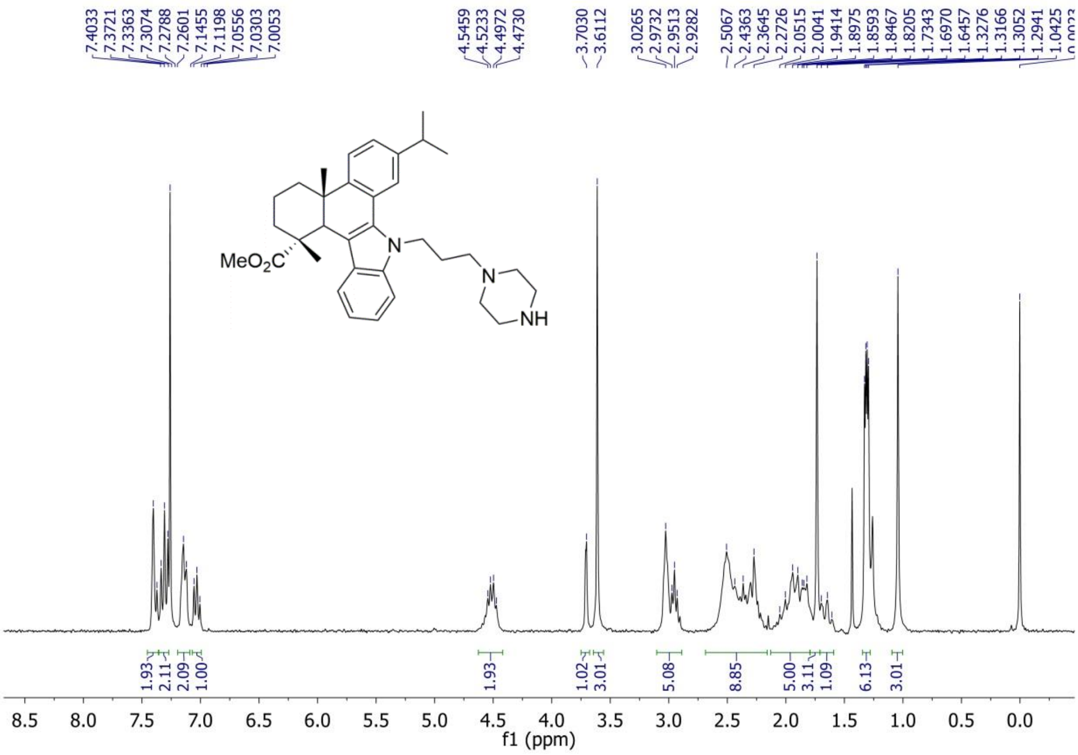

Fig. S17. ^{1}H-NMR spectrum of compound **7a** (300 MHz, $CDCl_3$)

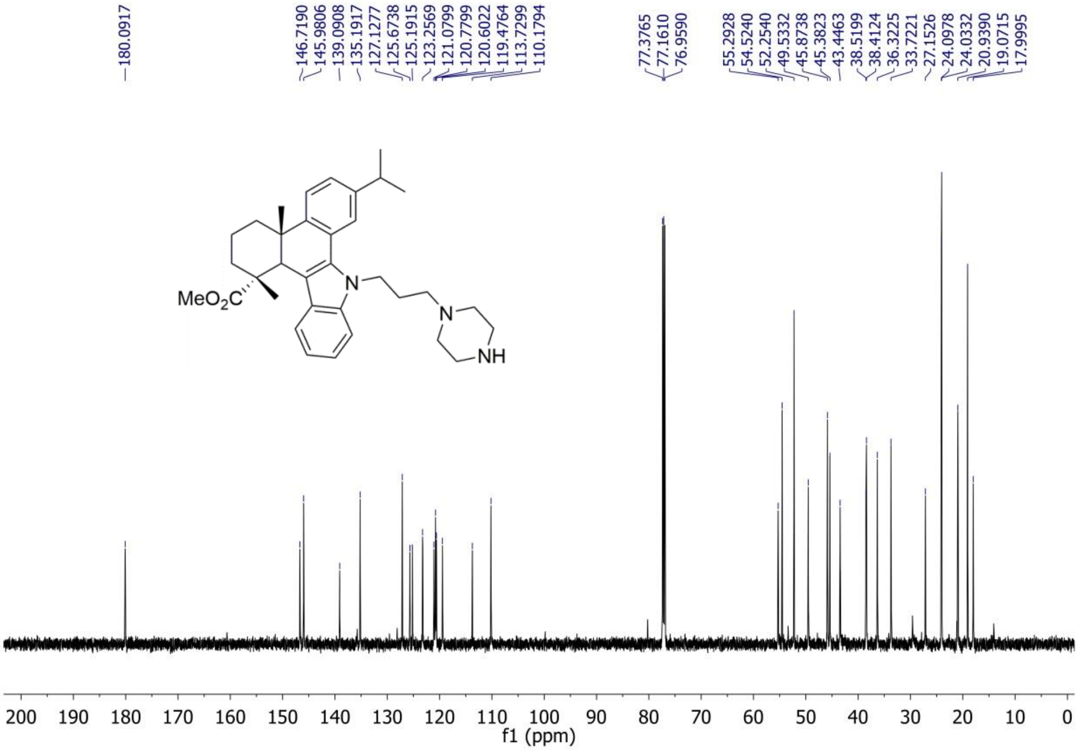

Fig. S18. ^{13}C-NMR spectrum of compound **7a** (150 MHz, $CDCl_3$)

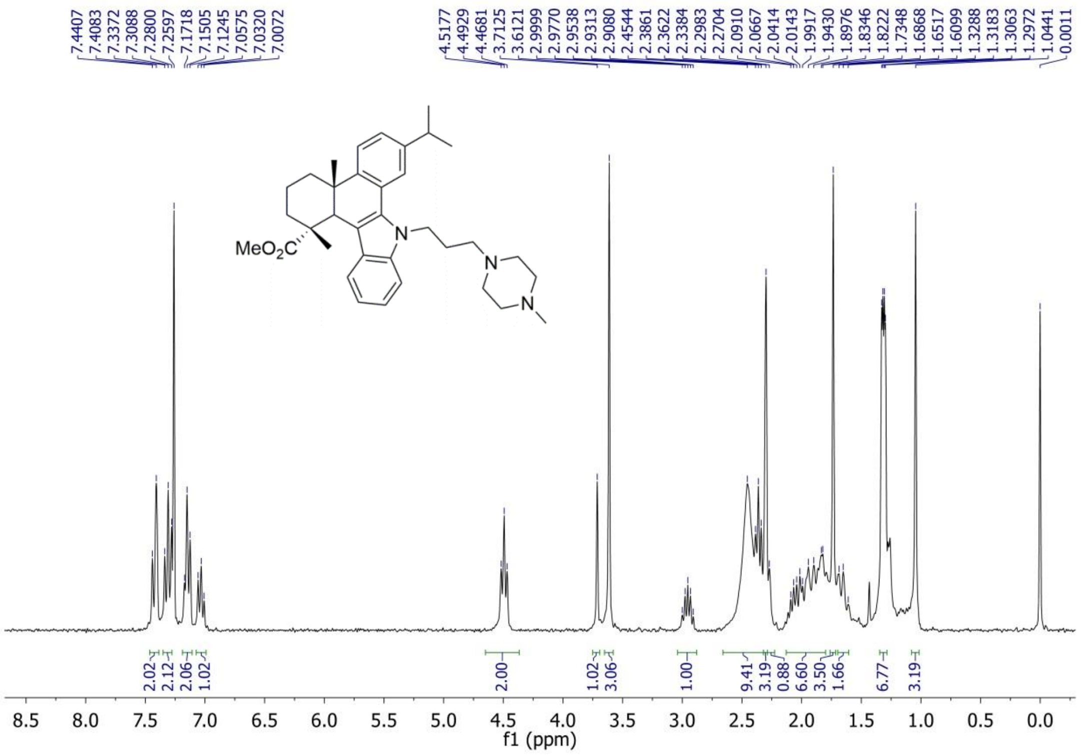

Fig. S19. ^{1}H-NMR spectrum of compound **7b** (300 MHz, $CDCl_3$)

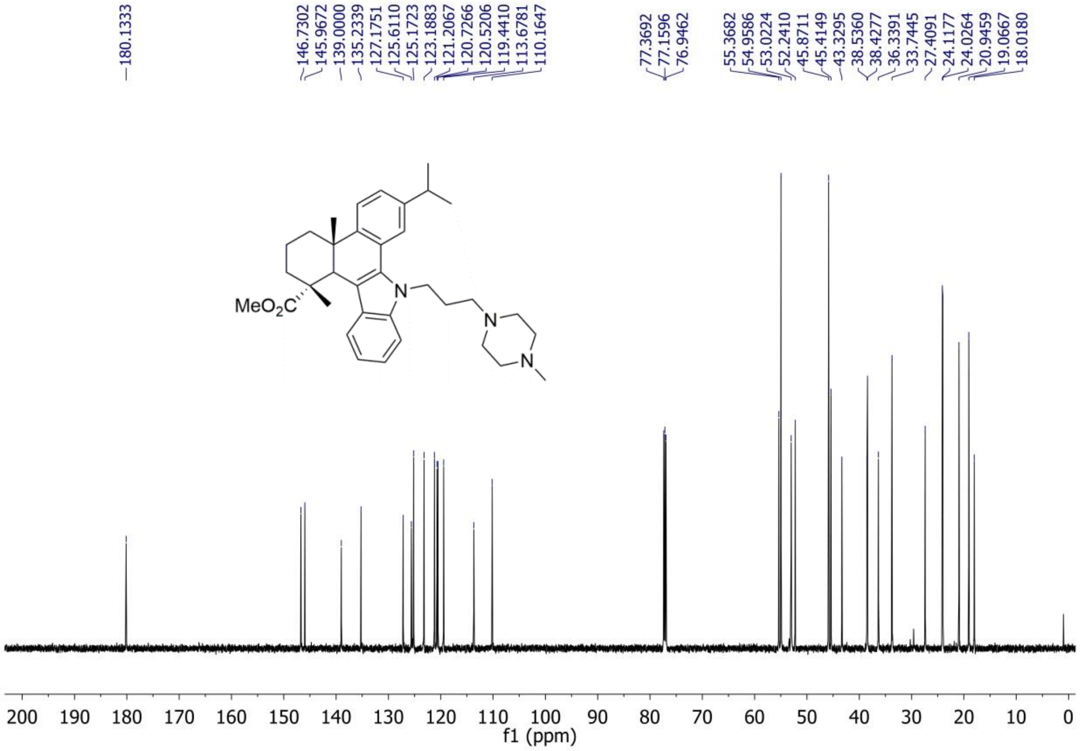

Fig. S20. ^{13}C-NMR spectrum of compound **7b** (150 MHz, $CDCl_3$)

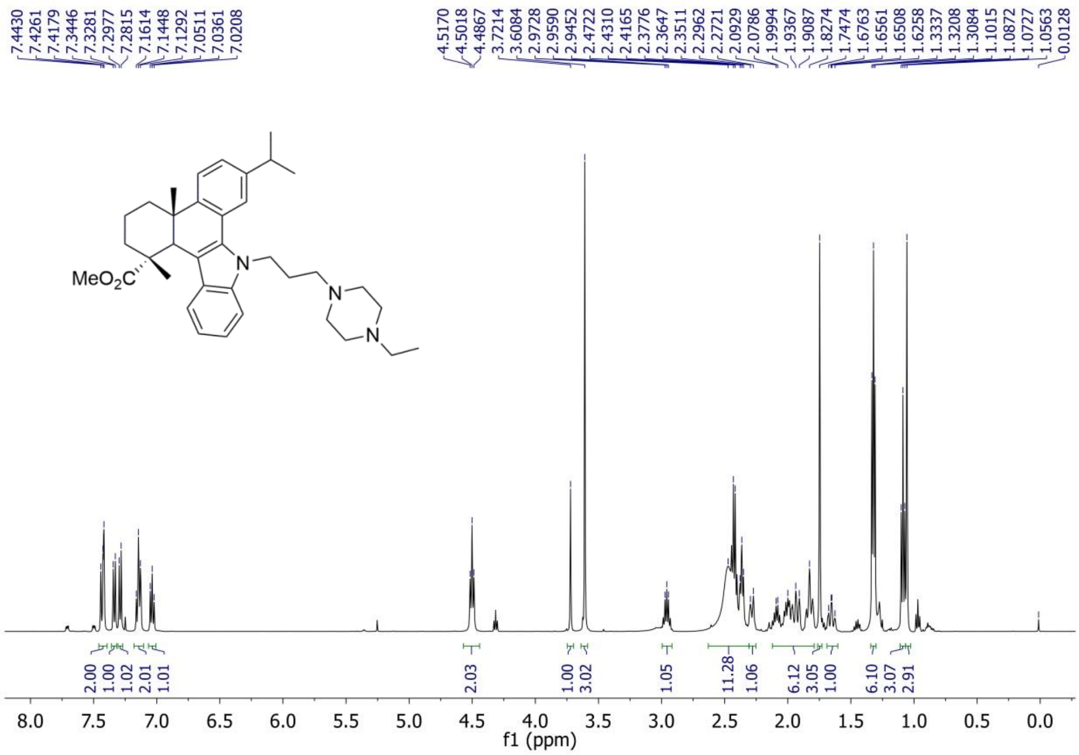

Fig. S21. ^{1}H-NMR spectrum of compound **7c** (500 MHz, $CDCl_3$)

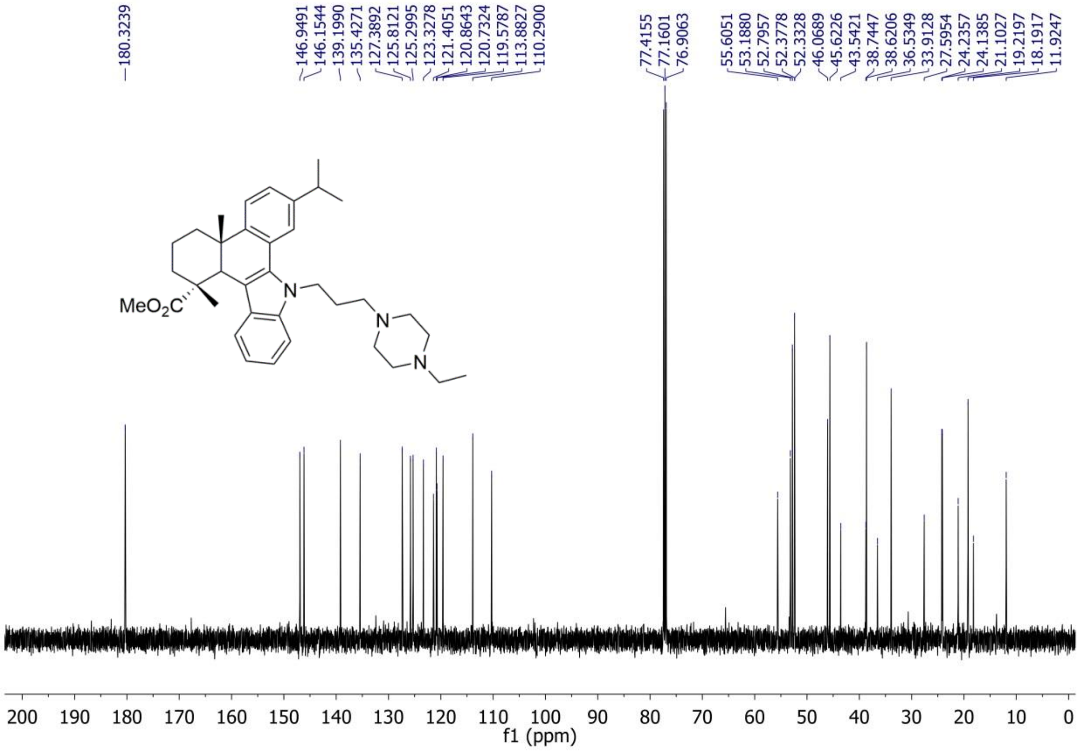

Fig. S22. ^{13}C-NMR spectrum of compound **7c** (125 MHz, $CDCl_3$)

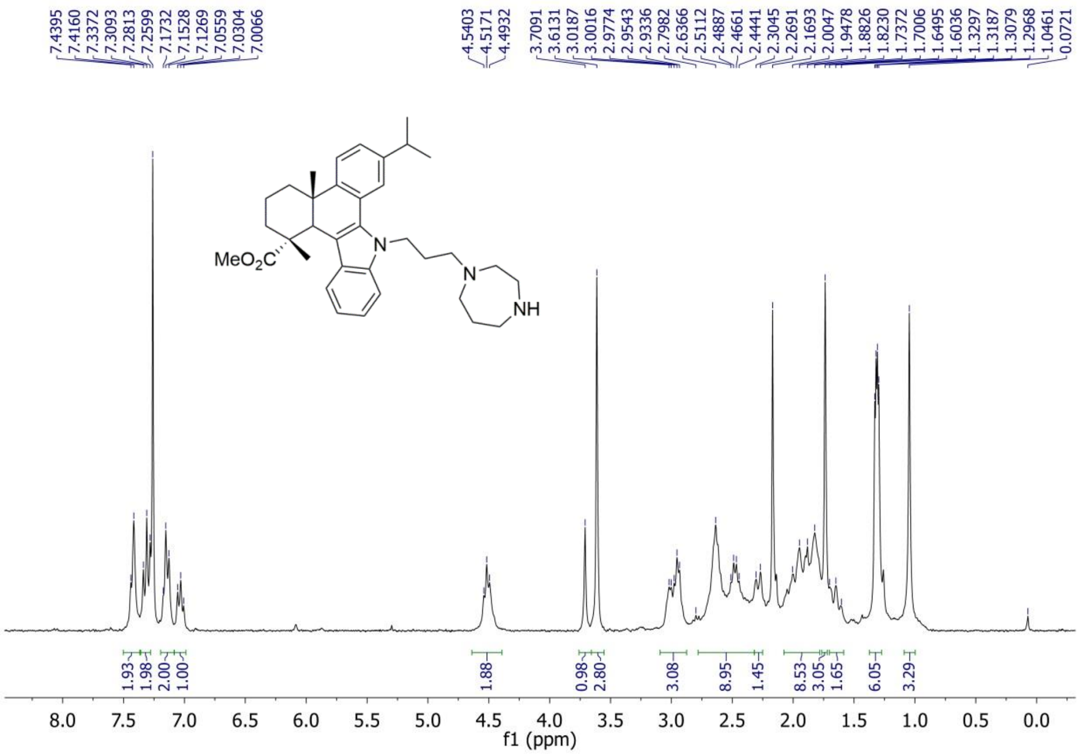

Fig. S23. ^{1}H-NMR spectrum of compound **7d** (300 MHz, $CDCl_3$)

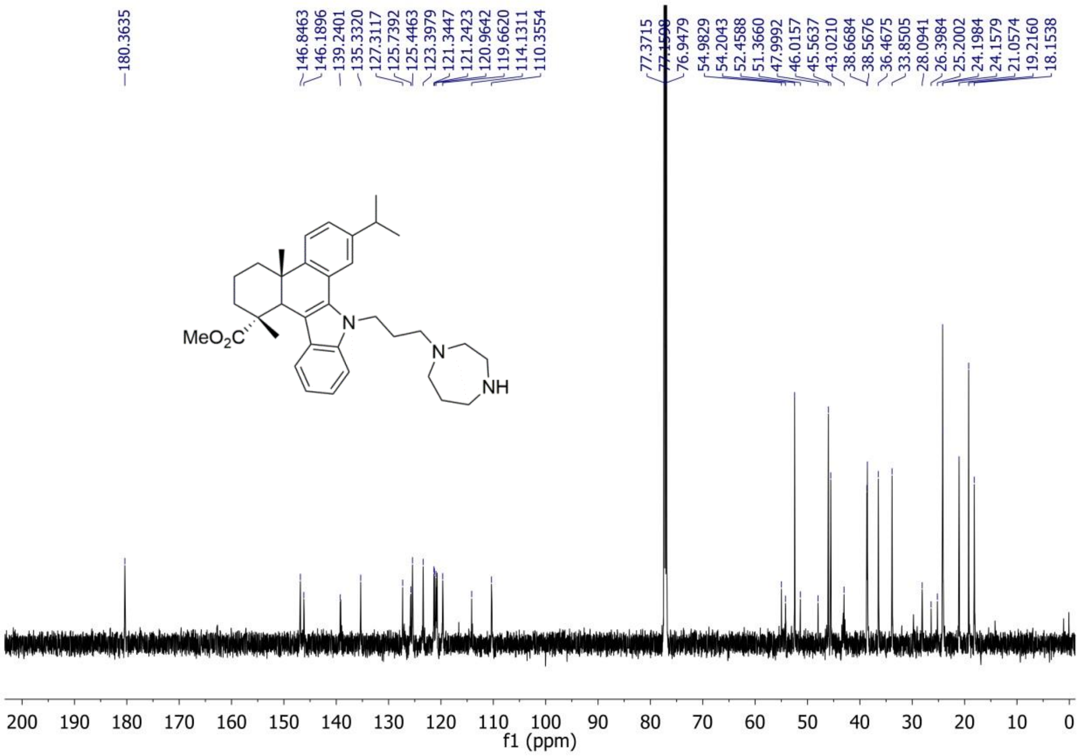

Fig. S24. ^{13}C-NMR spectrum of compound **7d** (150 MHz, $CDCl_3$)

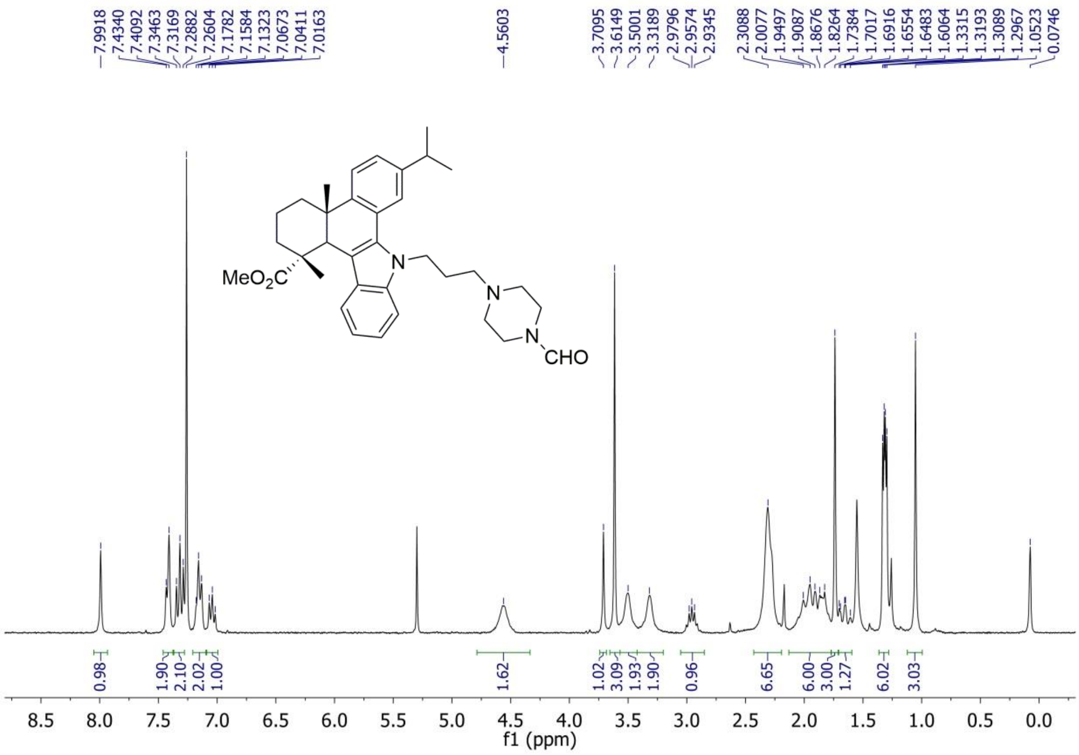

Fig. S25. ^{1}H-NMR spectrum of compound **7e** (300 MHz, $CDCl_3$)

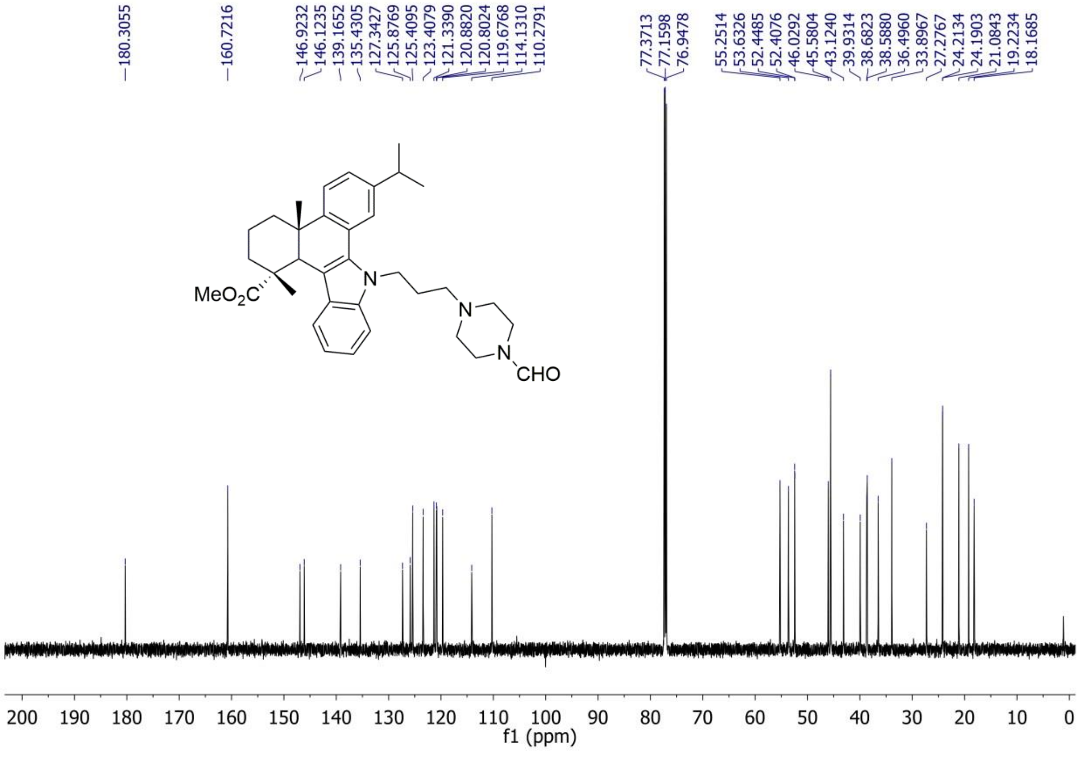

Fig. S26. ^{13}C-NMR spectrum of compound **7e** (150 MHz, $CDCl_3$)

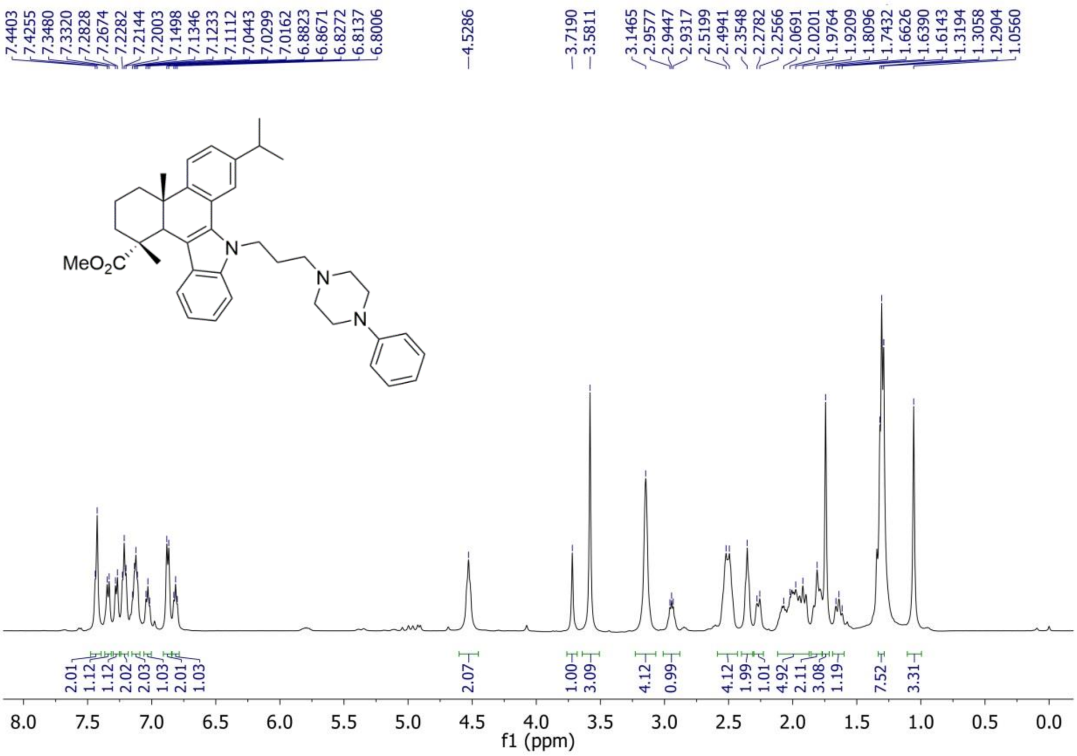

Fig. S27. ^{1}H-NMR spectrum of compound **7f** (500 MHz, $CDCl_3$)

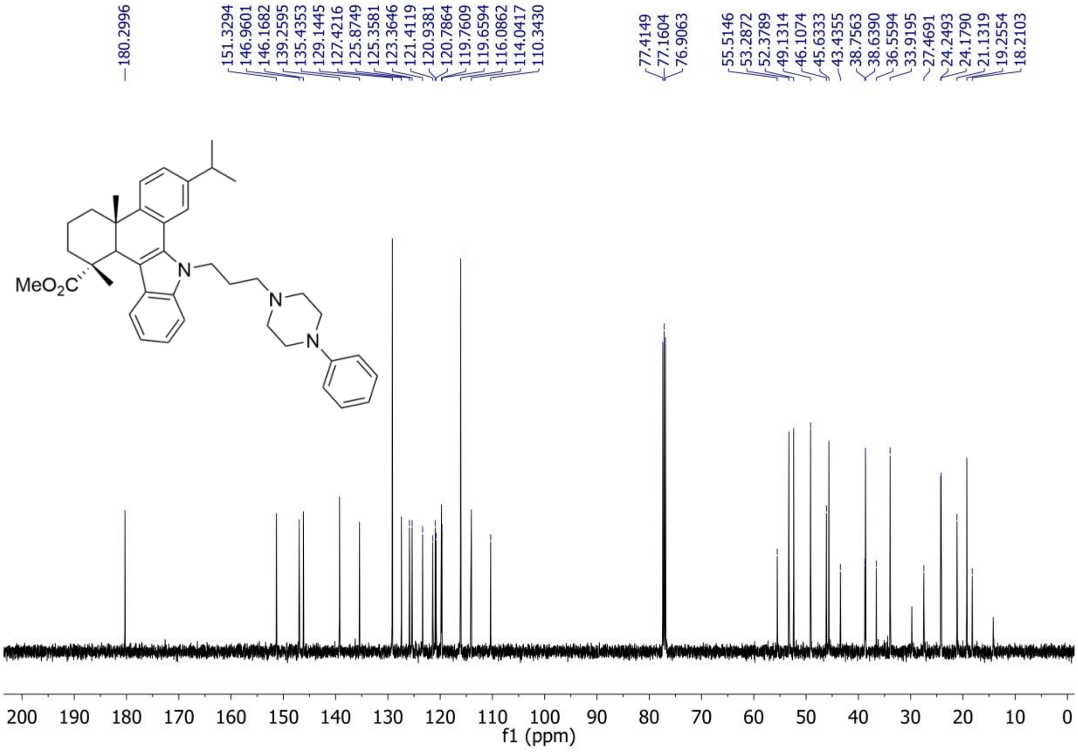

Fig. S28. ^{13}C-NMR spectrum of compound **7f** (125 MHz, $CDCl_3$)

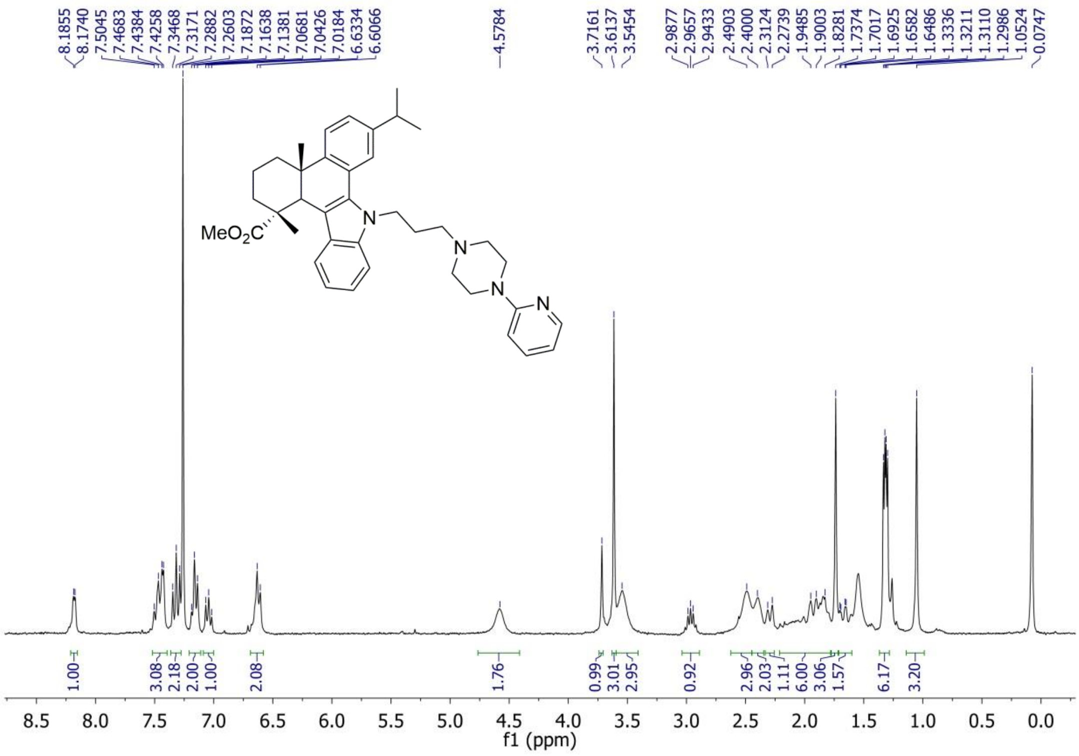

Fig. S29. ^{1}H-NMR spectrum of compound **7g** (300 MHz, $CDCl_3$)

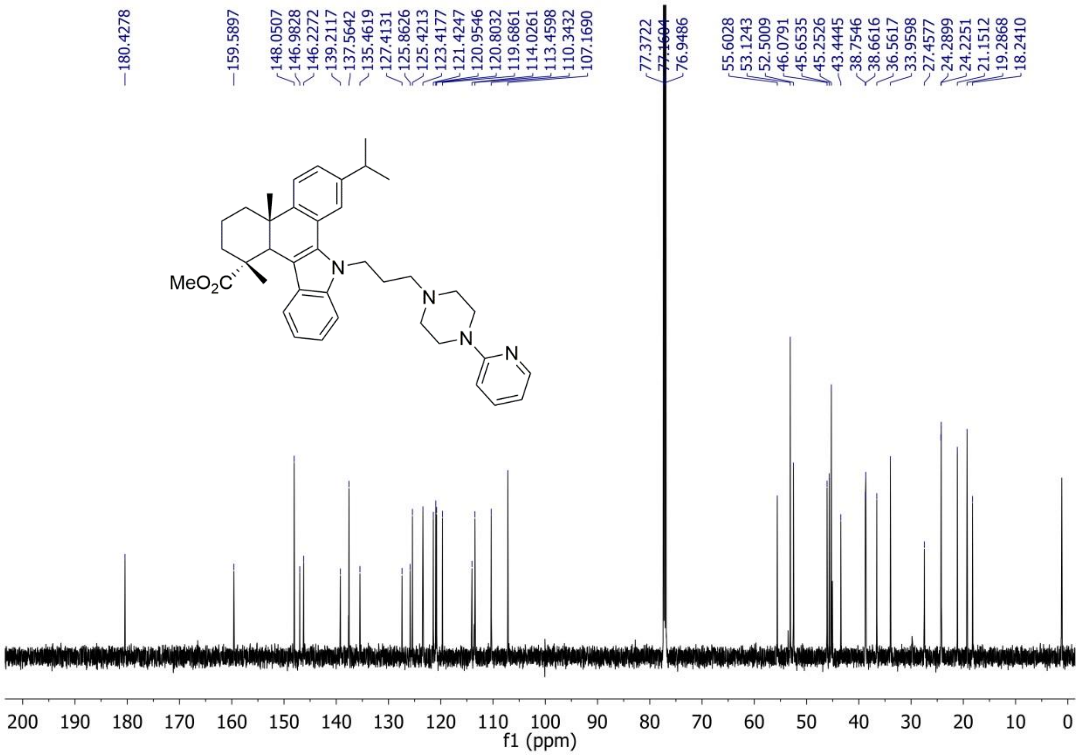

Fig. S30. ^{13}C-NMR spectrum of compound **7g** (150 MHz, $CDCl_3$)

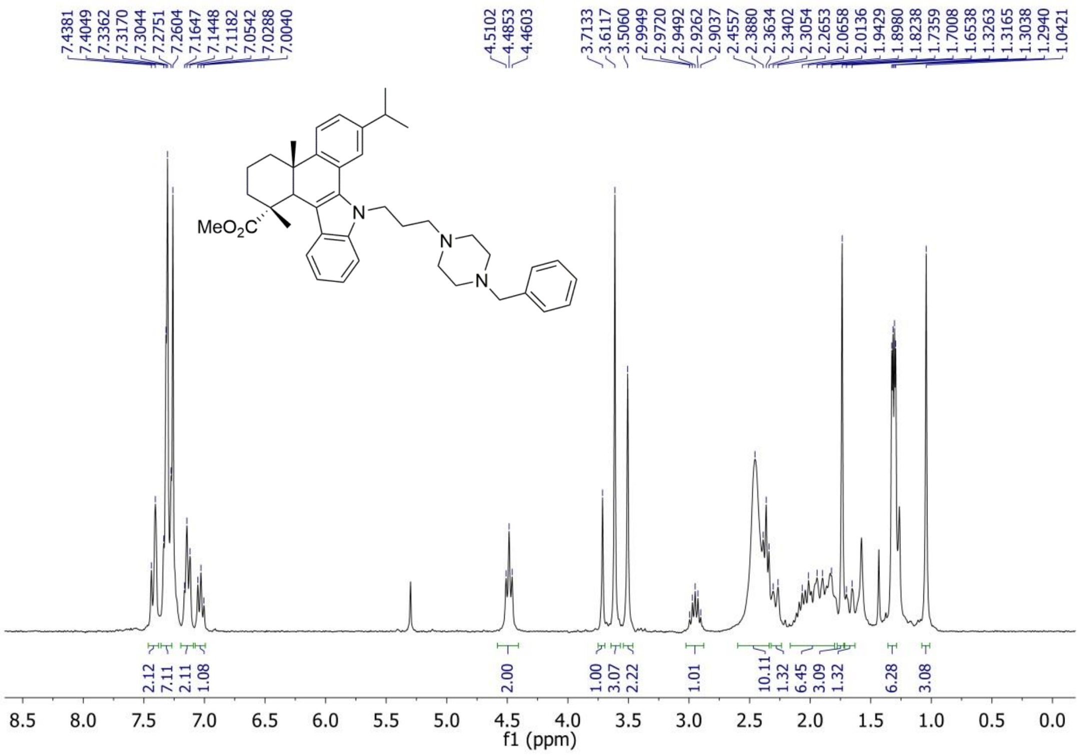

Fig. S31. ^{1}H-NMR spectrum of compound **7h** (300 MHz, $CDCl_3$)

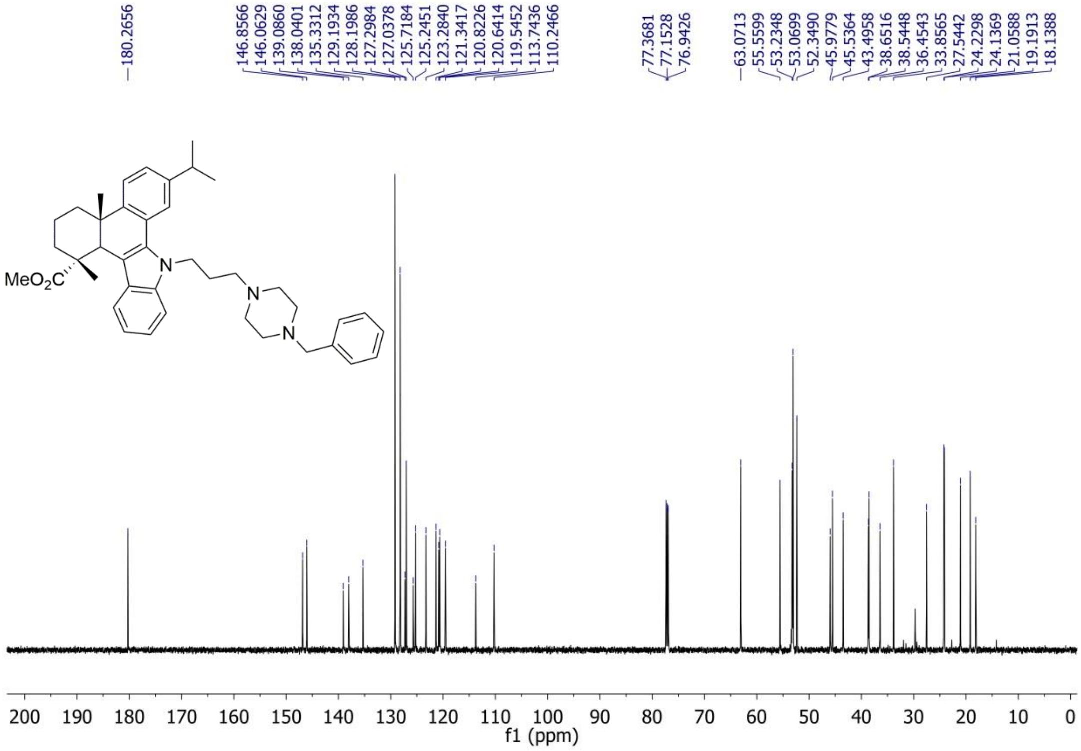

Fig. S32. ^{13}C-NMR spectrum of compound **7h** (150 MHz, $CDCl_3$)

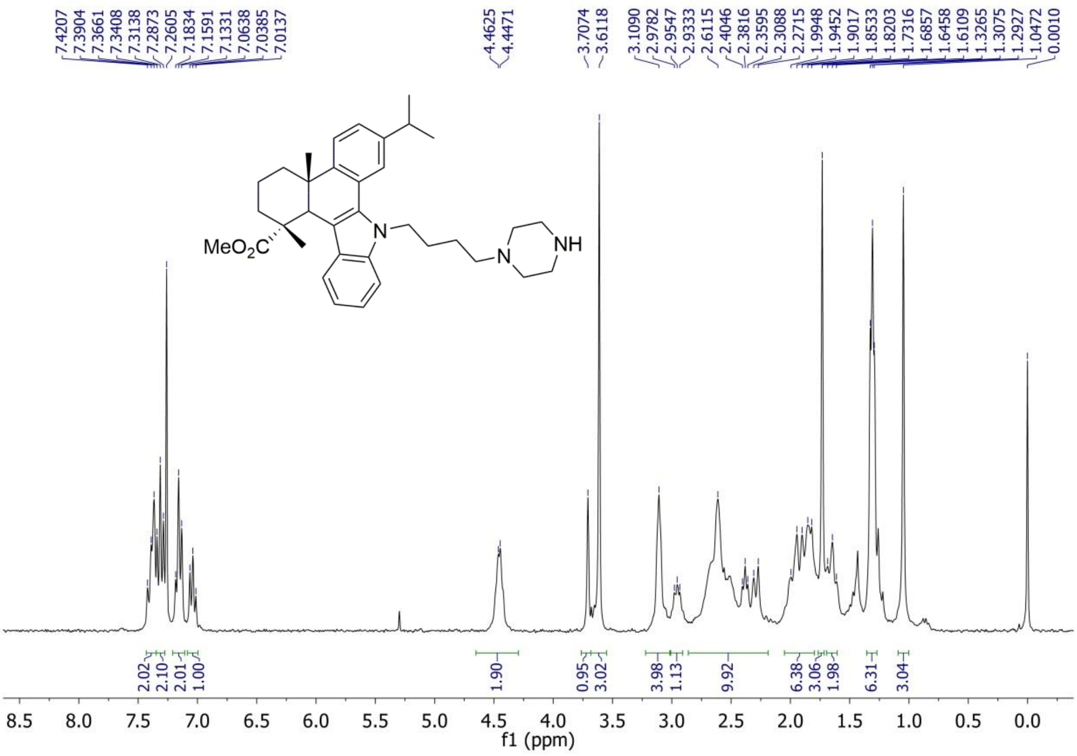

Fig. S33. ^{1}H-NMR spectrum of compound **8a** (300 MHz, $CDCl_3$)

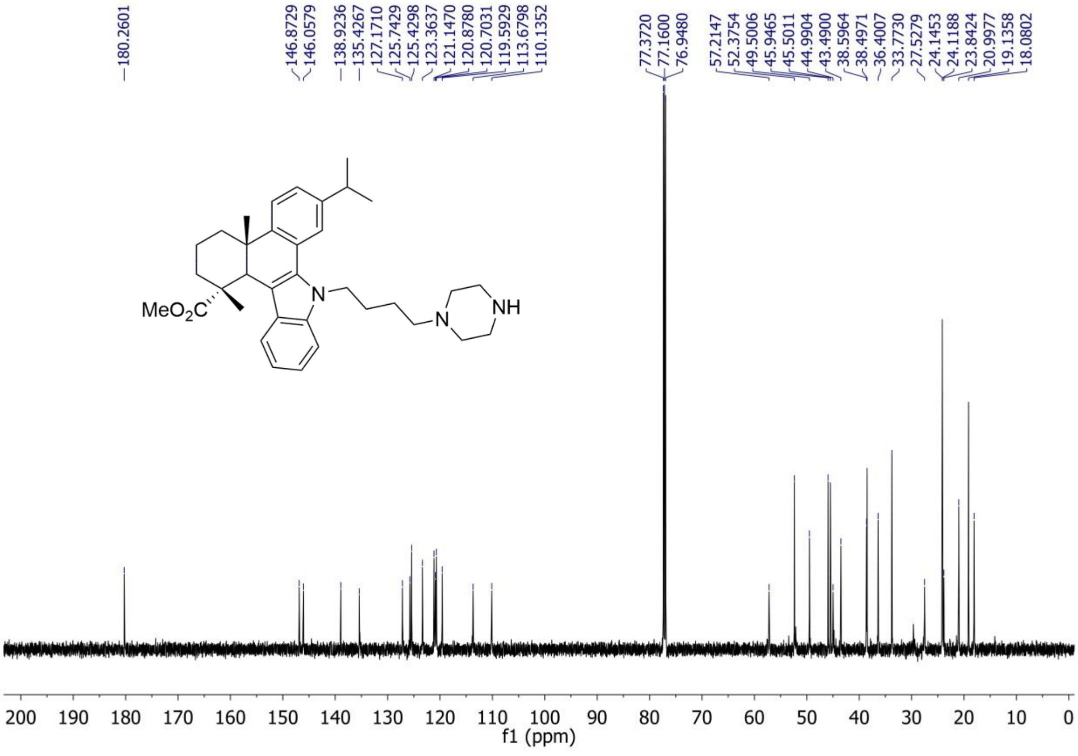

Fig. S34. ^{13}C-NMR spectrum of compound **8a** (150 MHz, $CDCl_3$)

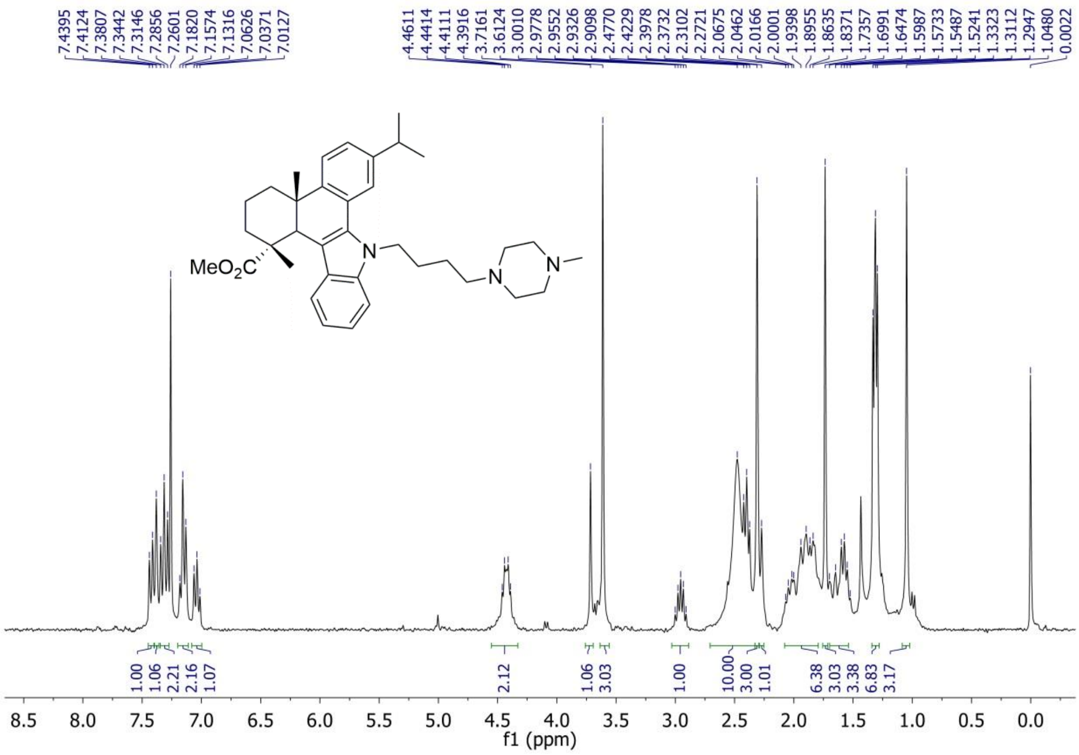

Fig. S35. ^{1}H-NMR spectrum of compound **8b** (300 MHz, $CDCl_3$)

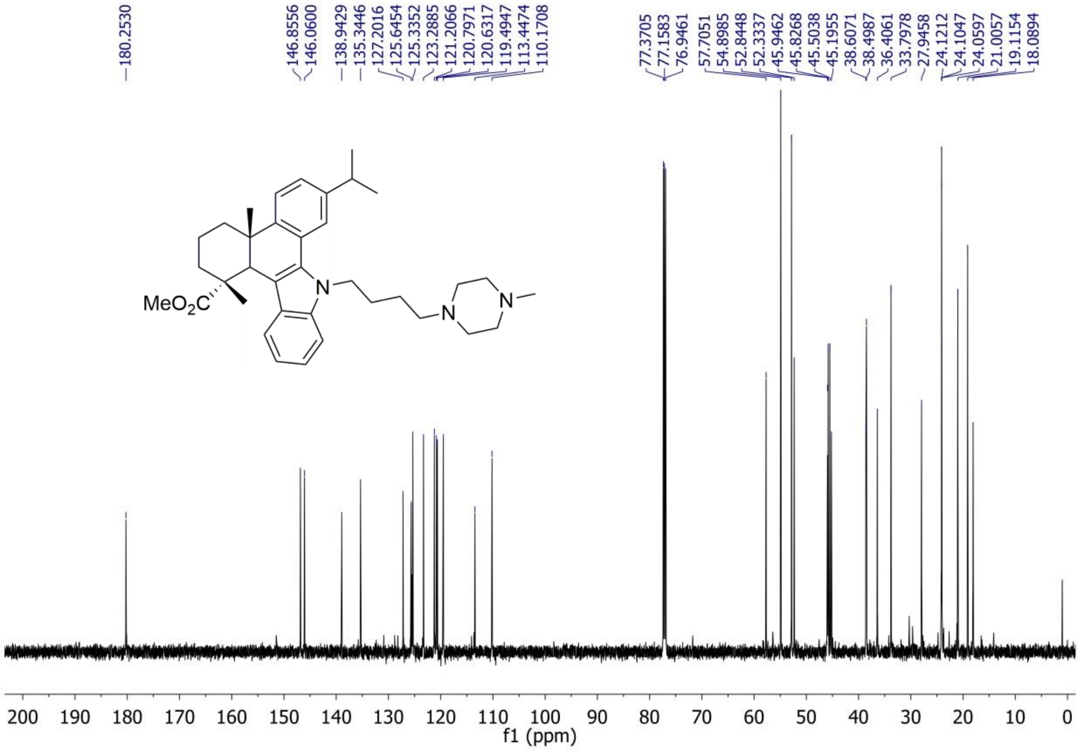

Fig. S36. ^{13}C-NMR spectrum of compound **8b** (150 MHz, $CDCl_3$)

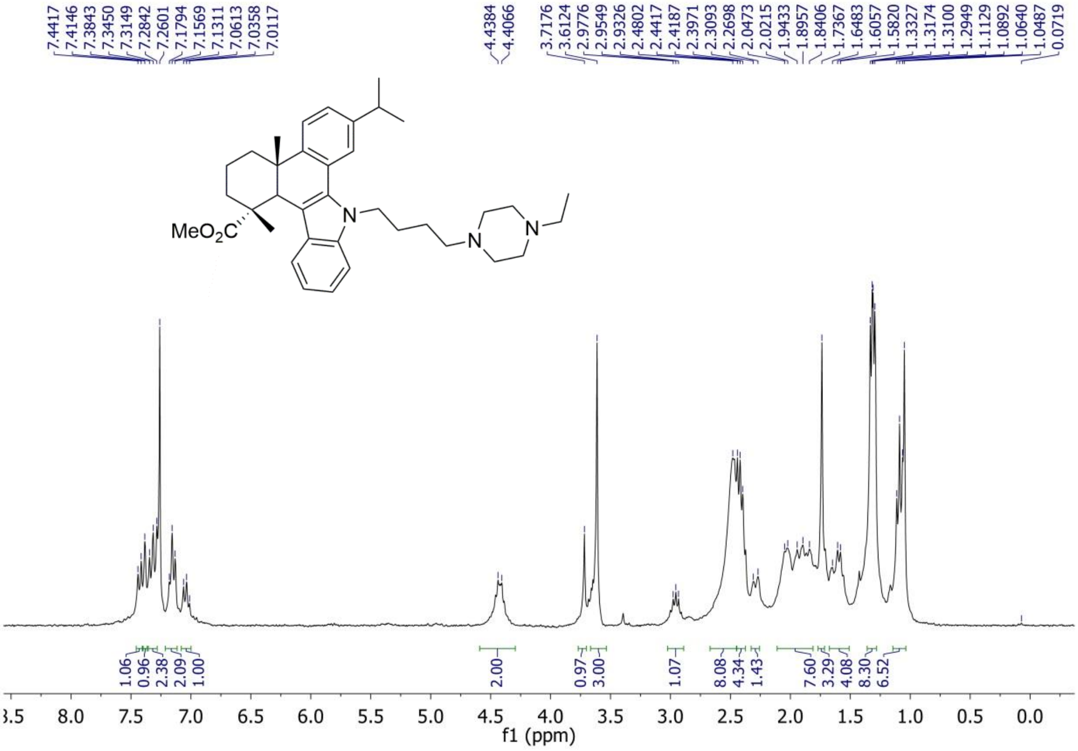

Fig. S37. ^{1}H-NMR spectrum of compound **8c** (300 MHz, $CDCl_3$)

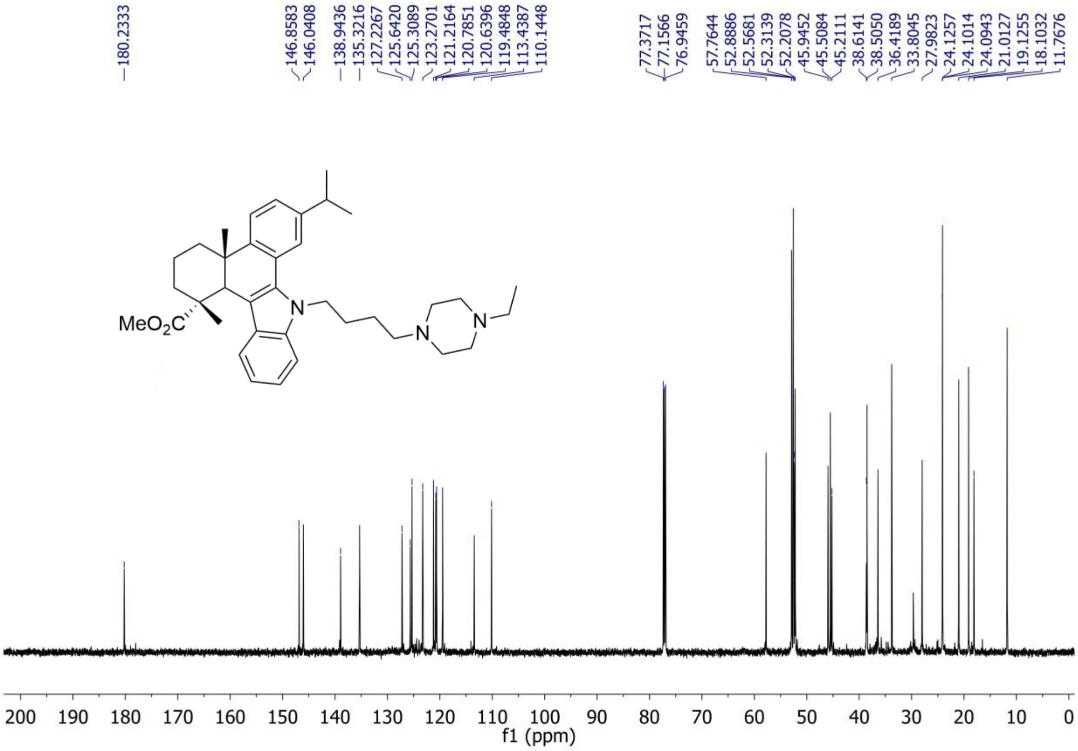

Fig. S38. ^{13}C-NMR spectrum of compound **8c** (150 MHz, $CDCl_3$)

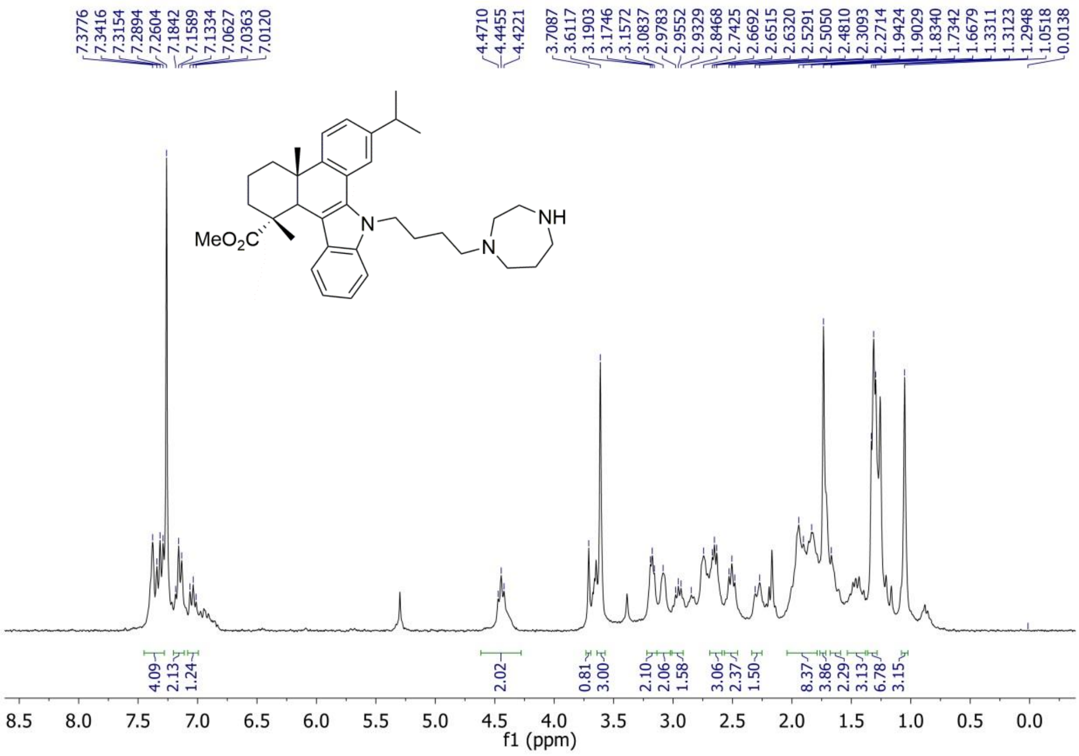

Fig. S39. ^{1}H-NMR spectrum of compound **8d** (300 MHz, $CDCl_3$)

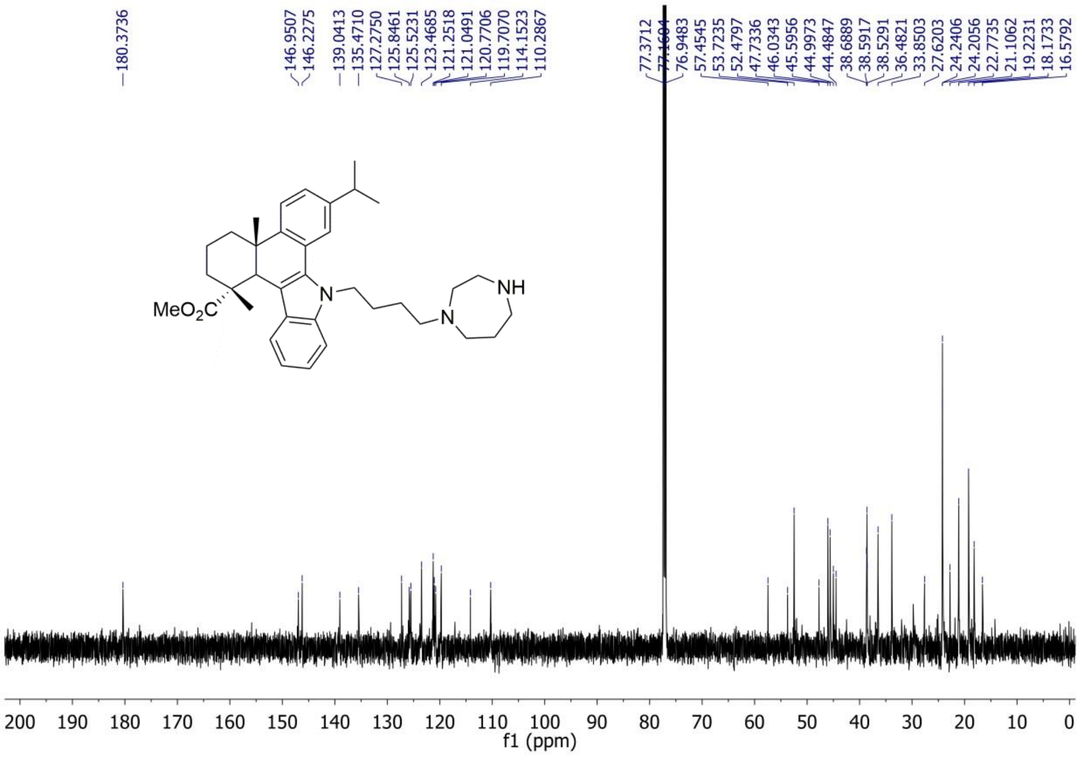

Fig. S40. ^{13}C-NMR spectrum of compound **8d** (150 MHz, $CDCl_3$)

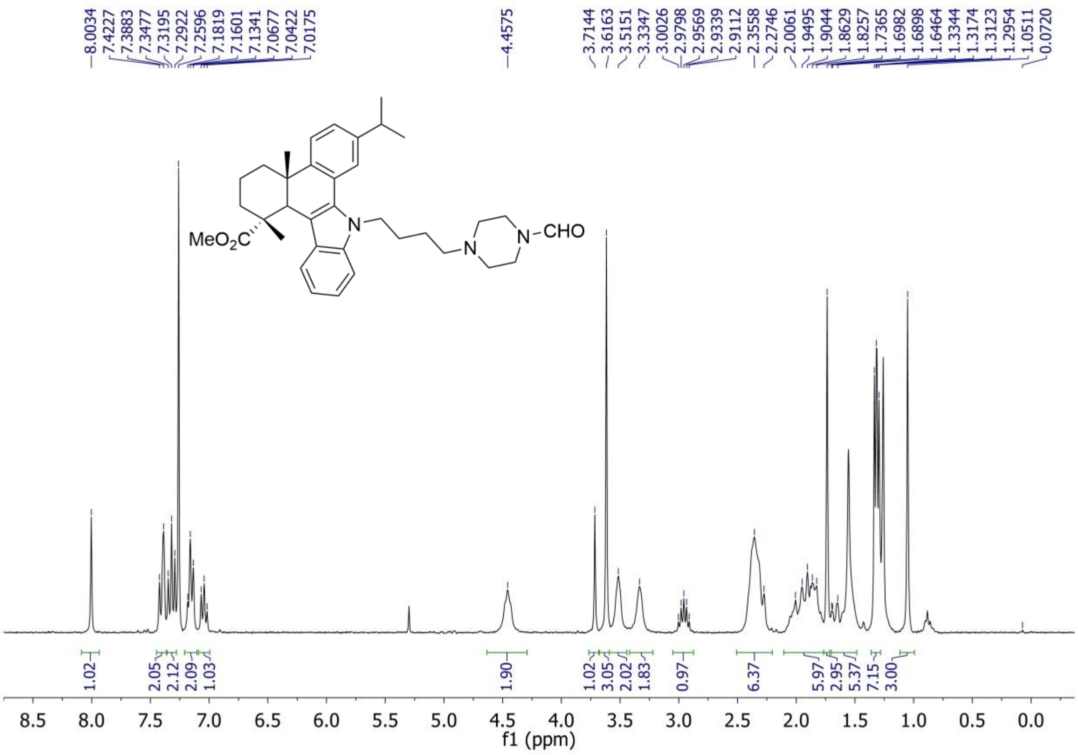

Fig. S41. ^{1}H-NMR spectrum of compound **8e** (300 MHz, $CDCl_3$)

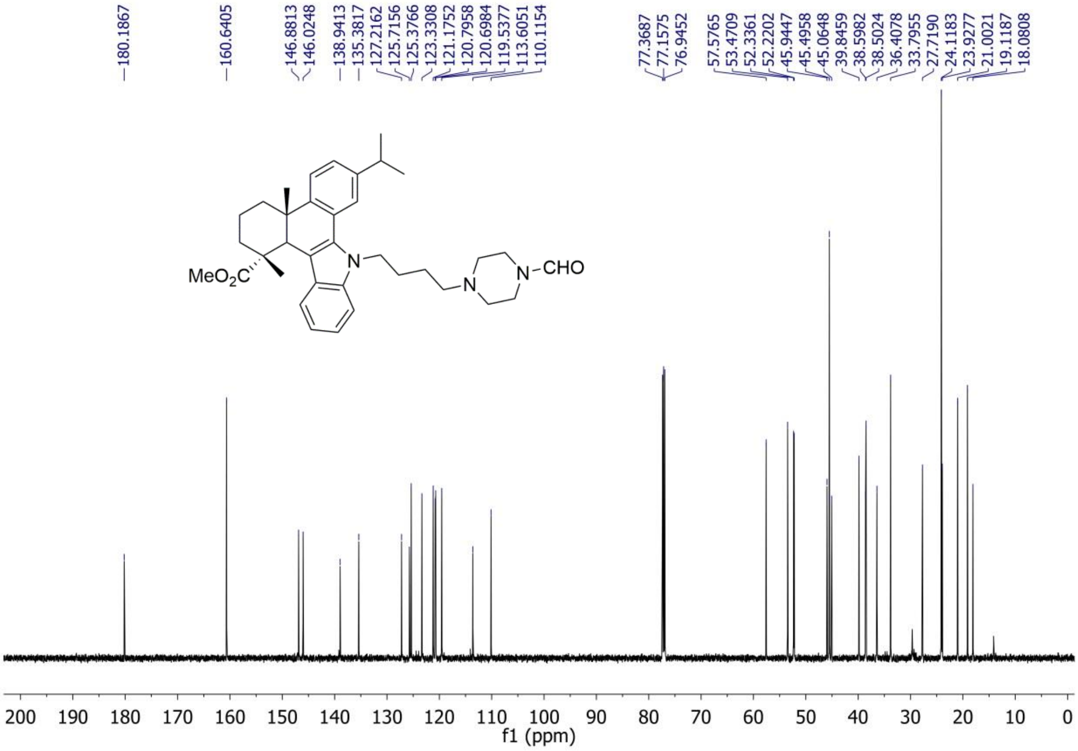

Fig. S42. ^{13}C-NMR spectrum of compound **8e** (150 MHz, $CDCl_3$)

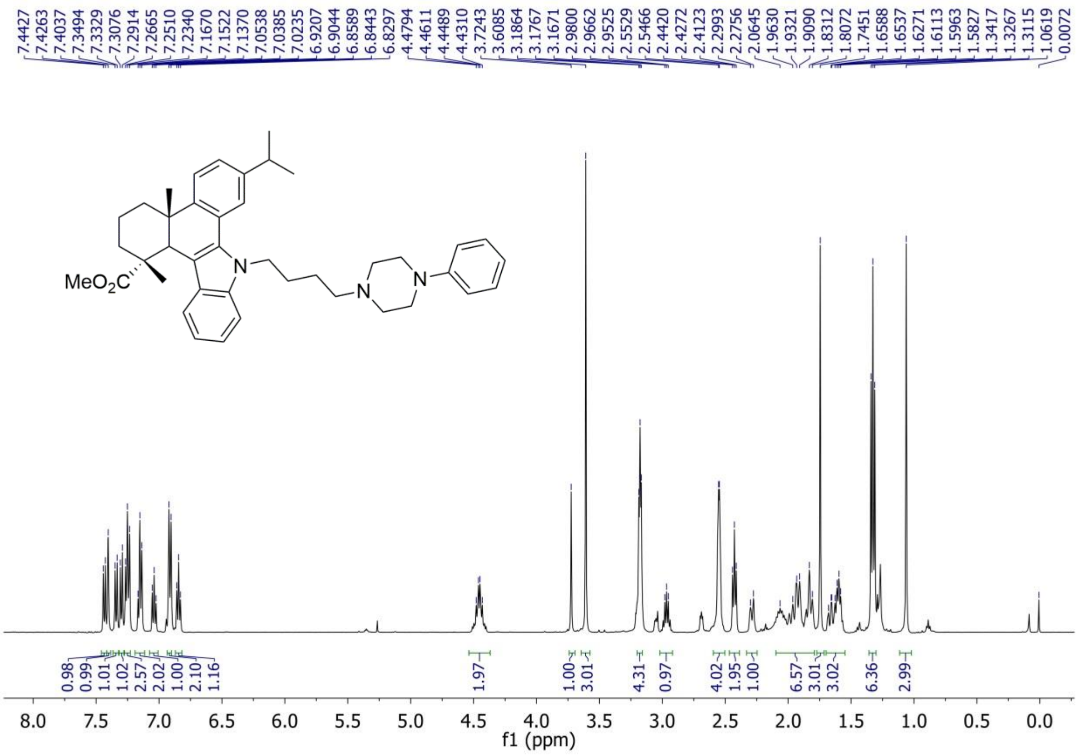

Fig. S43. ^{1}H-NMR spectrum of compound **8f** (500 MHz, $CDCl_3$)

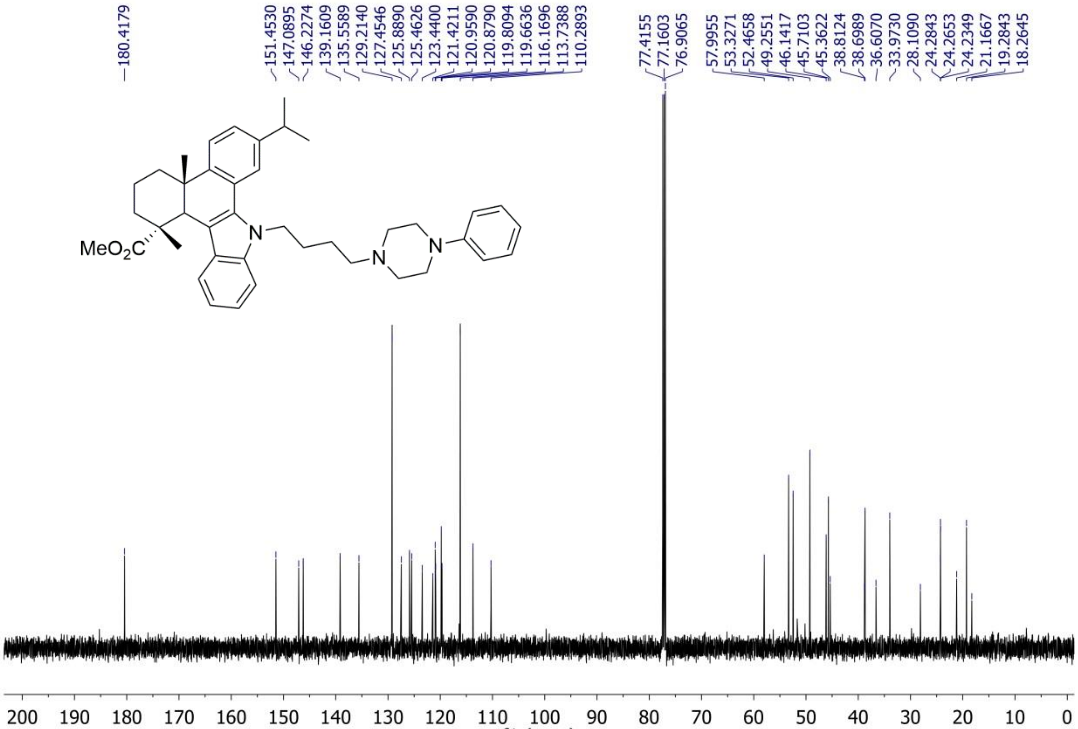

Fig. S44. ^{13}C-NMR spectrum of compound **8f** (125 MHz, $CDCl_3$)

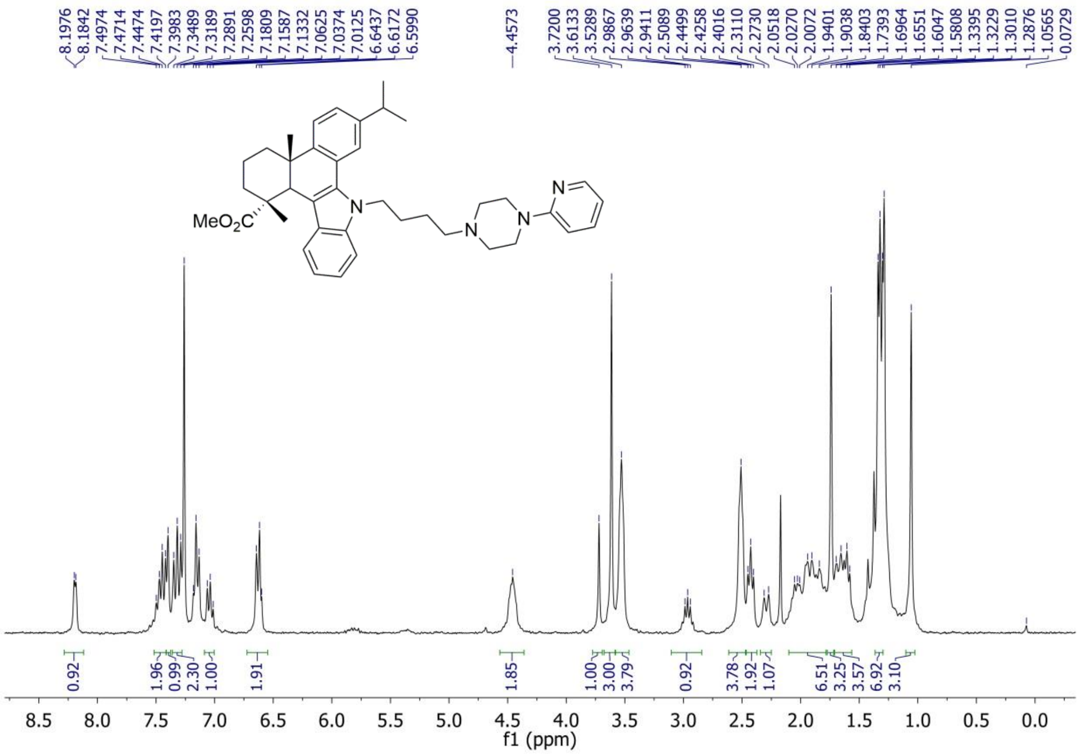

Fig. S45. ^{1}H-NMR spectrum of compound **8g** (300 MHz, $CDCl_3$)

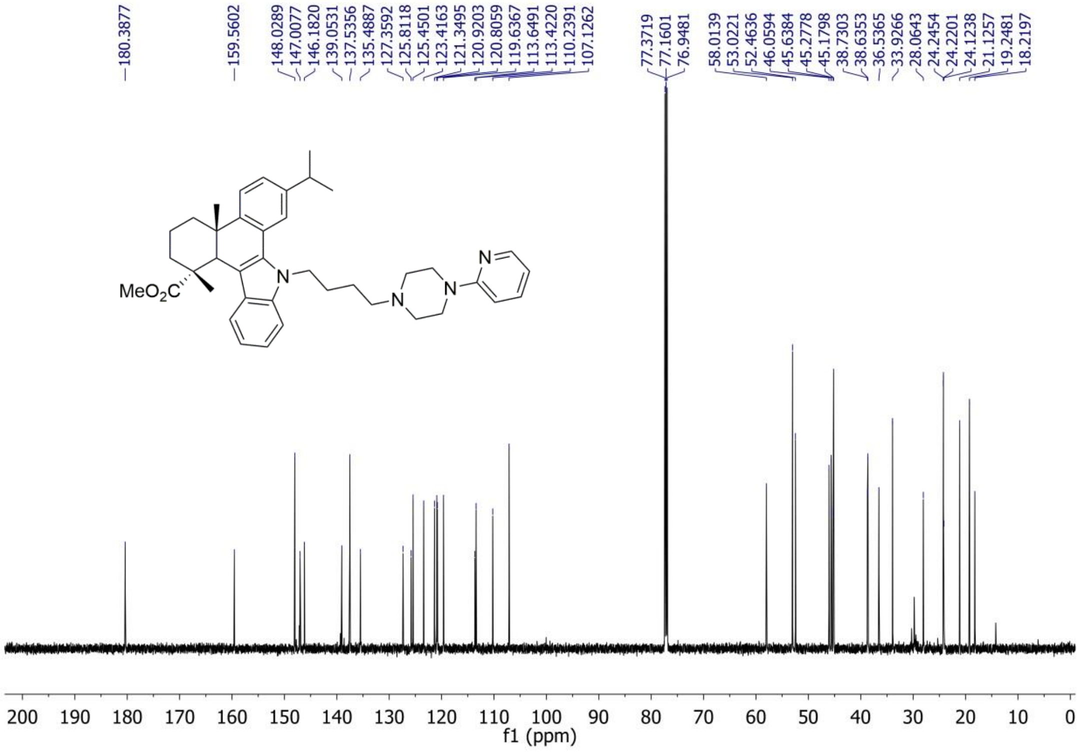

Fig. S46. ^{13}C-NMR spectrum of compound **8g** (150 MHz, $CDCl_3$)

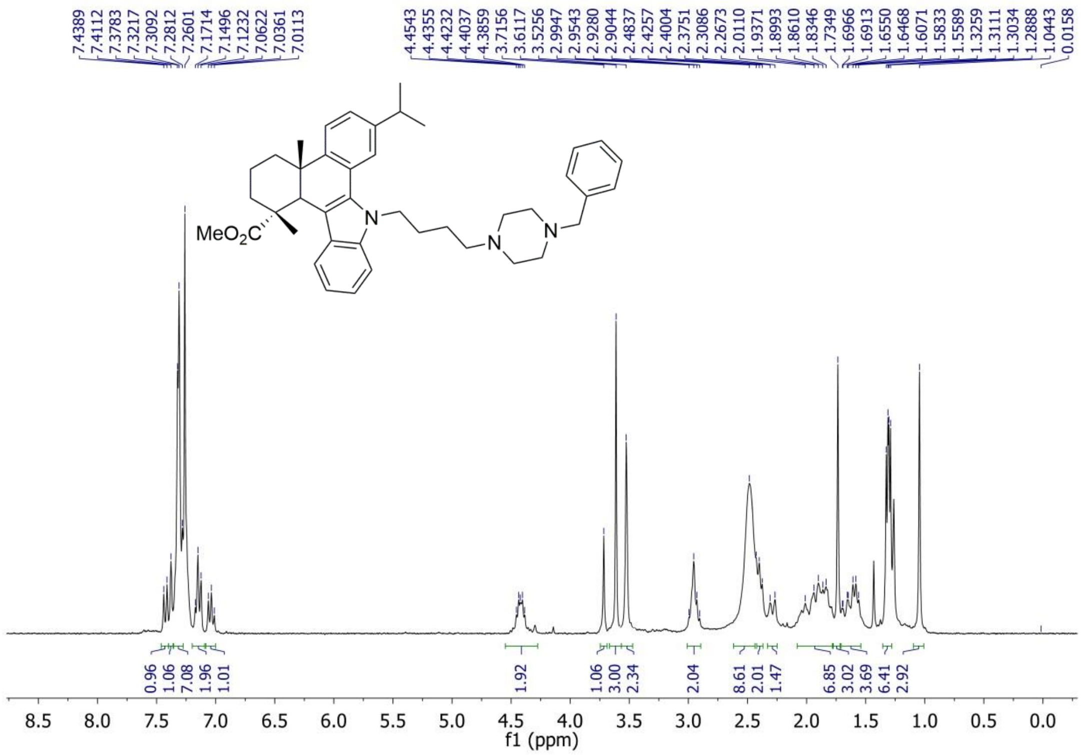

Fig. S47. ^{1}H-NMR spectrum of compound **8h** (300 MHz, $CDCl_3$)

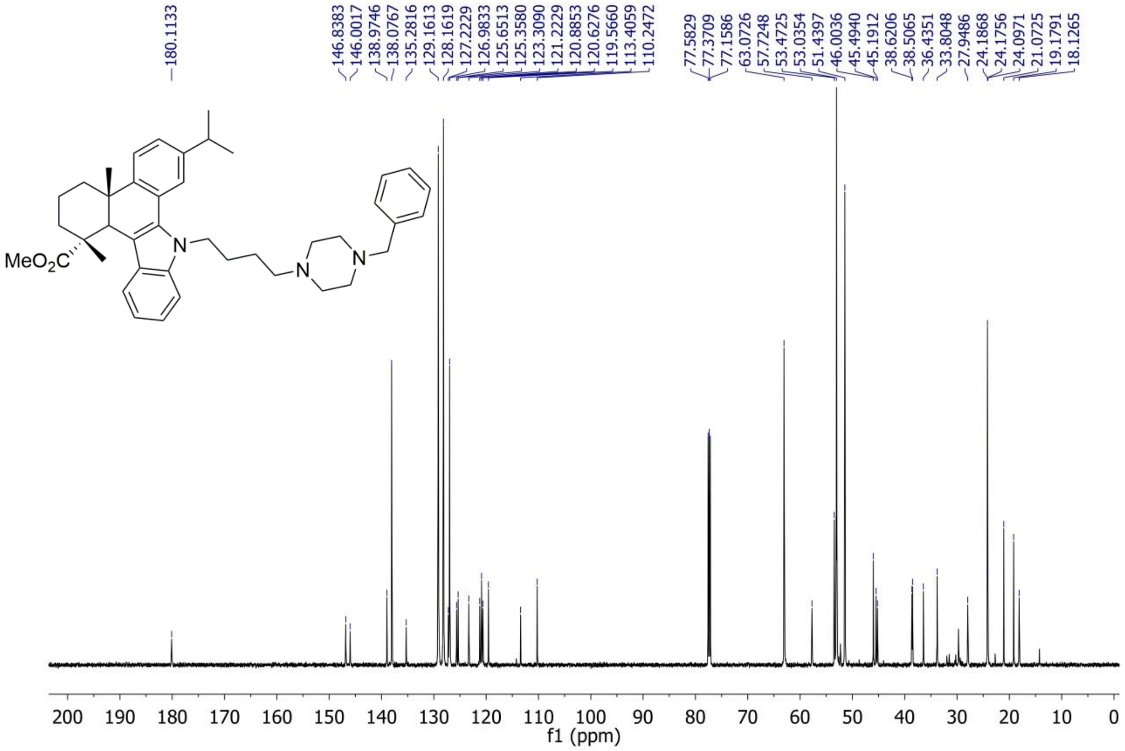

Fig. S48. ^{13}C-NMR spectrum of compound **8h** (150 MHz, $CDCl_3$)

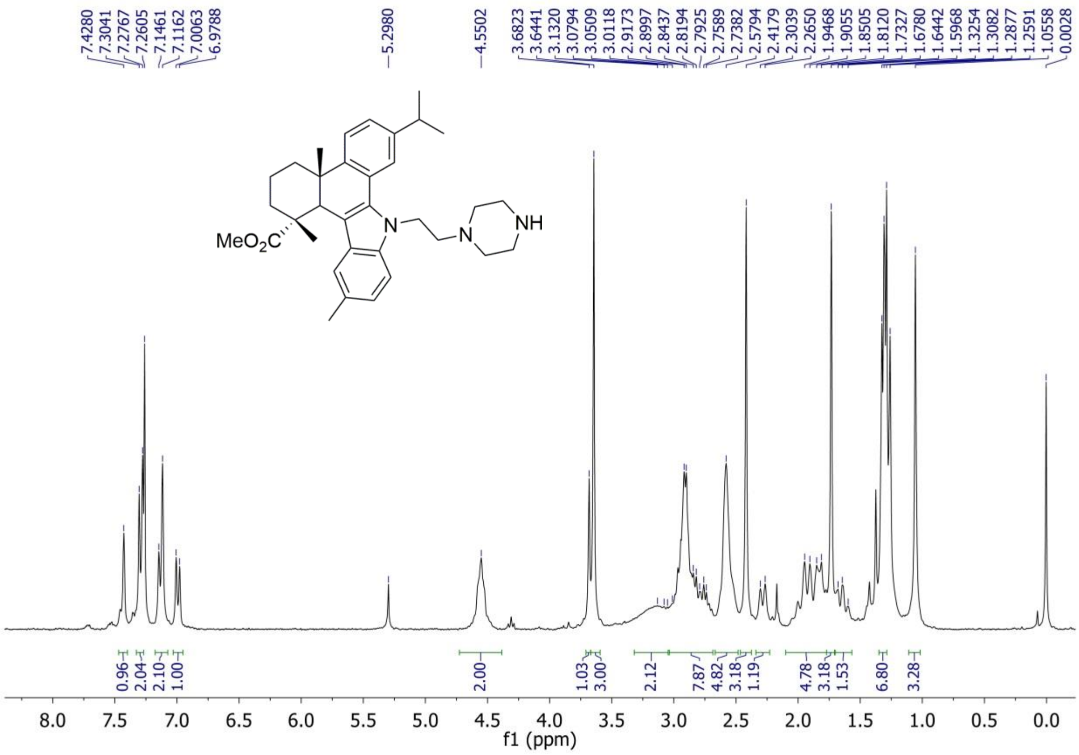

Fig. S49. ^{1}H-NMR spectrum of compound **10a** (300 MHz, $CDCl_3$)

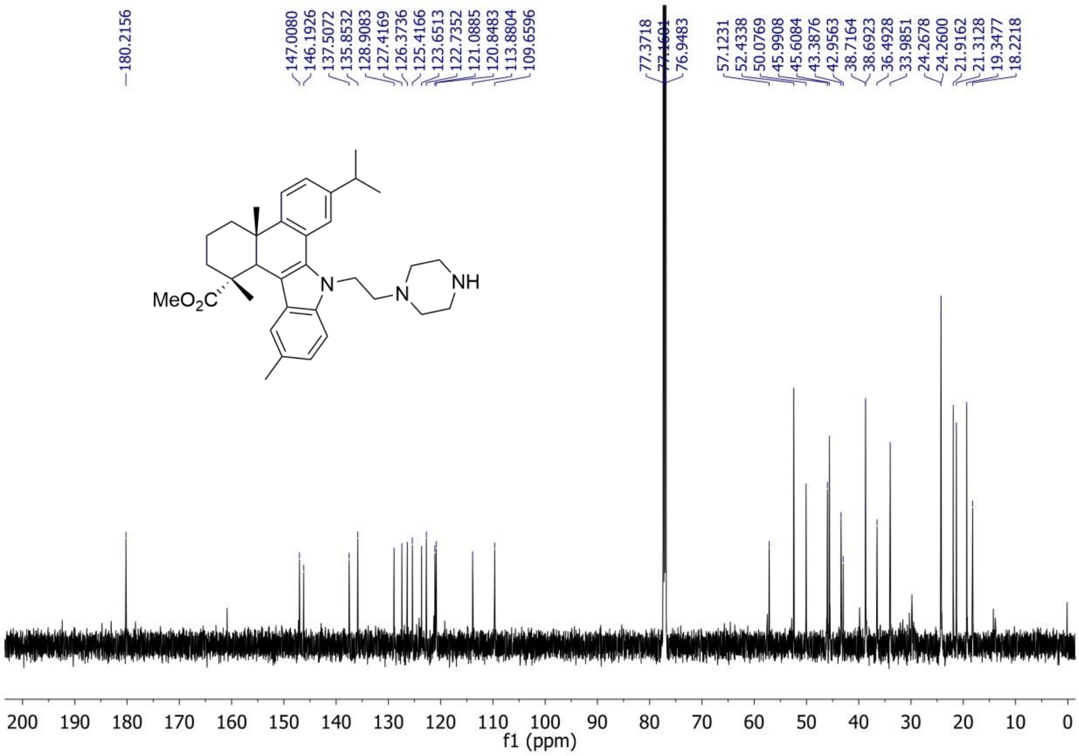

Fig. S50. ^{13}C-NMR spectrum of compound **10a** (150 MHz, $CDCl_3$)

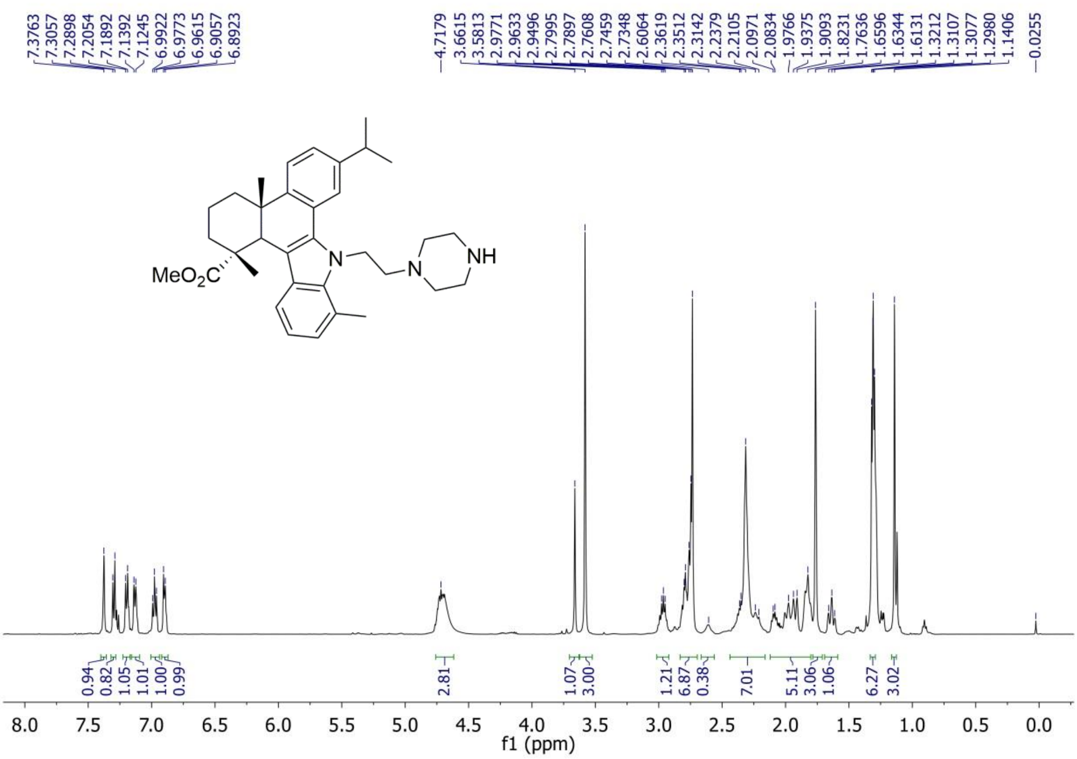

Fig. S51. ^{1}H-NMR spectrum of compound **10b** (500 MHz, $CDCl_3$)

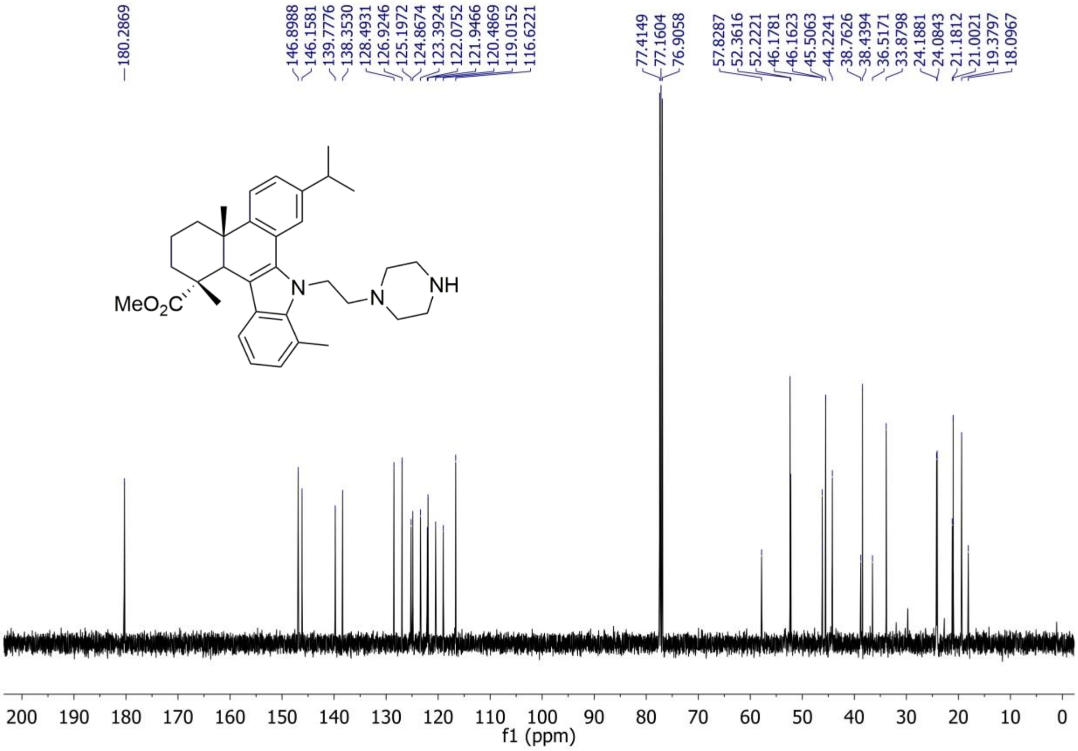

Fig. S52. ^{13}C-NMR spectrum of compound **10b** (125 MHz, $CDCl_3$)

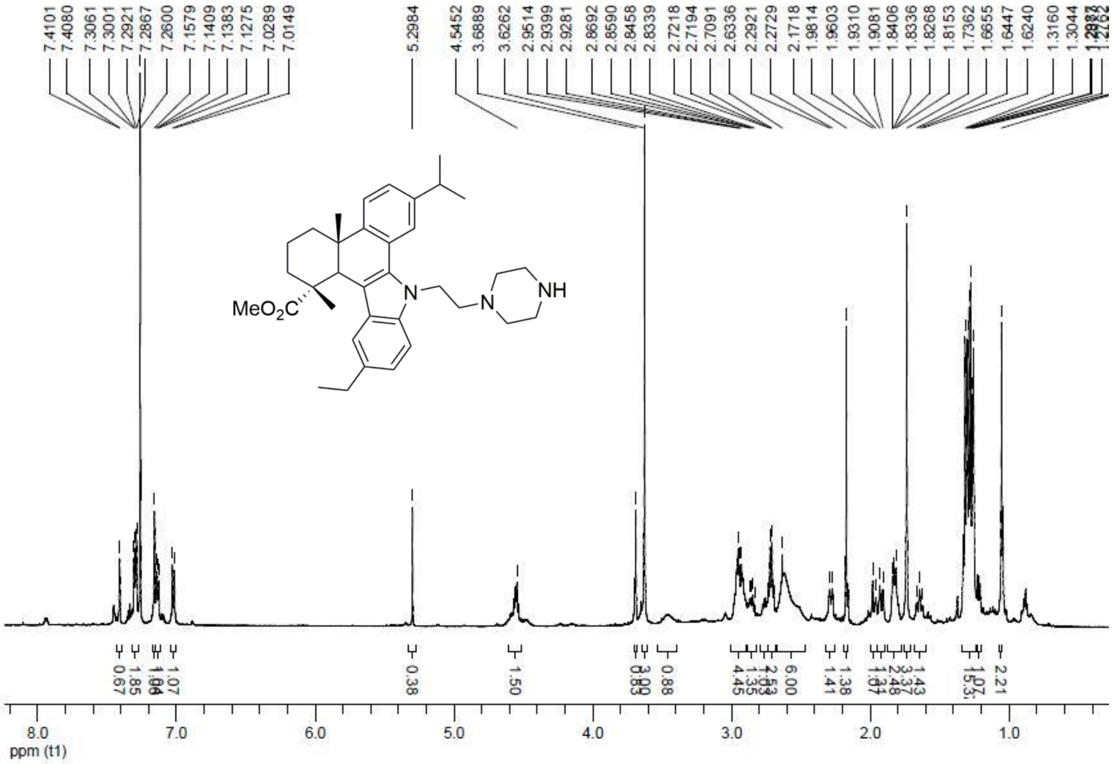

Fig. S53. ^{1}H-NMR spectrum of compound **10c** (600 MHz, $CDCl_3$)

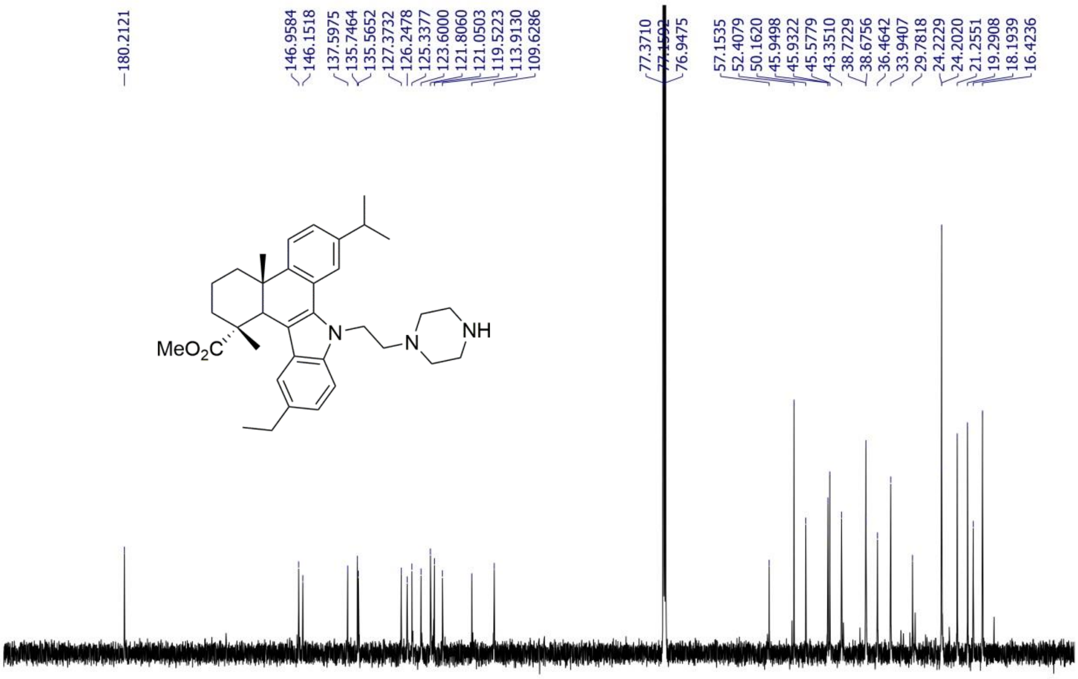

Fig. S54. ^{13}C-NMR spectrum of compound **10c** (150 MHz, $CDCl_3$)

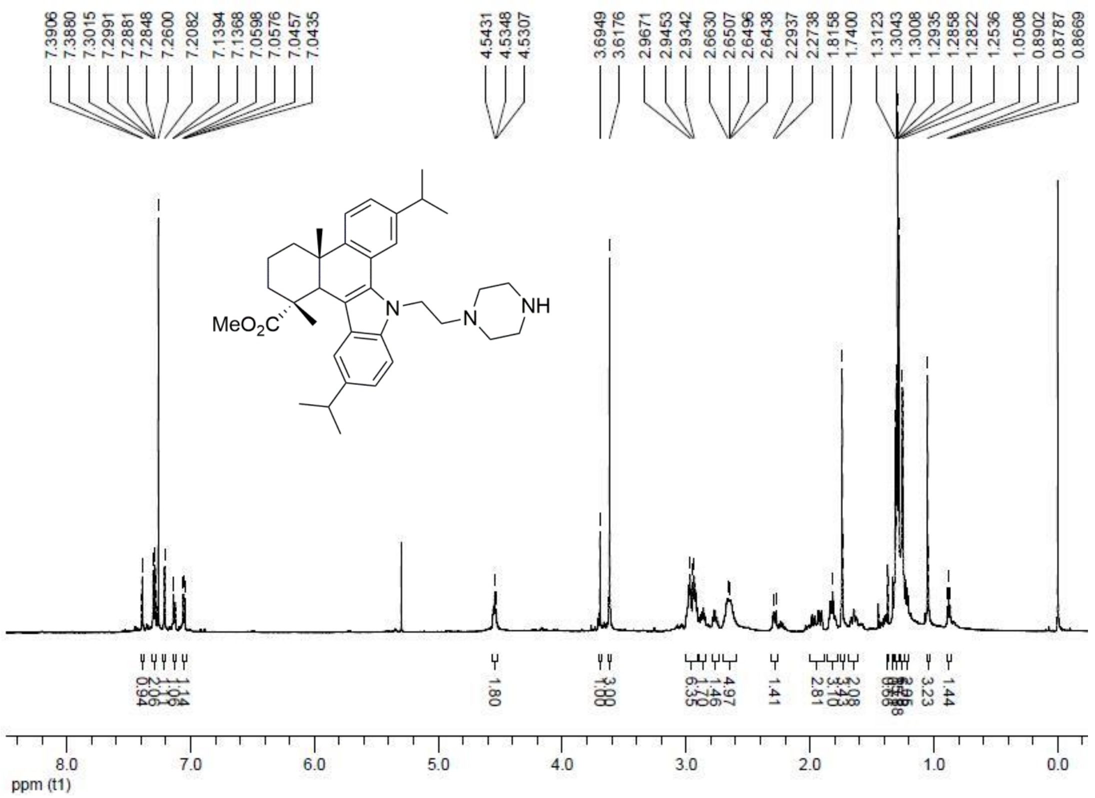

Fig. S55. ^{1}H-NMR spectrum of compound **10d** (600 MHz, $CDCl_3$)

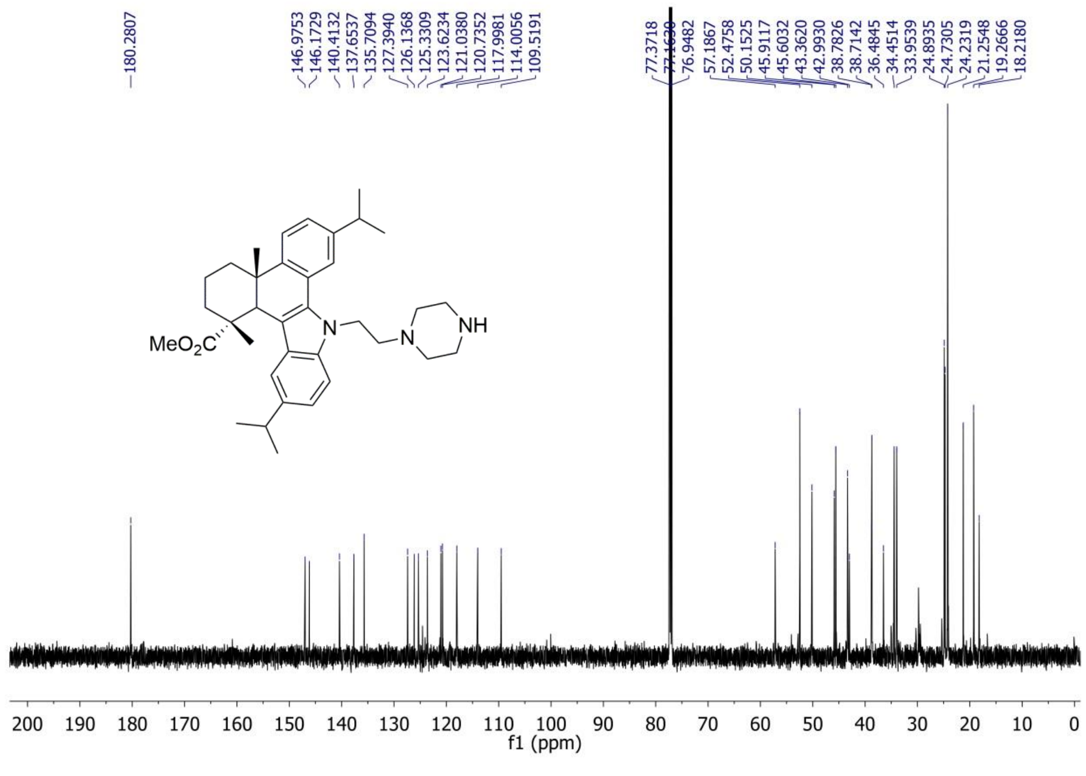

Fig. S56. ^{13}C-NMR spectrum of compound **10d** (150 MHz, $CDCl_3$)

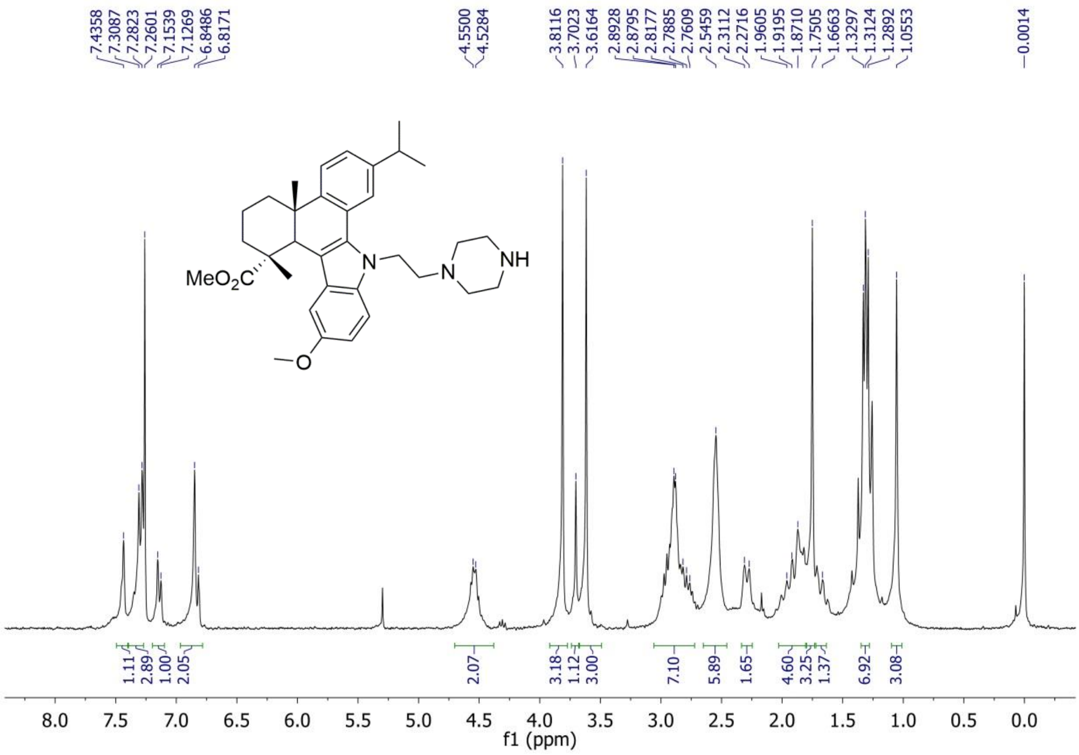

Fig. S57. ^{1}H-NMR spectrum of compound **10e** (300 MHz, $CDCl_3$)

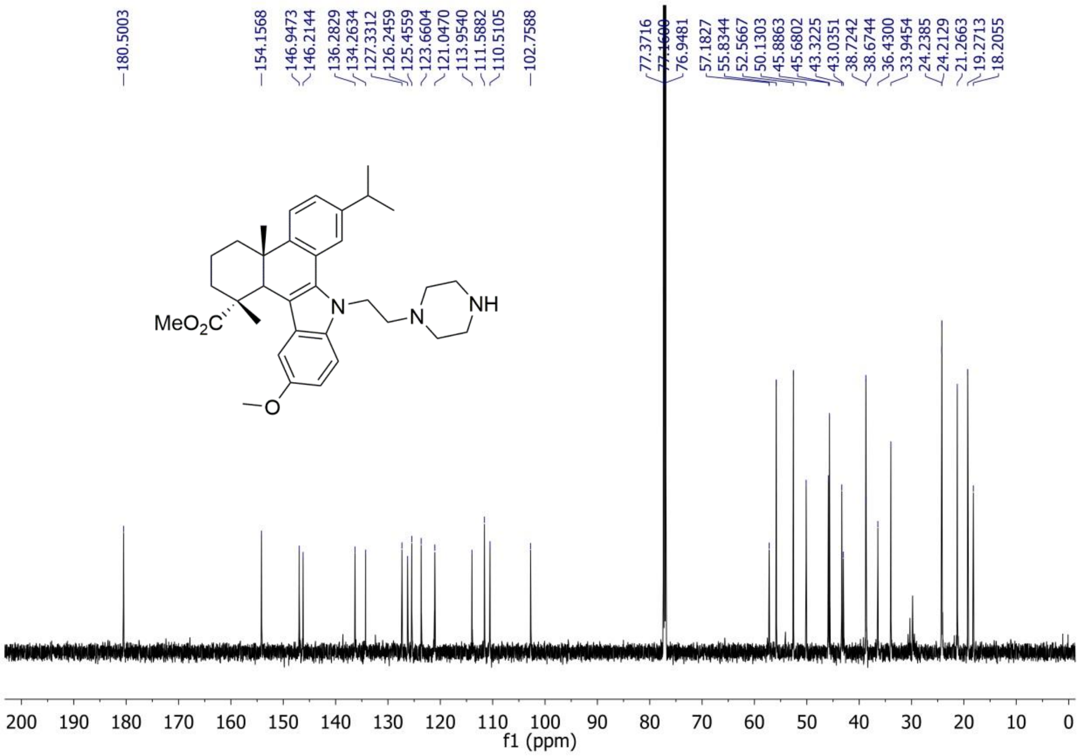

Fig. S58. ^{13}C-NMR spectrum of compound **10e** (150 MHz, $CDCl_3$)

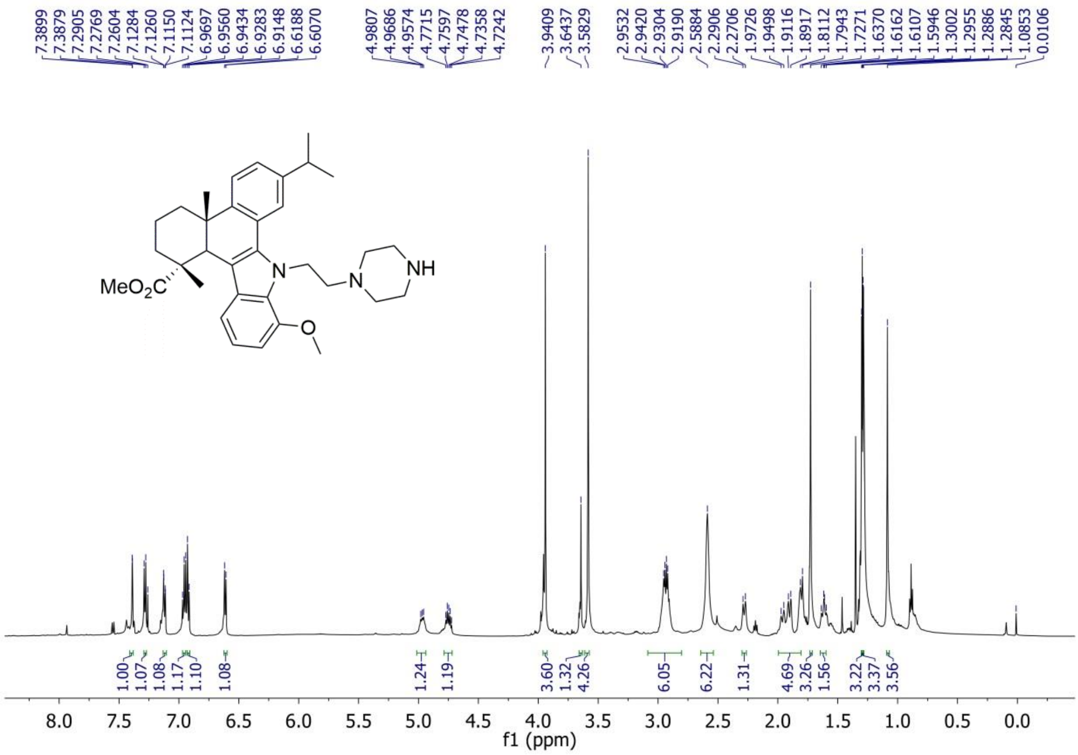

Fig. S59. ^{1}H-NMR spectrum of compound **10f** (600 MHz, $CDCl_3$)

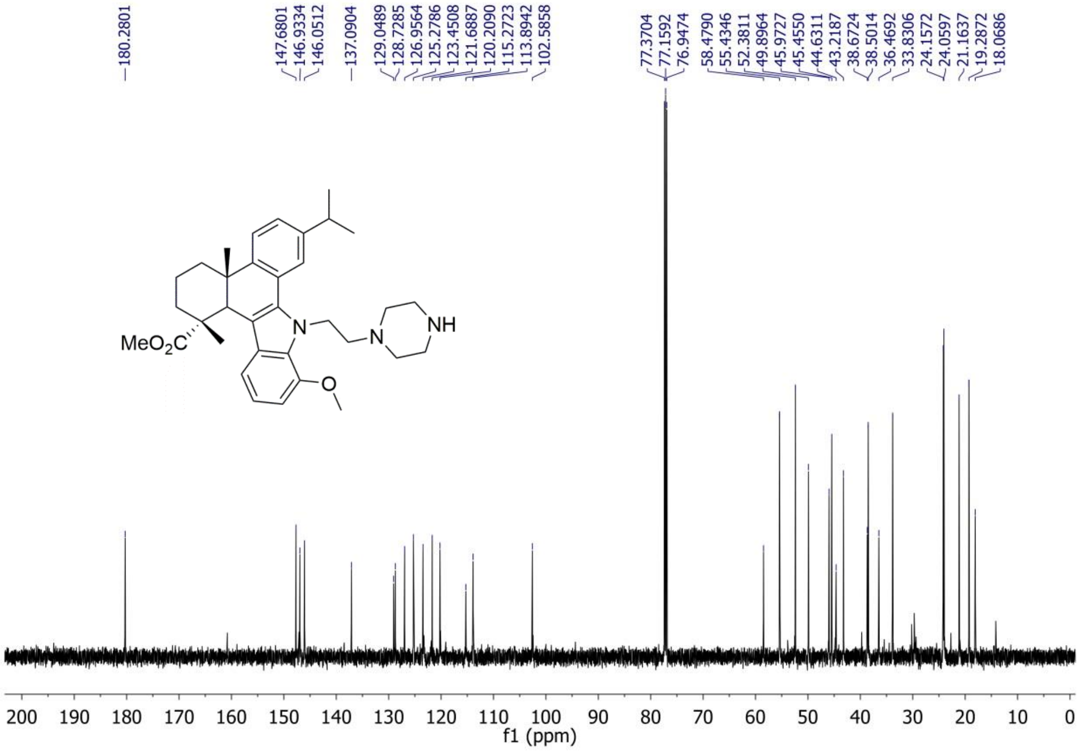

Fig. S60. ^{13}C-NMR spectrum of compound **10f** (150 MHz, $CDCl_3$)

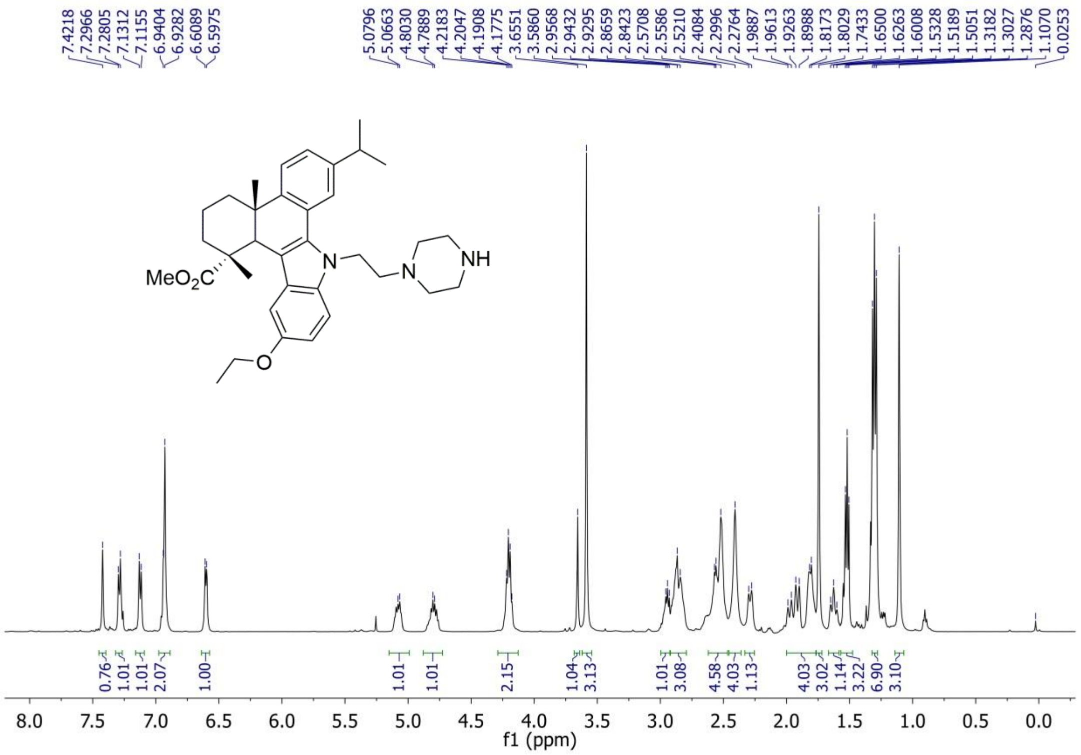

Fig. S61. ^{1}H-NMR spectrum of compound **10g** (500 MHz, $CDCl_3$)

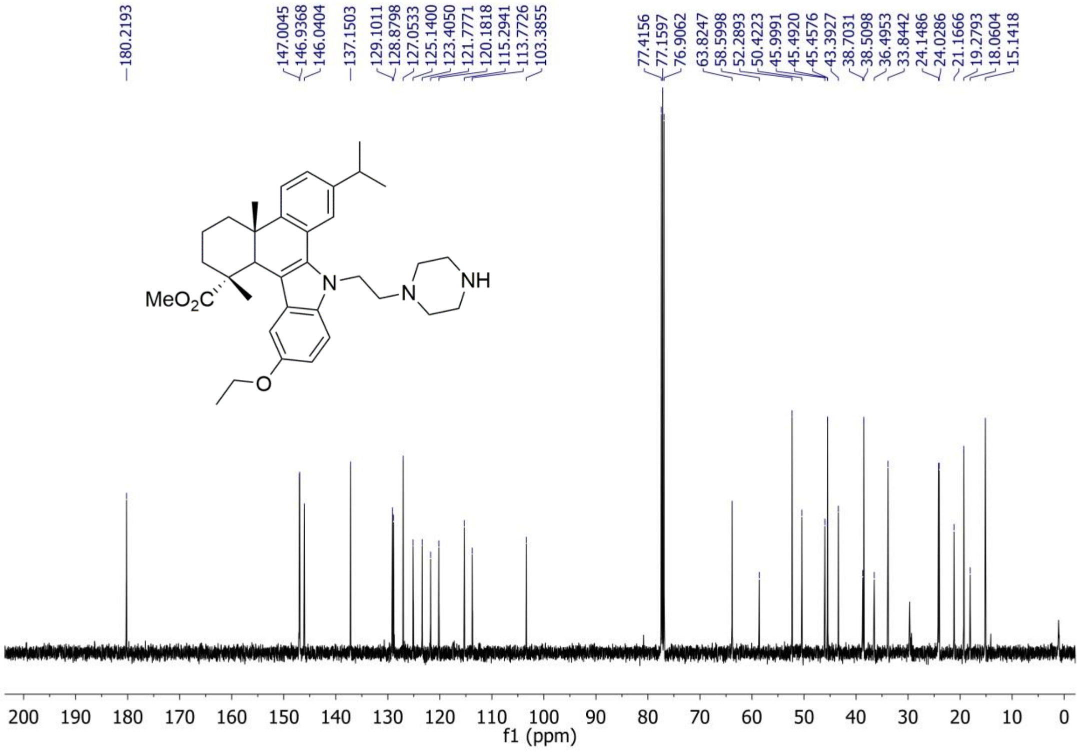

Fig. S62. ^{13}C-NMR spectrum of compound **10g** (125 MHz, $CDCl_3$)

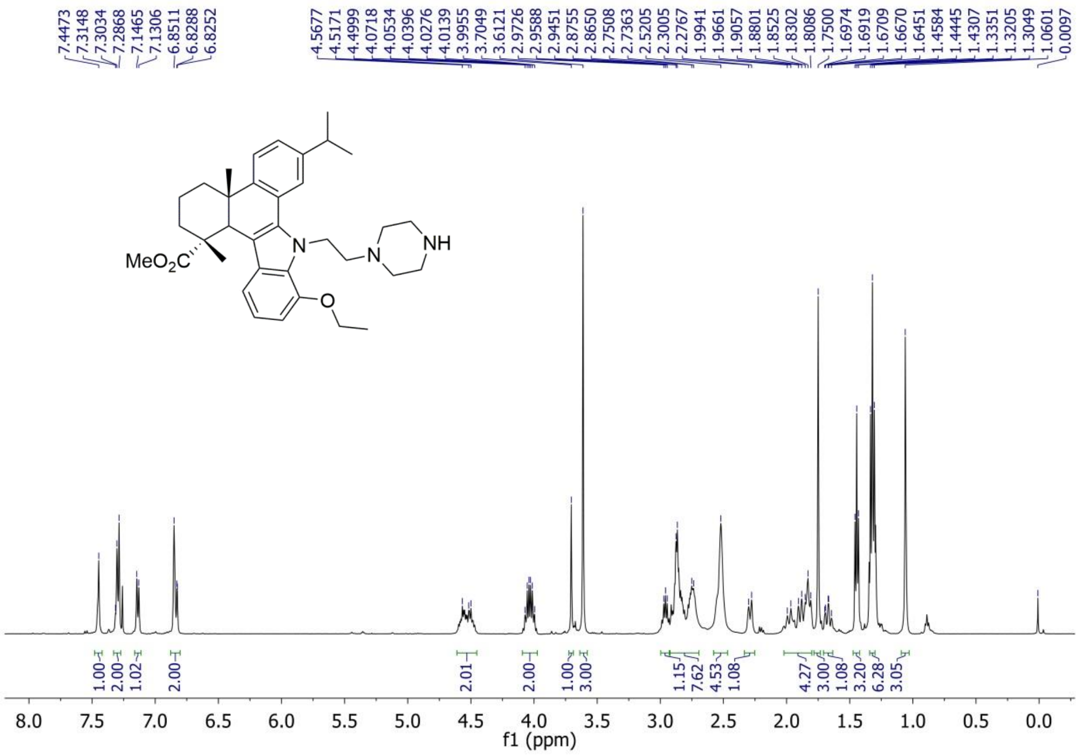

Fig. S63. ^{1}H-NMR spectrum of compound **10h** (500 MHz, $CDCl_3$)

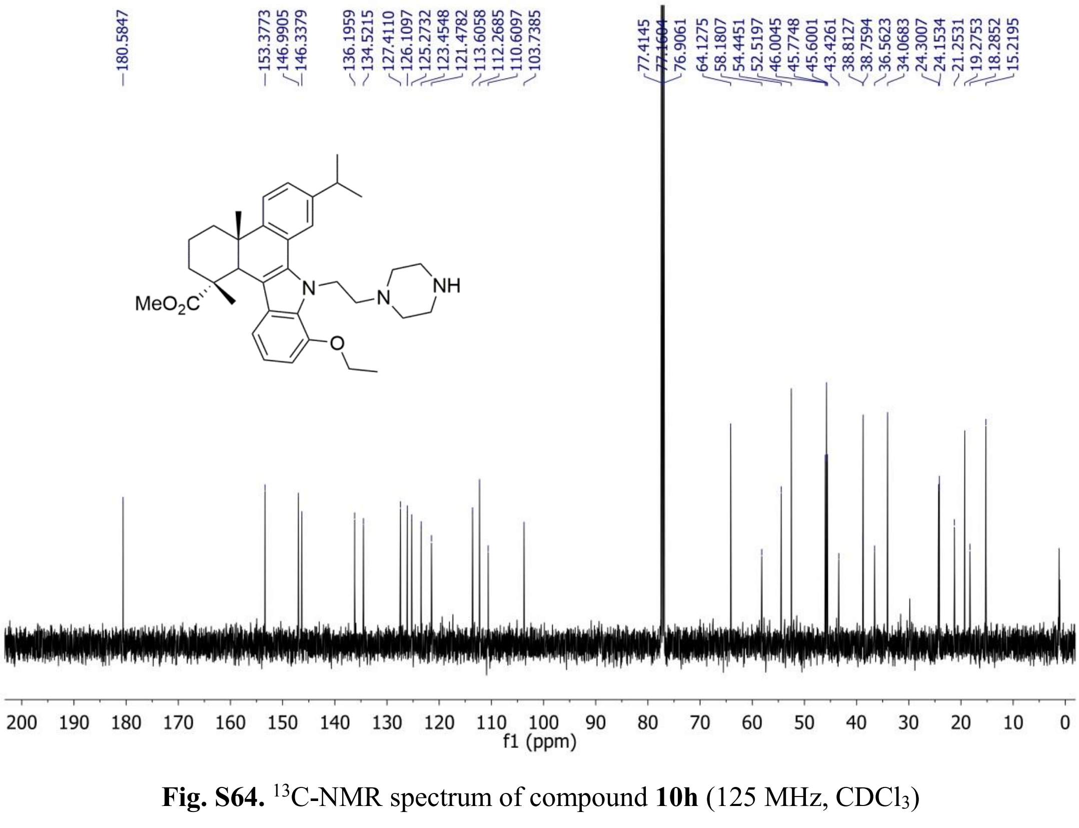

Fig. S64. ^{13}C-NMR spectrum of compound **10h** (125 MHz, $CDCl_3$)

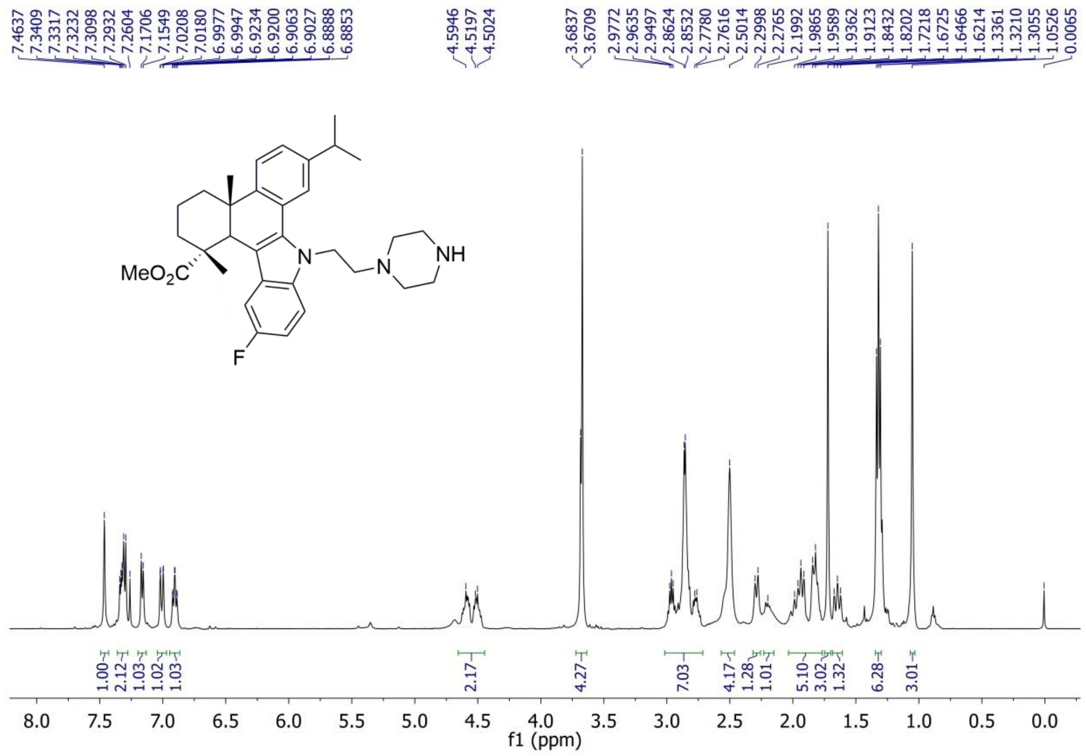

Fig. S65. ^{1}H-NMR spectrum of compound **10i** (500 MHz, $CDCl_3$)

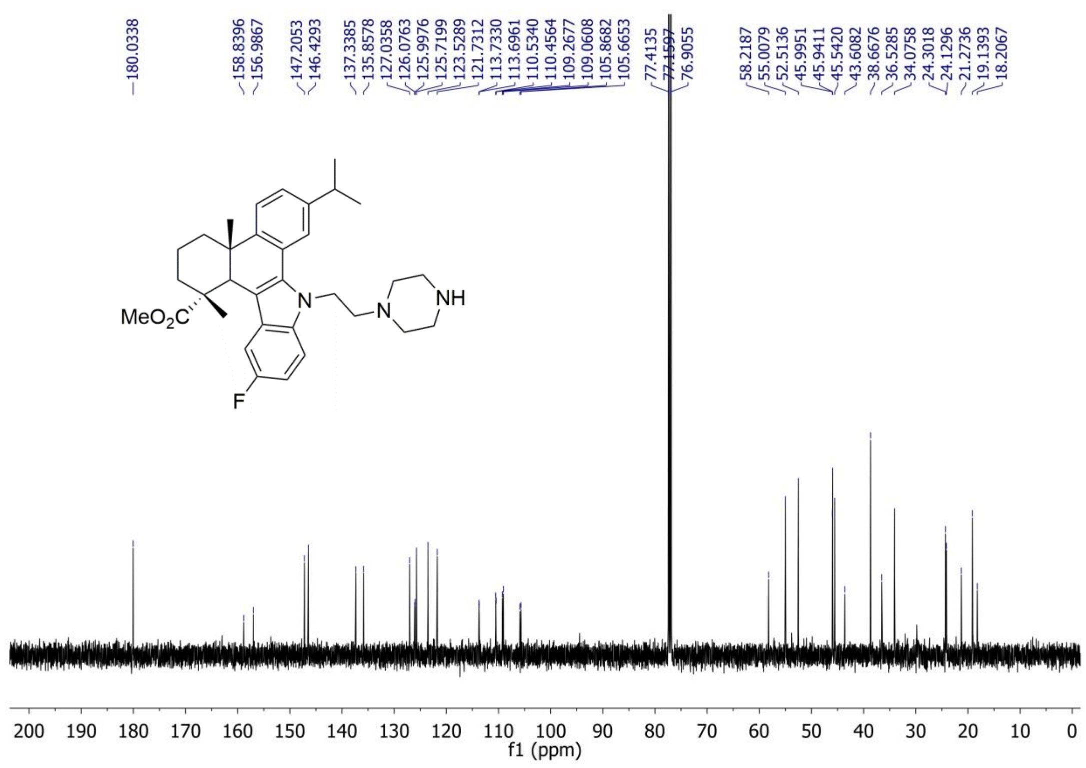

Fig. S66. ^{13}C-NMR spectrum of compound **10i** (125 MHz, $CDCl_3$)

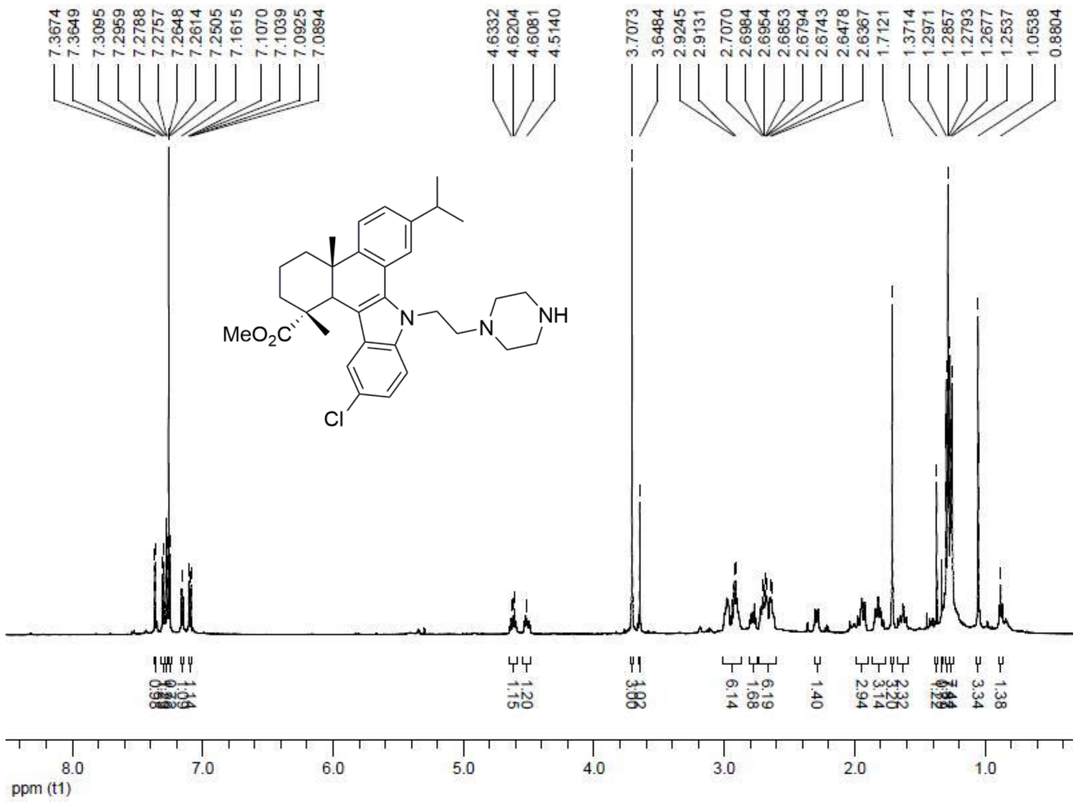

Fig. S67. ^{1}H-NMR spectrum of compound **10j** (600 MHz, $CDCl_3$)

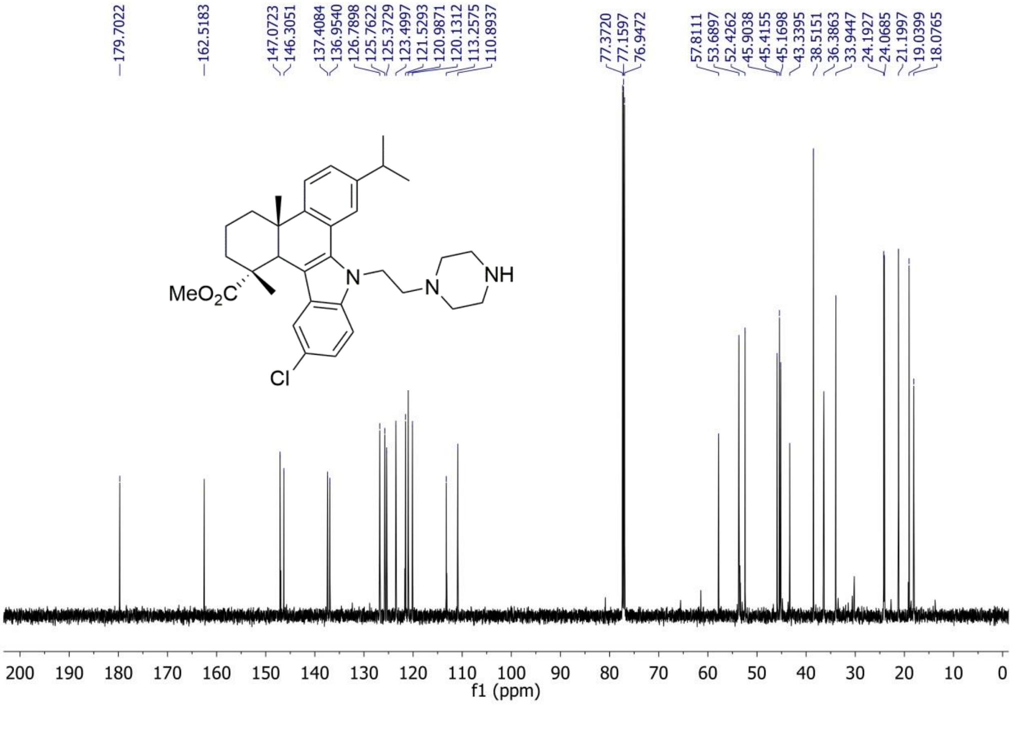

Fig. S68. ^{13}C-NMR spectrum of compound **10j** (150 MHz, $CDCl_3$)
